# The Vaccinia virion: Filling the gap between atomic and ultrastructure

**DOI:** 10.1371/journal.ppat.1007508

**Published:** 2019-01-07

**Authors:** Yeva Mirzakhanyan, Paul Gershon

**Affiliations:** Department of Molecular Biology & Biochemistry, UC-Irvine, Irvine, California, United States of America; Princeton University, UNITED STATES

## Abstract

We have investigated the molecular-level structure of the Vaccinia virion *in situ* by protein-protein chemical crosslinking, identifying 4609 unique-mass crosslink ions at an effective FDR of 0.33%, covering 2534 unique pairs of crosslinked protein positions, 625 of which were inter-protein. The data were statistically non-random and rational in the context of known structures, and showed biological rationality. Crosslink density strongly tracked the individual proteolytic maturation products of p4a and p4b, the two major virion structural proteins, and supported the prediction of transmembrane domains within membrane proteins. A clear sub-network of four virion structural proteins provided structural insights into the virion core wall, and proteins VP8 and A12 formed a strongly-detected crosslinked pair with an apparent structural role. A strongly-detected sub-network of membrane proteins A17, H3, A27 and A26 represented an apparent interface of the early-forming virion envelope with structures added later during virion morphogenesis. Protein H3 seemed to be the central hub not only for this sub-network but also for an ‘attachment protein’ sub-network comprising membrane proteins H3, ATI, CAHH(D8), A26, A27 and G9. Crosslinking data lent support to a number of known interactions and interactions within known complexes. Evidence is provided for the membrane targeting of genome telomeres. In covering several orders of magnitude in protein abundance, this study may have come close to the bottom of the protein-protein crosslinkome of an intact organism, namely a complex animal virus.

## Author summary

Vaccinia is one of the most complex virions among the animal viruses, containing 70+ distinct gene products. Although virion ultrastructure has been apparent, at least in outline by electron microscopy since the year 1961 or earlier, its molecular architecture is largely unknown: Vaccinia is resistant to classical structural approaches requiring virus crystallization and moderately resistant to cryoEM. Molecular approaches requiring the maintenance of protein assemblies during virion deconstruction, reconstruction of protein complexes in heterologous or *in vitro* systems, or internalization of bulky reagents such as antibodies or gold particles may have been already pursued close to exhaustion. Here, protein interfaces within and around the intact virion were identified by virus incubation with bifunctional chemical crosslinkers *in situ* followed by proteolysis and peptide-level mass spectrometry. This minimally invasive approach revealed the molecular arrangements of structural and membrane protein complexes within the virus, confirming and extending several aspects of virus biology.

## Introduction

The virion of Vaccinia, the prototypical poxvirus, is one of the largest among the animal viruses. While its ultrastructural characterization is the beneficiary of 60+ years of electron microscopic examination [1–3] and references therein, attempts to better understand its molecular and atomic architecture have fallen foul of various properties of the Vaccinia virion such as asymmetry, polymorphic character, tendency to aggregate, and the general incompatibility of enveloped viruses with X-ray crystallography.

Electron microscopy (EM) and atomic force microscopy (AFM) studies have established clear ultrastructural compartments of the mature virion (MV) [4] including a central, genome-containing ‘core’ that also houses a number of virus-encoded enzymes of mRNA transcription and modification, a proteinaceous wall surrounding the core, a pair of ‘lateral body’ structures flanking the core wall, a single lipid bilayer envelope, and an outer protein-rich coat that appears late during maturation. The virion contains between 58 and 73 distinct gene products [5]. Some of these have been localized at low resolution on the basis of immunogold EM [6–10], while the compartmental locale of others can be inferred from clearly identifiable transmembrane (TM) domains and other bioinformatics signatures, known function and/or the conditions required for the extraction from the virion. Proteins and visible structures localizing to outer compartments of the virion (outside of the core) have been identified via their fractionation *in vivo* during virus entry [10, 11] or under pseudo-entry conditions recreated by the gentle, controlled treatment of virions with nonionic detergent or nonionic detergent+disulfide reductant [9, 12–15]. A number of core enzymes, including the virus-encoded multisubunit DNA-dependent RNA polymerase (RPO), heterodimeric virion capping enzyme (CA), early transcription factor (ETF), poly(A) polymerase (PAP), two protein kinases, at least two proteases and two glutaredoxins have been released from the virion under more harsh conditions (0.2% ionic detergent (sarkosyl) and high salt [16]), retaining solubility, integrity and activity after detergent removal [4]. By contrast, a number of structural proteins of the virion core remain insoluble during virion extraction even in ionic detergent.

Aside from these compartmentalization approaches, little is known of the virion’s internal organization at the molecular level. Certainly, the heteromultimeric status of the above core enzymes has long been known [4], and the homomultimeric status of yet other virion proteins has been revealed by X-ray crystallography (eg. proteins H1 [17, 18] and A27 [19]). Some binary protein-protein interactions have been successfully recapitulated and identified in a yeast two-hybrid system [20]. Other proteins, and fragments thereof, have been co-immunoprecipitated from cell extracts, pulled-out as tagged complexes [2] or inferred by genetic and directed mutational studies.

However, larger macromolecular and ultrastructural assemblies clearly dissociate under the conditions required for full virion disruption. For example, the presence, within the virion core, of a ‘transcriptosome’ assembly was inferred in studies down-regulating the Vaccinia RNA polymerase subunit RAP94. Under non-permissive conditions, virions were morphologically mature but showed low infectivity [21]. Albeit the virus genome was packaged in normal amounts as were ETF and the structural proteins, low or undetectable amounts of RPO, CA, PAP large subunit, and proteins NTP1, RNA helicase and topoisomerase were packaged suggesting the coordinated packaging of the latter components. Such a ‘transcriptosome’ complex may correspond to the formation, within the core, of a genome-containing tubular ultrastructure [22] that can be resolved by EM under sample preparation conditions that include high pressure freezing [23]. However, no such ultrastructure or any subassembly thereof has been isolated biochemically: Capping enzyme can form a binary complex with RPO *in vitro* [24], but the soluble fraction from a sarkosyl virion core lysate, for example, even under gentle gradient sedimentation conditions, has yielded no higher order assemblies beyond the sedimentation of RPO as a discrete entity and the partial co-sedimentation of RPO with viral capping enzyme and NTP1 [25]. Other enzymes, including those apparently co-packaged with RAP94 (above) sedimented separately, towards the top of the gradient, suggesting an irreversible disruption of interactions within the transcriptosome upon core rupture. To our knowledge, no comprehensive transcriptosome, or other packaged superstructure has been (re)assembled biochemically as a positive correlate to the subtractive approaches of genetics.

Here, we have taken an approach to the molecular structure of the Vaccinia virion that is neither destructive, reconstructive nor exclusively applicable to binary complexes, namely protein-protein crosslinking mass spectrometry (XL-MS). We address the virion in its natural state *in situ*, with the potential to interrogate multivalent protein complexes. Technical challenges in this approach were not inconsiderable: At the outset of the current study, higher profile XL-MS studies in the literature had focused upon stoichiometric or near-stoichiometric isolated protein complexes, containing around ten or fewer polypeptides, with known crystal structures. Examples of these would include the 26S proteasome [26], multi-ringed TRiC/CCT chaperonin [27, 28], the RNA polymerase II pre-initiation complex [29–31], RNA polymerase I [32] and RNA polymerase III [33]. By contrast, the Vaccinia virion likely contains a variety of protein complexes covering an abundance dynamic range of ~5000 [34] or greater, only a minority of which have yielded X-ray crystallographic structures. Our XL-MS results with Vaccinia are described below.

## Results

### Approach

Virions (intact or activated for mRNA transcription) were incubated with bifunctional chemical crosslinkers to impose inter-protein distance restraints. Crosslinked virus was then dissolved and trypsinized to peptides, followed by peptide-level nanoLC-MS/MS and bioinformatics to identify crosslinked peptides. For disuccinimidyl suberate (DSS), the crosslinker used in the majority of experiments, the restraint comprised a lysine Nζ-Nζ distance of ≤ 10–11.4 Å with corresponding Cα-Cα distances of ≤ 32 Å (give or take molecular dynamics considerations). Crosslinkable lysines thereby sweep a sphere of Cα-Cα distances up to ~6 nm, or ~2% of the diameter of a Vaccinia virion for proteins not forming extended, repeating arrays.

**Fig 1:**
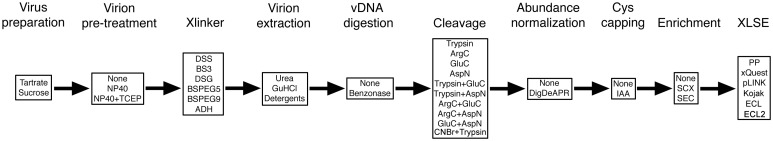
Vaccinia MV protein crosslinking multi-threaded workflow and strategy of variation. In total, 49 distinct pathways through the conditions matrix (Table 1) were sampled over 53 experiments. The final step of the workflow (‘XLSE’, for ‘crosslink search engine’) was a parallel, rather than a variable element.

Due to the low intrinsic ionizability of crosslinked peptide pairs and the potential for low saturation crosslinking within/between low abundance proteins in the virion, a strategy of variation [5] (Table 1) was implemented to maximize opportunities for the detection of crosslink (XL) ions (Fig 1). This was combined with a total of six distinct XL search engines, used in parallel (Fig 1 and Materials & methods). After data thresholding and filtering, a unique meta-score (‘DFscore’, or detection frequency score) was introduced as a guide to the extent of internal confirmation within the dataset.

**Table 1:** Crosslinking experiments and experimental conditions. 49 distinct sets of experimental conditions were sampled as a sparse-matrix through Fig 1.

Condition#	Pre-XL		Xlinker	Post-XL		Digestion			Enrichment	XLSE					
	Virus prep	pre-treatment		Extraction	DNA digestion	Cleavage	Normalization	Iodoacetamide		PP	xQuest	pLINK	Kojak	ECL	ECL2
1	Sucrose	N	DSS	Urea	-	Trypsin	-	Y	-	Y	Y	Y	Y	Y	Y
2	Sucrose	NT	DSS	Urea	-	Trypsin	-	Y	-	Y	Y	Y	Y	Y	Y
3	Sucrose	None	DSS	Urea	-	Trypsin	-	Y	-	Y	Y	Y	Y	Y	Y
2	Sucrose	NT	DSS	Urea	-	Trypsin	-	Y	-	Y	Y	Y	Y	Y	Y
4	Sucrose	None	DSS	Urea	-	Trypsin	-	N	-	Y	Y	Y	Y	Y	Y
5	Sucrose	N	DSS	Urea	-	Trypsin	-	N	-	Y	Y	Y	Y	Y	Y
6	Sucrose	NT	DSS	Urea	-	Trypsin	-	N	-	Y	Y	Y	Y	Y	Y
7	Sucrose	NT	DSS	GuHCl	-	Trypsin	-	N	-	Y	Y	Y	Y	Y	Y
8	Tartrate	NT	DSS	Urea	Benzonase	Trypsin	-	Y	-	Y	Y	Y	Y	Y	Y
9	Tartrate	NT	DSS	Urea	Benzonase	AspN	-	Y	-	Y	Y	Y	Y		
10	Tartrate	NT	DSS	Urea	Benzonase	ArgC	-	Y	-	Y	Y	Y	Y		
11	Tartrate	NT	DSS	Urea	Benzonase	GluC	-	Y	-	Y	Y	Y	Y		
12	Tartrate	NT	DSS	Urea	Benzonase	Trypsin	-	Y	SCX	Y	Y	Y	Y	Y	Y
13	Tartrate	NT	BS3	Urea	Benzonase	Trypsin	-	Y	-	Y		Y	Y		
14	Tartrate	NT	DSS	Urea	Benzonase	Trypsin	DigDeAPR	Y	-	Y	Y	Y	Y	Y	Y
15	Tartrate	NT	DSS	Urea	Benzonase	AspN (new)	-	Y	-	Y		Y	Y		
16	Tartrate	NT	DSS	Urea	-	Trypsin	-	Y	-	Y	Y	Y	Y	Y	Y
17	Tartrate	NT	DSS	Urea	-	Trypsin+AspN	-	Y	-	Y		Y	Y		
18	Tartrate	NT	ADH	Urea	-	Trypsin	-	Y	-	Y		Y	Y		
19	Tartrate	NT	DSS	Urea	-	Trypsin	-	Y	SEC	Y		Y	Y		
20	Tartrate	NT	DSG	Urea	-	Trypsin	-	Y	-	Y		Y	Y		
21	Tartrate	NT	BSPEG9	Urea	-	Trypsin	-	Y	-	Y		Y	Y		
22	Tartrate	NT	BSPEG5	Urea	-	Trypsin	-	Y	-	Y		Y	Y		
23	Tartrate	NT	DSS	Urea	-	Trypsin+GluC	-	Y	-	Y		Y	Y		
24	Tartrate	N	ADH	Urea	-	Trypsin	-	Y	-	Y		Y	Y		
25	Tartrate	N	DSS	Urea	-	AspN+GluC	-	Y	-	Y		Y	Y		
26	Tartrate	N	DSS	Detergents	-	AspN+GluC	-	Y	-	Y		Y	Y		
27	Tartrate	N	DSS	Urea	-	ArgC+GluC	-	Y	-	Y		Y	Y		
28	Tartrate	N	DSS	Urea	-	ArgC+AspN	-	Y	-	Y		Y	Y		
29	Tartrate	N	DSS	Urea	-	AspN+GluC	-	Y	SCX	Y	Y	Y	Y		
30	Tartrate	N	DSS	Urea	-	Trypsin	DigDeAPR	Y	-	Y	Y	Y	Y	Y	Y
31	Tartrate	N	DSS	Urea	-	Trypsin+GluC	DigDeAPR	Y	-	Y		Y	Y		
32	Tartrate	N	DSS	Urea	-	Trypsin	DigDeAPR	Y	SCX	Y	Y	Y	Y	Y	Y
33	Tartrate	N	ADH	Urea	-	Trypsin	-	Y	-	Y		Y	Y		
34	Tartrate	N	DSS	Detergents	-	Trypsin	-	Y	-	Y		Y	Y	Y	Y
35	Tartrate	NT	DSS	Urea	-	LysN	-	Y	-	Y		Y	Y		
36	Tartrate	NT	DSS	Urea	-	LysC	-	Y	-	Y		Y	Y		
37	Tartrate	N	DSS	Urea	-	Trypsin+GluC	-	Y	SCX	Y	Y	Y	Y		
38	Tartrate	N	DSS	Urea	-	LysN	-	Y	-	Y		Y	Y		
39	Tartrate	N	ADH, EDC	Urea	-	Trypsin	-	Y	-	Y		Y	Y		
40	Tartrate	N	ADH, EDC/NHS	Urea	-	Trypsin	-	Y	-	Y		Y	Y		
41	Tartrate	N	DSS	Urea	-	Trypsin+GluC	DigDeAPR	Y	SCX	Y	Y	Y	Y		
42	Tartrate	N	DSS	Urea	-	Trypsin+AspN	DigDeAPR	Y	SCX	Y	Y	Y	Y		
43	Tartrate	N	DSS	Urea	-	AspN+GluC	DigDeAPR	Y	SCX	Y		Y	Y		
44	Tartrate	N	DSS	Urea	-	LysN+GluC	DigDeAPR	Y	SCX	Y		Y	Y		
45	Tartrate	N	DSS	Urea	-	LysN+AspN	DigDeAPR	Y	SCX	Y		Y	Y		
46	Tartrate	N	EDC	Urea	-	Trypsin+AspN	DigDeAPR	Y	SCX	Y		Y	Y		
47	Tartrate	N	EDC	Urea	-	Trypsin+GluC	DigDeAPR	Y	SCX	Y		Y	Y		
48	Tartrate	N	DSS	70% FA	-	CNBr-Trypsin	-	N	-	Y		Y	Y		
49	Tartrate	None	DSS	Urea	-	Trypsin	DigDeAPR	Y	SCX	Y	Y	Y	Y	Y	Y

### Overall project dataset (‘crosslinkome’)

The resulting XL dataset yielded a total of 4609 confidently-identified unique-mass ions, each corresponding to a crosslinked peptide pair (S1A and S1B Table). Of these, 1486 (32.2%) had a DFscore > 1. The highest DFscore for any ion was 178, and the four top-scoring ions each corresponded to p4a intra-protein XL, of which the two highest scoring were light/heavy versions of the same ion and the third represented a small shift in XL position for one of the two crosslinked peptides (S1A and S1B Table)). 3725 of the 4609 unique-mass ions represented intra-protein XL while 884 were inter-protein, consistent with the known tendency for XL to fall within rather than between proteins. 273 of the 884 inter-protein XL ions had a DFscore > 1 among which the highest DFscore was 83 (p4a-position 876 crosslinked to p4b-position 563).

By merging (a) distinct charge states for a crosslinked peptide, (b) identical crosslinked accessions/positions detected within distinct peptide species, (c) light/heavy isotopic forms of the crosslinker and (d) crosslinked peptides with secondary modifications, the 4609 unique XL ion masses collapsed down to 2534 unique pairs of residues within the proteome. 625 of these were inter-protein and, of these, 157 (25.1%) had a DFscore > 1 with the highest DFscore for an inter-protein accession/position pair being 475 (for the p4a-876/p4b-563 XL mentioned above). This accession/position pair was represented by 43 distinct m/z crosslinked peptide ions. S1C Table shows all crosslinked protein pairs in the dataset. S1 Fig shows crosslinking partners among all proteins considered to be packaged in the virion [5] for which XL were detected, and Table A in S1 Text reconciles the proteome of S1 Fig with the contents of the XL search database.

### Validation

Orthogonal approaches to the validation of *in situ*–detected protein-protein interactions all seemed less direct than XL-MS itself (involving virion disruption, recapitulation of interactions *in vitro*, and/or the expression of virus proteins in heterologous systems). We therefore sought to validate the XL dataset via inference criteria, asking four basic questions as follows:

### (a) Was reasonable bioinformatic rigor applied (eg. in program score thresholding)?

All six XL search engines employed a target-decoy approach [35] (Table 2) and primary score thresholding comprised false discovery rate (FDR) or its surrogate, q-value (Materials & methods). For four of the six engines we took the unprecedented step of also applying a second threshold, via the score-type that is native to the engine itself (Table 2). A small fraction of the ions discarded solely on the basis of threshold 2 were then rescued according to the criteria described in Materials & Methods. With a primary threshold alone, namely 5% FDR, around 230 of our 4609 unique-mass ions would have arisen from our decoy database. Via our dual thresholding/rescue approach (see the “Data Assembly” section of “Materials & methods”), only 15 of the 4609 ions involved a decoy accession, representing an effective FDR of just 0.33%—an exceptionally low number. We regard our low effective FDR as a bona fide validation step, and an indication of low technical noise in the dataset. All 15 decoy hits had a DFscore of 1 with one exception, whose DFscore was 2.

**Table 2:** XL search engine score thresholds. Second thresholds are native to individual search engines. SD-E is described in Materials & Methods, PEP = posterior error probability.

Program	Inbuilt threshold	Primary threshold	Second threshold
Protein Prospector	-	FDR = 6%	SD-E (≥ 5)
pLINK	FDR = 5%	-	e-value (≤ 0.1)
xQuest -> xProphet	-	FDR = 6%	ID-Score (≥ 20)
Kojak -> Percolator	-	q-value ≤ 0.01	PEP (≤ 0.9)
ECL/ECL2	-	q-value ≤ 0.01	-

### (b) Did data appear statistically non-random?

Non-randomness was evaluated on the basis of several criteria:

Inter-protein vs. intra-protein XL: For a database of 86 proteins, random partner selection would result in a 1/86 (1.12%) chance of both tryptic peptides in a crosslinked pair arising from the same protein, assuming an equal number of tryptic peptides from each protein in the database. Experimentally, however, far more opportunities exist for efficient crosslinking within a protein than between proteins. Of the 1742 unique accession/position pairs in the dataset, 1294 (74.3%) were intra-protein, conforming to the experimental expectation rather than the random selection of peptides during bioinformatics.

Protein abundance: During MS data acquisition, ions were prioritized for sequencing on the basis of intensity (high-to-low) leading to an expectation of XL detection at a higher frequency for relatively abundant proteins. Consistent with this, the dataset was dominated by XL between the abundant virion structural proteins p4a and p4b (S1C Table). This provided a clear validation of data on the basis of known protein abundance.

**Fig 2:**
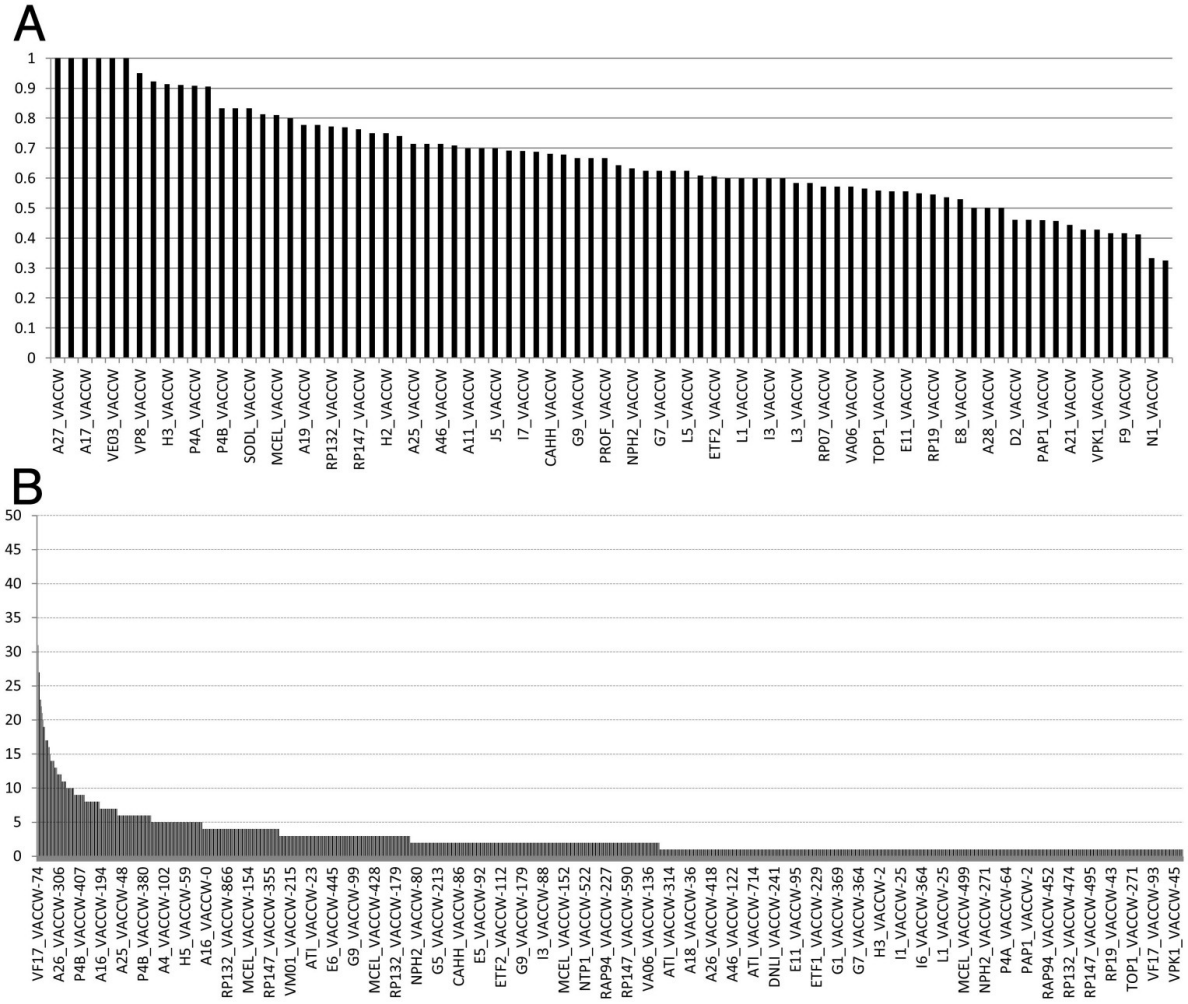
(**a**) Bar chart showing the extent of lysine occupancy with XL, per protein. X axis: All proteins within which lysine XL were detected (every alternate accession is named). Y axis: Proportion of the protein’s lysines found to be crosslinked in the dataset, which ranged from 32.5% to 100%. (**b**) Bar chart showing the popularity of each unique pair of crosslinking site in the dataset. X axis: Individual lysines in the dataset (there are 1742 bars in total, with only every ~31th bar labeled). Y axis: Number of unique crosslinking sites to which it was attached, ranging from 45 (left) to 1 (right).

Non-random lysine occupancy per protein: If search engines were picking lysine XL sites randomly, then the proportion of lysines occupied with XL would be expected to be fairly constant from protein-to-protein. However, lysine occupancy on a per protein basis covered a broad range, from 32.5% to 100% (Fig 2a). Search engines were therefore not simply picking sites from the database randomly. Some proteins were clearly more ‘detectably crosslinkable’ than others for reasons that presumably included protein abundance, solvent accessibility and lysine basicity for reaction with succinimide-based crosslinkers.

Non-random ‘hotspotting’ of lysine XL sites within a protein: Individual XL sites within a protein may vary in exposure, reactivity or flexibility or the number of reactive partners within crosslinking range, resulting in the appearance of crosslinking ‘hotspots’ [36]. The crosslinkability of some protein N-termini in particular (S1 Fig) likely arises from their exposure and flexibility, combined with a pKa [37] that promotes chemical reactivity. Consistent with this, individual lysines in our dataset showed substantial variation in predisposition towards XL ‘hotspotting’ (Fig 2b). F17 residue K74, for example, provided a particularly concentrated crosslinking hotspot, appearing in a total of 45 distinct accession/position pairs (Fig 2b) among 15 protein partners (S1 Fig). By contrast, many other positions in various accessions appeared just once (Fig 2b, S1 Fig).

Non-random coverage of inter-protein XL space: Our 86-protein search database provided a theoretical space of 3655 potential protein-protein pairs from which the XL dataset contained just 449. Despite the depth of analysis (4609 XL ions), this 12.3% coverage of theoretical inter-protein crosslinking space suggested a level of specificity.

### (c) Did data appear structurally rational, using PDB co-ordinates for known virion protein structures?

**Fig 3:**
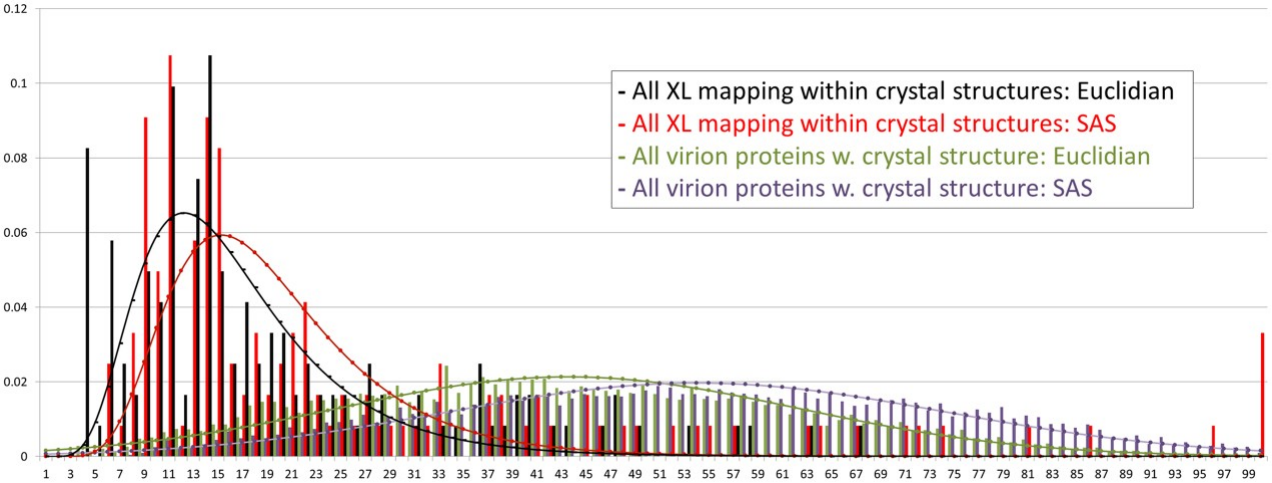
Histograms (bars), and fits (lines) to the histograms, showing all lysine-lysine Cα –Cα Euclidian (green) and SAS (purple) distances from within all virion proteins whose crystal structures have been reported to date (Table B in S1 Text; overlay = normal distribution). Superimposed are Euclidian (black) and SAS (red) distances between those crosslinked residues in our dataset that mapped within these structures (overlay = log-normal distribution). X: Distance (Å). Y: Proportion of total (ie. of all values summed for a histogram or distribution) found within an individual bar or point. XL lengths < 32 Ǻ were considered structurally rational for crosslinkers DSS/BS3. For the longer crosslinkers BSPEG5/9, no XL exceeded an SAS distance of 30 Å. For zero-length XL (EDC), Cα –Cα SAS distances for crosslinked residues of < 18.6 Å were considered structurally rational. The few violators of these restraints likely represent inter-subunit XL within homomultimers as opposed to intra-protein XL. Distances for Vaccinia Profilin were based on a homology model of the Monkeypox ortholog (PROF_MONPZ, Table B in S1 Text).

At the time of writing, partial or complete X-ray crystallographic structures covered the crosslinked portions of 12 proteins in our XL dataset, with an additional two crystallographic structures from other orthopoxviruses (Table B in S1 Text). All possible lysine-lysine through-space (Euclidian) and solvent-accessible surface (SAS) distances within all of these structures [38] were binned, and the resulting two histograms were found to be centered at ~43 and ~54 Å, respectively (Fig 3). By contrast, the Euclidian/SAS distance histograms for all experimental XL found within the 14 proteins was centered at 14.9 and 13.5 Å respectively, with 103 or 114 (SAS/Euclidian) out of the 136 experimental XL distances being structurally rational (≤ 32 Å, Cα to Cα distance). Based on the Kolmogorov-Smirnov test, the probability that the “All lys-lys” and “experimental XL” distance histograms (Fig 3) were sampled from a single population was < 10^−4^, providing 99.99% statistical confidence that the crosslinking dataset was structurally rational.

### (d) Were data biologically rational, regarding known protein functions and the biology of the virus?

**Fig 4:**
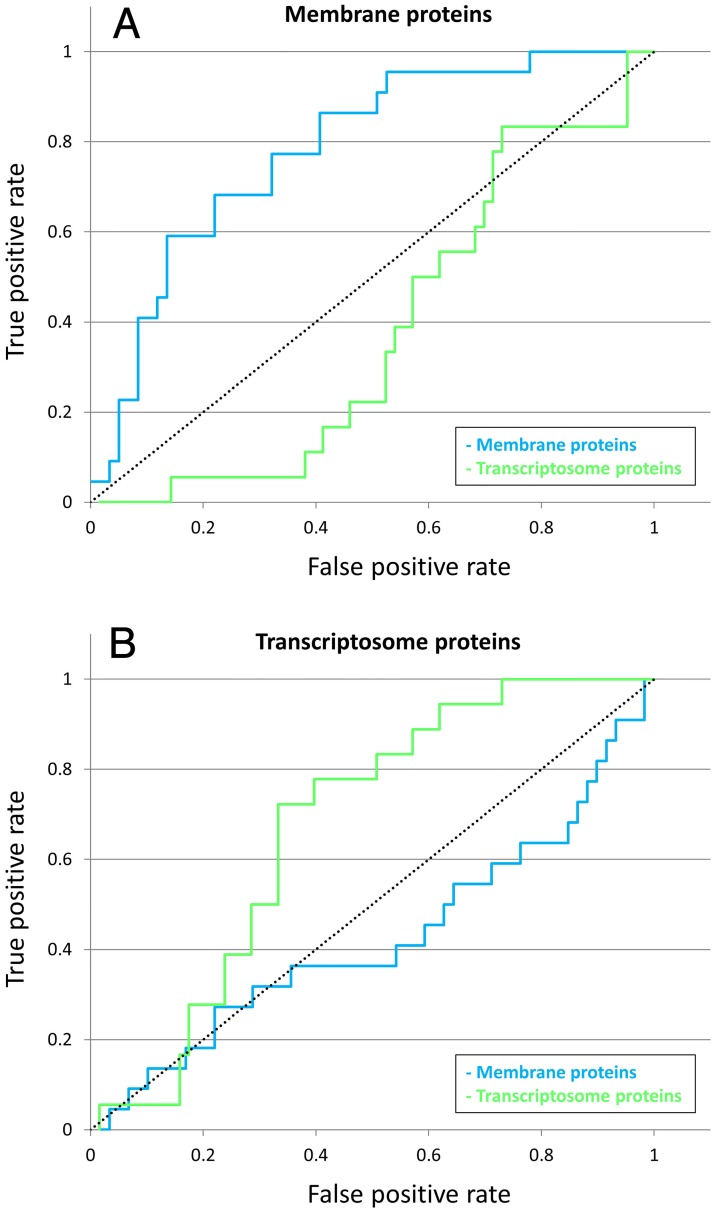
‘Biological rationality’ in the dataset, as ROC (‘receiver operating characteristic’) curves. Proteins with strong functional annotations were divided into functional groups (Table 3) and on this basis were subjected to ROC analysis (Materials & methods). Briefly, a listing of all virion proteins with crosslinking partners was ranked by proportion of partners that were classed as: (**a**) membrane, (**b**) transcriptosome. ROC curves score, proportionately (0 to 1), positions (y) vs. not(positions) (x) in the ranking that correspond to membrane proteins (blue) or transcriptosome proteins (green). The line of no-discrimination (neutrality) is shown black, dotted. A colored line curving above the diagonal indicates a positive correlation, and vice versa.

Further assessment of the XL dataset was largely biological, namely, whether the identities of crosslinked protein pairs were consistent with known protein functions. For this analysis, accessions with strong functional annotations were collected into groups (Table 3). Interactions within any group were considered ‘biologically rational’, while the pairing of a membrane-group protein with a transcriptosome-group protein was designated ‘biologically non-rational’ since these two groups of proteins are considered, based on controlled degradation studies [9, 16], the most likely among the various groups to occupy distinct virion compartments—separated by the core wall. All other protein-protein pairings were disregarded for the purposes of biological validation as being relatively uninterpretable. Membrane-group proteins showed a moderate, yet unmistakable global positive predilection for other membrane-group proteins as crosslinking partners, and a mild antipathy, globally, for transcriptosome proteins (Fig 4a). Transcriptosome proteins, as a class, showed a mild but unmistakable predilection for other transcriptosome proteins as crosslinking partners and a mild antipathy for the membrane class (Fig 4b). While not absolute, the trends shown in Fig 4 were consistent with accepted compartmentalization models for virion proteins, with the likely location of the transcriptosome within the virion core enclosed by a core wall, and virion TM proteins likely occupying a two-dimensional membrane compartment surrounding the core wall. This provided a suggestion of biological rationality within the XL dataset. Among the top 28 crosslinked protein pairs by DFscore, 12 were ‘rational’ and only 2 were ‘non rational’ (S1C Table). The top 28 protein pairs contained 1205 of the 1849 total XL ions represented in S1C Table, and the top 12 “Y” protein pairs represent 92% of all XL ions in S1C Table associated with a “Y” (ie. that were biologically ‘rational’).

**Table 3:** Six functional groups covering 68 Vaccinia virion accessions: ‘7PC’ (seven protein complex), DNA, ‘membrane’ (MV transmembrane and membrane-associated proteins),’ structural’, ‘thiol’ (redox plus an additional glutaredoxin), transcriptosome (mRNA biogenesis). ‘#mem’ = number of members in each group, ‘#comb’ = number of pairwise combinations within a group according to n!/k!(n-k)! (subset of k distinct elements from an n-element set). There are 450 theoretical pairs of ‘membrane’ with ‘transcriptosome’-group proteins (these pairs being designated ‘non-rational’). Some accessions were reassigned during the study (eg. VP8 away from ‘DNA’). The ‘Membrane’ group was chosen to represent all MV proteins with detectable transmembrane domains plus MV proteins considered to be membrane-associated (A26, A27). Since WV-specific proteins were not considered in the current study, VENV (F13L), a membrane-associated WV-specific protein, was included in the search DB in error. A11 (a ‘VMAP’ [39]) is also considered to be not packaged in Vaccinia MV [2]. No XL at all for A14 and I2 were detected in this study. The Vaccinia stub of the cowpox ATI is considered, here, to be a membrane protein. VP8, a virion core protein, is included in the ‘DNA’ group due to its nucleic acid binding properties [40] as opposed to a known role in conjunction with the Vaccinia genome.

Group	Proteins	#mem	#comb
7PC	A30,G7,J1,A15,D2,D3,F10	7	21
DNA	DNLI,I1,I3,K4,G5,H5,I6,TOP1,VP8	9	36
Membrane	A9,A11,A13,A14,A16,A17,A21,A28,ATI,CAHH,E8,F9,F14.5,G3,G9,H2,H3,I2,I5,J5,L1,L5,O3,A26,A27,VENV	26	325
Structural	A4,P4A,P4B	3	3
Thiol REDOX	A2.5,E10,GLRX1,GLRX2	5	10
Transcriptosome	MCE,MCES,MCEL,NPH2,NTP1,PAP1,RAP94,RP07,RP18,RP19,RP22,RP30,RP35,RP132,RP147,L3,ETF1,ETF2	18	163
	**TOTAL**:	**68**	**558**

### Vaccinia virion crosslinkome

#### Virion structural proteins

**Fig 5:**
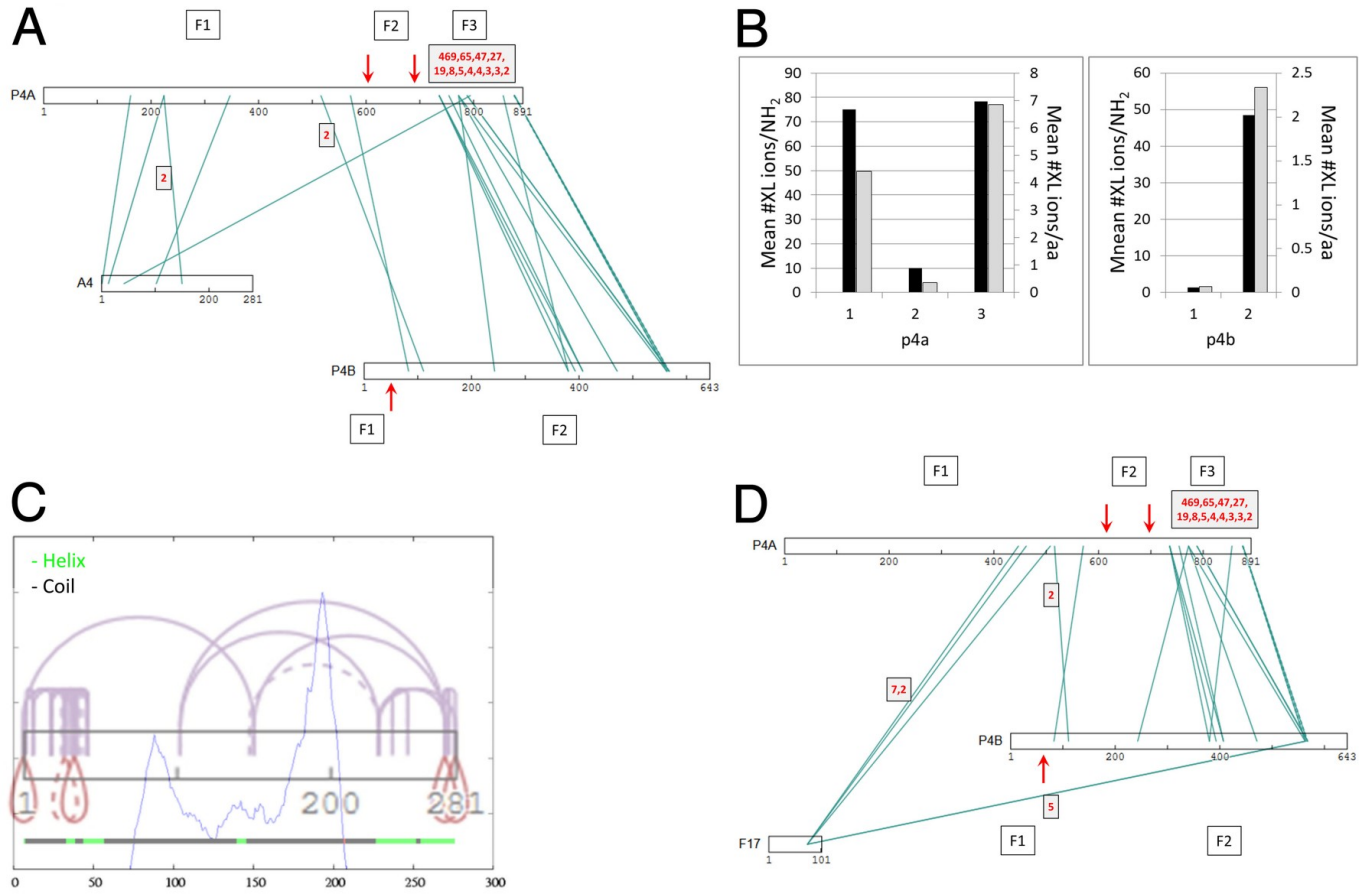
(**a**) XL interactions between the three major structural proteins p4a, p4b and A4 (p39). F1, F2, F3 (boxed): fragments 1, 2 and 3 of p4a, and fragments 1 and 2 of p4b generated during virion maturation by AG|-specific processing after p4a residues 614 and 697 [139] and p4b residue 61 [140] (red vertical arrows). (**b**) Mean # of XL ions (intra- and inter-molecular) detected per amino group (lysine sidechain and fragment N-terminus; Y1 axis, black bars) and per residue (Y2 axis, gray bars) in fragments 1, 2 and 3 of p4a (left), and fragments 1 and 2 of p4b (right), as generated by AG|-specific processing at the sites indicated in panel A. Raw numbers are given in Table C in S1 Text. (**c**) Predicted three-domain structure for protein A4. Lower horizontal line: Predicted helix (green) and coil (black). Blue trace: Predicted domain boundaries at residues 88 and 193 based on an endpoint density profile from 2718 PSIBLAST hits. Mauve: Intra-A4 XL. The density of XL within N- and C-terminal regions is consistent with the presence of discrete N- and C-terminal domains coincident with the green helical regions. XL from both protein termini to positions 100 and 150 (towards the N- and C-terminal ends, respectively of the short central domain located between residues 88 and 193) suggest that the central region of A4 is within crosslinking range of the two terminal domains. (**d**) XL interactions of structural proteins p4a and p4b with protein F17. Vertical red arrows: As in panel A. In panels A, D: Numbers (red font) in gray squares with black border: DFscore (XL with no red-font numbers had a DF score of 1).

Three major structural proteins of the virion, p4a (A10), p4b (A3) and A4 (p39), are thought to comprise the wall of the virion core ([2] and references therein). Among the most clearly discernible interactions in the XL dataset was a connection between the C-terminal portions of p4a and p4b (Fig 5a) providing, perhaps, the first structural information on the mutual arrangement of p4a and p4b in MV. Among the three fragments of p4a arising from proteolytic processing during MV maturation, p4b was most abundantly crosslinked to fragment 3 (the C-terminal proteolytic product), with an additional pair of XL connecting p4b’s N-terminal region (around residue 100) to the C-terminal end of p4a fragment 1 (Fig 5a). In this manner, p4b may bring p4a fragments 1 and 3 together after p4a cleavage. The fate of p4a fragment 2 is unknown: The density of XL ions in this fragment was dramatically lower than in fragments 1 or 3 (Fig 5b, Table C in S1 Text) suggesting that fragment 2 is discarded or degraded after its excision during virion maturation, with the few residual detectable XL in fragment 2 perhaps representing low level contamination of MV preparations with pre-cleavage viroforms. A similar suppression of XL density was apparent for the N-terminal cleavage product of p4b (Fig 5b, Table C in S1 Text). This correlation of XL density with known fragments of p4a and p4b provided additional validation for the dataset as a whole.

Protein A4 interacted with p4a but not p4b (Fig 5a), consistent with prior immunoprecipitation and immunogold EM co-localization studies showing a stable interaction between p4a and A4 [41]. p4a-A4 XL were, with one exception, between the N-terminal ~half of A4 and residues 170–350 of p4a fragment 1. In immunoEM studies, antibodies to A4 decorate a region of MV between the core and outer envelope [2] or stain the surface of the exposed virion core [9], and A4 has been suggested to reside in a ‘spike’ or ‘palisade’ layer on the exterior of the core wall [7, 8]. Fig 5c shows a predicted three-domain structure for A4. Short-range intra-protein XL tended to cluster within the predicted N- and C-terminal domains with these two domains donating longer range XL to a third, central domain. The majority of inter-protein XL to A4 were within its N-terminal half (S1 Fig), the only exception being the strongly-detected XL between A4’s C-terminal region and protein F17 (below).

**Fig 6:**
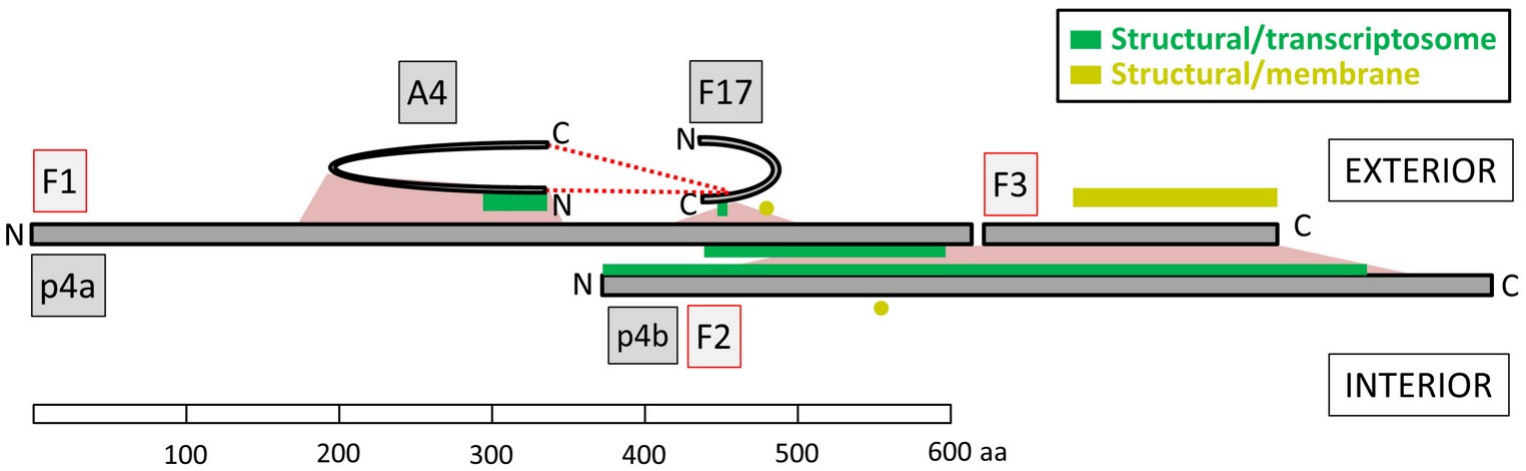
Simplest arrangement satisfying topological restraints between structural proteins p4a, p4b, A4, and F17. ‘N’, ‘C’, ‘F1’, ‘F3’, ‘F2’ refer, respectively, to protein N- and C-termini, proteolytic fragments 1 and 3 of p4a and fragment 2 of p4b. p4a-fragment 2 and p4b-fragment 1 have been discarded. Pink trapezoids: The p4a/p4b and p4a/A4 interaction regions (from Fig 5a). Pink wedge: The F17(K74)/p4a interaction region (from Fig 5d). Broken red lines: XL from F17(K74) to both ends of protein A4 (from S1 Fig). The arrangement shown also emphasizes the absence of p4b interaction with either A4 or F17 and the absence of any interactions within the four-protein complex of protein A4’s C-terminal half or p4a’s N-terminal region (Fig 5a and 5d). Also accounted for are the three-domain structure of protein A4 (Fig 5c) and a core-wall exterior location for A4 as predicted by immunoEM studies ([7, 8]). Among the membrane proteins, interaction sites for J5, A21, H3, A16, G3, A26 and ATI cluster at the C-terminal region of p4a (yellow line) while A17 interacts further upstream, with p4a fragment 1 (yellow circle; from S1 Fig). p4b’s only interaction with a bona fide membrane protein is with A17 (yellow circle; from S1 Fig). This membrane protein arrangement suggests that p4b may be oriented towards the interior of the core. Green lines: Regions of structural proteins that interact with transcriptosome components (from S1 Fig). These presumably extend into the third dimension.

The short (101 amino acid) virion protein F17 formed multiple XL to a localized region of p4a fragment 1 between residues 450 and 510, just upstream of the fragment 1 interaction site of p4b (Fig 5d). All p4a XL to F17 were via a single lysine ‘hotspot’ of F17, namely K74, the most intensively focused XL hotspot of any found in the current study (see above). K74 may therefore form an anchor point for a number of virion proteins. F17 also formed a very strongly-detected (DFscore = 11) interaction with the C-terminus of A4 (S1 Fig) as well as A4’s N-terminal region (DFscore = 3). Due to its strong association with p4a and A4, combined with its high abundance in the virion, F17 is considered a good candidate to be a major structural protein of the core wall. F17 has been reported, by immunogold EM, to be a lateral body protein [10], an observation neither inconsistent nor mutually exclusive with its snug fit to the exterior of the core wall exterior shown here. Fig 6 shows a topological arrangement for proteins p4a, p4b, A4 and F17 that satisfies the deduced restraints.

#### The pairing of virion core proteins VP8 and A12

**Fig 7:**
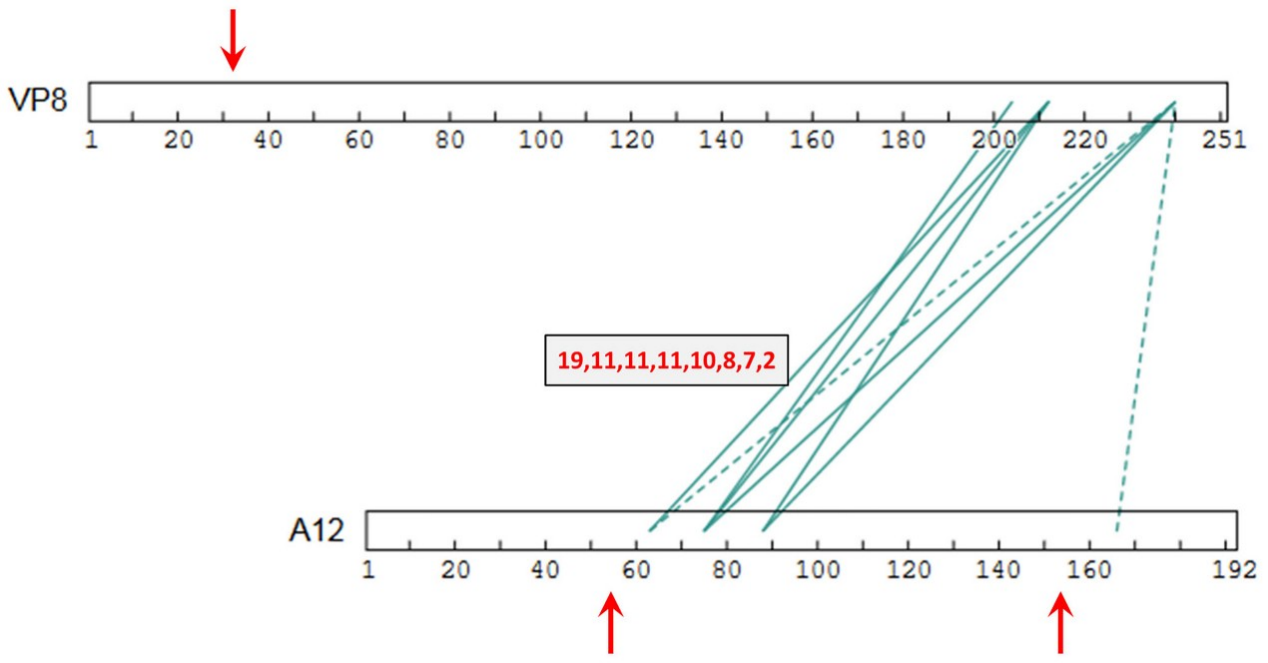
(a) XL interactions between proteins VP8 and A12, showing AG|-specific processing sites (vertical red arrows). Details as in Fig 5.

Another strongly-detected protein pairing was the 251 residue protein VP8 and the 192 residue protein A12, crosslinked via their final ~50 residues and residues 63–88, respectively (Fig 7). The association of these two proteins seems to be a new finding. VP8, a virion core protein with nucleic acid binding properties *in vitro* [40], is required for the production of infectious, morphologically and transcriptionally normal MV [42, 43]. It is exposed in core material only under the harshest conditions, such as during the use of a virus mutant in p4b along with DNase [9]. A12, a core protein of unknown function, is also essential for the formation of a structurally normal core [2]. Mutants in both VP8 and A12 show morphological defects in IV membrane adhesion to viroplasm during virion morphogenesis [42, 44] and, during the morphogenic transition from IV/IVN (immature virus; immature virus with nucleoid) to MV, both proteins are N-terminally processed [2, 45] via AG| -specific cleavage immediately after residues 32 and 56, respectively [46] [45, 47]. The A12 precursor may be only incompletely processed at this site [44]. A12 seems to be partially C-terminally processed also, after residue 154, with the C-terminal fragment detectable [47].

A12 showed two emphatically detected crosslinking hotspots, centered at residues 88 and 167 (S1 Fig), located within fragments 2 and 3, respectively, of the three-fragment protein if doubly-processed. The two hotspots and the protein N-terminus were strongly connected to one another via intra-protein crosslinking (S1 Fig) suggesting that the processed fragments of A12 remain together after proteolytic processing. The strongly detected interaction of A12’s N-terminus with protein H3 (DFscore = 6, S1 Fig), a virion-resident TM protein, also suggested that the N-terminal fragment is not discarded after A12 cleavage. A12 showed six transcriptosome partners, all crosslinked at the residue 167 hotspot (S1 Fig). We speculate that A12 may span the core wall with fragment 3 contacting transcriptosome and p4b interiorly, and fragments 1 and 2 oriented towards the exterior contacting A4, p4a, F17 and TM proteins H3, F9 and H2 (S1 Fig). The compromised VP8 encapsidation upon repression of core wall protein p4b [48] seems consistent with a core wall connection to VP8 also, as does the compromised core wall in absence of VP8 [49]. Since, minimally, ~57 amino acids (aa) of linear beta sheet would be required cross a 20 nm core wall, contacts within A12 of the C-terminal 20% of VP8 and some membrane proteins, appear to be more intimate than a wall’s-width. We suppose that this apparent intermingling of the VP8 C-terminal region with membrane proteins as well as A12 could be outside, within, or immediately interior to the wall.

A12 also showed connectivity with G1 metalloprotease (S1 Fig) but not the I7 cysteine protease, the implications of which are unclear. The finding of protein A19 as a crosslinking partner for A12 (at the central hotspot of A12, S1 Fig) was consistent with the A12-A19 interaction detected by yeast two-hybrid (Y2H) analysis [20]. Among VP8’s 12 crosslinking partners (S1 Fig) was the short (65 aa) F8 protein, of unknown function, supporting the finding of an association of VP8(L4) with F8 by Y2H analysis [20]. F8’s XL fell close to the VP8 C-terminus.

#### A17-H3-A27-A26 membrane protein network

**Fig 8:**
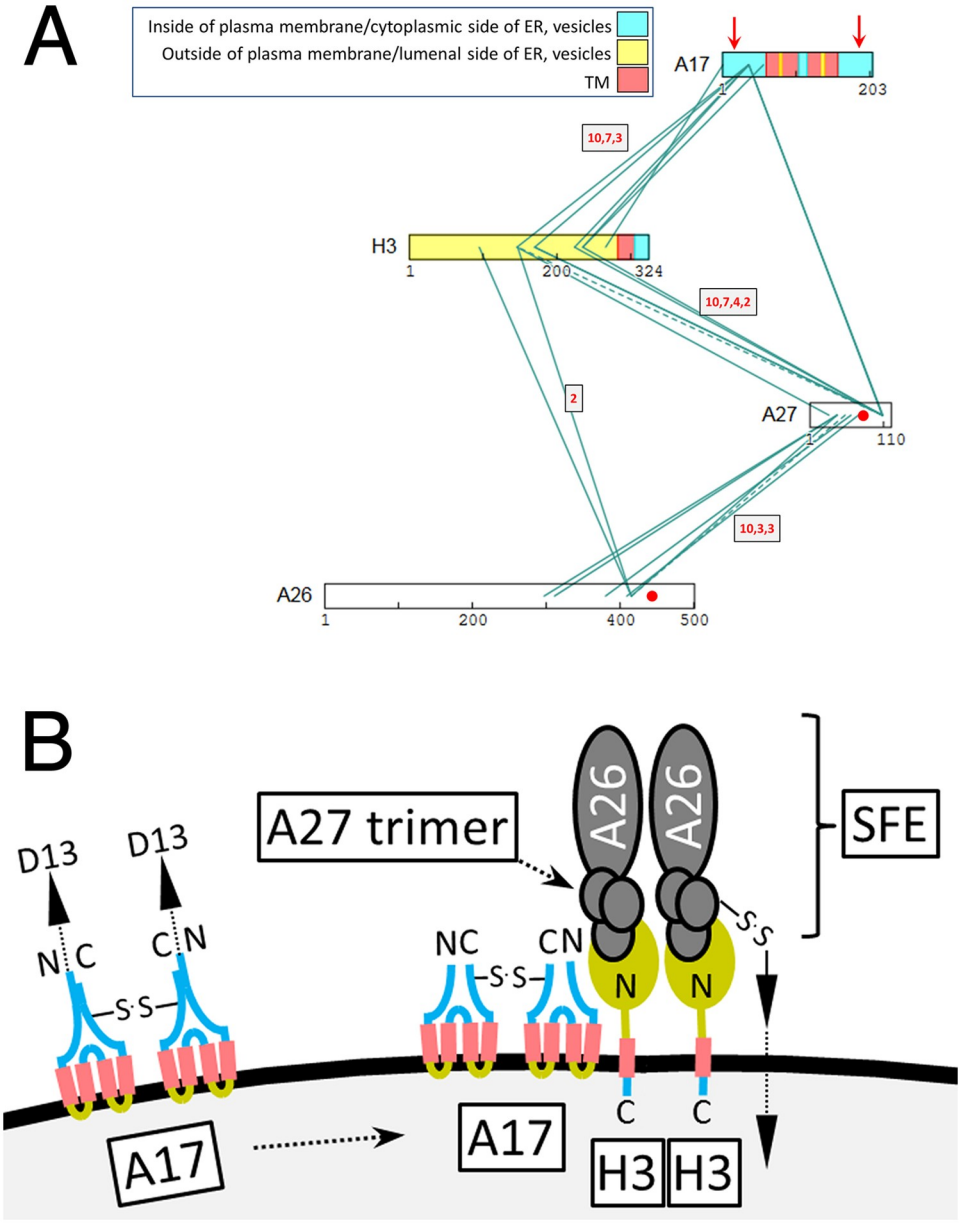
(**a**) Crosslinkome for major membrane proteins A17, H3, A27, A26. Yellow, red and cyan fill: ‘Outside’, TM and ‘inside’ domains, respectively, with “Inside” and “Outside” following the convention of the program TMHMM [68] in which “inside” refers to the cytoplasmic side of plasma membrane, ER membrane or vesicles for a classically embedded TM protein, and “outside” refers to the lumenal side or the ER or external side of the plasma membrane. Vertical red arrows: AG|-specific processing sites in protein A17. Red spots (A26, A27): Cysteines that are disulfide bonded to one another. Other details as in Fig 5. (**b**) Arrangement, at the MV envelope, of proteins shown in panel A satisfying XL, functional and imaging data. Left side: Disulfide bonded homodimers of unprocessed A17 in the IV envelope in reticulon conformation. The unprocessed A17 N-terminus is within crosslinking range of A17’s C-terminal region. The D13 external scaffold is not depicted. Right side: Maturation of the envelope includes A17 N- and C-terminal cleavage and D13 loss. H3 is now non-canonically tail-anchored in the MV envelope with immunodominant N-terminal domain exposed, and SFEs have been added as stacked trimers of A27 and associated A26. H3’s external N-terminal domain mediates most of the interaction between A27 and the processed A17 N-terminal region, with the A17 C-terminus now out of range of H3. The H3/A26 and A27/A17 protein pairs are sufficiently proximal within the A17, H3, A27, A27 network to allow some modest direct crosslinking across their interfaces. SFEs are anchored either directly or indirectly to the core wall beneath the MV envelope by disulfide bonding (arrowed). Proteins CAHH and ATI (not depicted) are candidates for mediating such disulfides. Disulfide bonding does not involve H3 which is detergent-extractable in the absence of disulfide reduction. A27 and A26, although disulfide bonded to one another, interact tightly enough that SFEs show stability in the presence of NP40 plus disulfide reducing agent. ‘N’ and ‘C’ denote protein N- and C-termini respectively. TM protein domains are colored according to panel A and S1 Fig. Although H3 was designated ‘N-outside’ by prediction program TMHMM (opposite polarity to A17), rendering the major N-terminal domain yellow, such predictions are presumably invalid for unconventionally added (tail-anchored) TM proteins (see text). Albeit two copies of the H3-A27 trimer-A26 complex are shown to suggest SFE topology, more accurate modeling would require an understanding of protein stoichiometries.

Another clearly discernible virion protein sub-network connected membrane proteins A17, H3, A27 and A26 (Fig 8a), involving the N-terminus of A17, a central region of H3, residues ~300 to 420 of the 500 residue A26 protein and a large portion of the 110 residue A27 protein (Fig 8a). To understand this sub-network requires some consideration of the known functions and properties of the four proteins. Thus, A17, a 203 aa protein, is one of two key proteins acting at the earliest stages of virion morphogenesis (the ‘crescent’ and IV stages), the other being its partner, the 90 aa protein A14 [2]. During normal infection, A14 and A17 co-localize to ER and ERGIC membranes as well as the earliest assembly structures (‘crescents’) and IV [39, 50–54], with crescents reportedly forming via the accretion of A17-containing vesicular elements [52]. Unfortunately, no XL were detected in A14, consistent with its three crosslinkable lysines being positioned such that XL to any of them would yield a long tryptic peptide (in the top 4^th^ percentile of peptide lengths for the project). Repression of the gene for A17 leads to a blockade in virion morphogenesis at a very early stage, with membrane tubuolovesicular elements accumulating at the periphery of electron-dense virosomes/viroplasm [2, 39]. A17 has four TM domains [39] and appears to use them in a ‘reticulon’-like manner to induce membrane curvature [55]. A17’s N- and C-termini, which are trimmed *in vivo* (at residues 17–20 and 185, respectively) by I7 proteinase [47, 56–58] are both thought to be cytoplasmic. Evidence for this includes their exposure after *in vitro* expression in the canine microsomal system [50, 51, 59] and, in intact MV, accessibility to antibodies of the N-terminal 60 residue region (prior to the first membrane-spanning region starting at residue 61) [2, 60]. A17 forms disulfide bonded homodimers via Cys178 in the C-terminal tail [50], and the A17 N-terminal region interacts with D13 trimers that assemble to form the honeycomb lattice of the IV external scaffold [3, 52, 61]. Virus is excised from the scaffold in an I7 proteinase-dependent manner [62].

Protein H3 is a heparin sulfate-binding attachment protein. Although not essential for virus replication, it is required for normal plaque size and virus yield [63, 64]. It is immunodominant [65, 66], localizes to the MV surface and can be extracted therefrom with NP40 in the absence of disulfide reducing agent [67]. H3 does not seem to follow a classical protein secretory pathway, but instead seems to be post-translationally anchored to virion membranes [67], via a TM helix that is predicted to lie towards the protein C-terminus (residues 283–305 of the 324 aa protein; [68]) such that the N-terminal region of the protein is cytoplasmically oriented and exposed on the virion surface [67, 69]. H3 is added to MV within the virus factory late during maturation [67] coinciding with the replacement of IV’s D13 external scaffold with an antigenically [70] and morphologically [71] distinct surface structure (see below).

XL involving A17 included a very clear connection between the N-terminal domains of A17 and protein H3 (Fig 8a, S1 Fig). Albeit both the N- and C-terminal regions of A17 are exposed to the outside of MV [50, 51, 59], XL with H3 were detected only for A17’s N-terminal region, with none at all to A17’s C-terminal region (Fig 8a). These H3 XL were to the region of A17 remaining after proteolytic removal of A17’s extreme N-terminus during maturation. Parenthetically, additional, unrelated XL were detected to the extreme N-terminus of A17 (S1 Fig) suggesting the presence of pre-processed viroforms in the MV preparation. Interestingly, one of these XL was to the C-terminal region of A17 itself (residue 180, S1 Fig), suggesting juxtaposed A17 termini in the pre-processed form. This in turn suggests that during virus maturation, upon cleavage, and loss of the D13 exoskeleton, A17 may undergo a reconfiguration: Prior to cleavage the unprocessed N- and C-termini mutually interact but after cleavage the only the processed N-terminus can interact with H3 (see Discussion). Finally, a strongly detected (DFscore = 5) inter-subunit XL in the C-terminal region of A17 (between residue 180 and residue 180, S1 Fig) was consistent with previously reports of A17 homodimer formation [50]. No XL were detected C-terminal to the A17 C-terminal cleavage site at residue 185.

A27 is an immunodominant [72] disulfide-bonded trimer [73, 74] that can stack into hexamers and higher order multimers *in vitro* [19, 75]. It functions in virus attachment to cell surface glycosaminoglycans [76], mediating a choice between cell entry pathways [77]. It also functions in the microtubule-dependent transport of MV within the cell [78], the secondary wrapping of MV with Golgi-derived membranes late in infection to form wrapped virus (WV) [79, 80] and in cell-cell fusion [73]. A27 lacks detectable TM domains [68] but is reportedly anchored to the virion membrane via interaction with A17 [58, 75]. A27 can be removed from the MV surface by disulfide reduction [9]. Like H3, A27 is detected more strongly in MV than IV suggesting that it is added to virions during the IV to MV transition [6, 67], maybe at the same time as H3. In the current study, the A17-A27 interaction (above) was represented by just a single direct XL which, at A17 residue 36, occurred within the N-terminal “high affinity” region (32–36) noted in *in vitro* interaction studies [75]. Far more readily detectable, however, was the crosslinking of both proteins to H3 (Fig 8a) suggesting that H3 mediates a substantial portion of the A17-A27 interaction in MV.

The non-essential A26 protein mediates virus attachment to cell surface lamin [81] and the embedding of cowpox virions in A-type inclusions ([82]; discussed below). A26 is absent from wrapped extracellular virus (EV) suggesting that A26 mediates a choice between wrapping and inclusion formation. Like A27, A26 contains no TM domain [68]. Instead, A26 is anchored to the MV membrane via disulfiding of cysteines 441 and 442 in its C-terminal coiled-coil region with Cys71 and Cys72 towards the C-terminus of A27 [83]. Via these interactions A26 and A27 are tethered to one another and to protein A17 on the virion surface [84]. Here, A26-A27 XL were detected abundantly, at sites in both proteins immediately N-terminal to the above-mentioned cysteines (Fig 8a). As with the A17-A27 interaction, some mediation of the A26-A27 interaction by H3 was suggested by the crosslinking pattern (Fig 8a).

The replacement of IV’s D13 external scaffold, during late-stage maturation, with a distinct surface protein structure over the MV lipid envelope (above) is supported by substantial evidence. Firstly, a two-domain exterior boundary is visible in thin sections of MV [2]. Second, AFM imaging under ambient conditions in the absence of any virion pre-treatment shows a surface topography described as resembling “surface fibrous elements” (SFEs) [15]. Deep-etch electron microscopy (DEEM) [3, 85], which involves neither fixation nor negative staining, evokes comparable descriptions of the MV surface (disorganized, close-packed, parallel rows of short “railroad tracks” [3]). These patterns also reflect the “Mulberry-like” MV surface features imaged by high-contrast negative staining as described throughout the literature [2, 85–91]. SFEs can be detached from the virion surface via the action of disulfide reducing agent in the presence of NP40 (they remain nominally intact in the presence of both reagents), and appear compellingly similar when imaged by either EM [91] or AFM [15]. They have been described as chain-like, globular protein fibers of uniform size (20 nm diameter x 100–150 nm length) with no obviously hollow interior or helicity [15]. Overall, the late adherence of H3 and A27 to the MV envelope (above) coinciding with the appearance of the mature surface topology, in combination with the crosslinking pattern of Fig 8a suggests that SFEs, comprising or containing the A27/A26 complex, are brought to the MV surface via the late tail-anchoring of H3, and that H3 mediates the interaction of SFEs with A17 already present at the MV envelope. This scheme is depicted in Fig 8b. Although H3 was designated ‘N-outside’ by prediction program TMHMM, which would polarize H3 with its major N-terminal domain in the ER lumen in the conventional secretory pathway and thence on the inside of the MV envelope, the prediction probability was little better than evens (62% [68]) and moreover, such predictions are presumably invalid for unconventionally added (tail-anchored) TM proteins.

#### The contrasting crosslinking patterns of proteins H3 and L1

Protein H3 showed 119 distinct contacts with other proteins (S1 Fig). This was a remarkable number, suggesting a high degree of connectivity for H3 in comparison with other proteins considered functionally comparable and/or that may be in the same compartment such as L1 (below). Quite dramatically, no inter-protein XL were detected beyond residue 266 of the 324 residue H3 protein, thereby restricting all inter-protein XL to H3’s 282 residue N-terminal ‘outside’ domain. The diversity of contacts suggested that protein H3 may sample multiple, complex environments. H3 also showed an unusually high signal for homomultimer formation (S2A Fig, red loops). Since the published crystal structure for H3, covering residues 1–237 did not show a homomultimer [92], we speculate that either the homomultimer interface is to the C-terminal side of the crystalized region, or H3 forms a mixed multimer (eg. A2B2-type) or H3 crosslinking to itself results from a very dense packing of monomers within virion membranes. Homomultimer formation may be consistent with H3 nucleating SFE formation (above).

Somewhat surprising, and in contrast to the 119 distinct inter-protein contacts involving TM protein H3, was the absence of TM protein interactions observed for the myristoylated [93] immunodominant TM protein L1, whose only detected inter-protein contact was with protein A12 (S1 Fig). Like H3, L1 appears on the MV surface later during virion maturation, after departure of the D13 external scaffold, via a C-terminal anchoring domain [94]. Yet L1 appears to be remarkably isolated on the MV surface from other TM proteins and virion proteins in general. Alternatively, there may be a greater difficulty in detecting L1 XL due to, speculatively, a differential abundance of H3 and L1 in MV.

#### All detected XL between TM proteins

**Fig 9:**
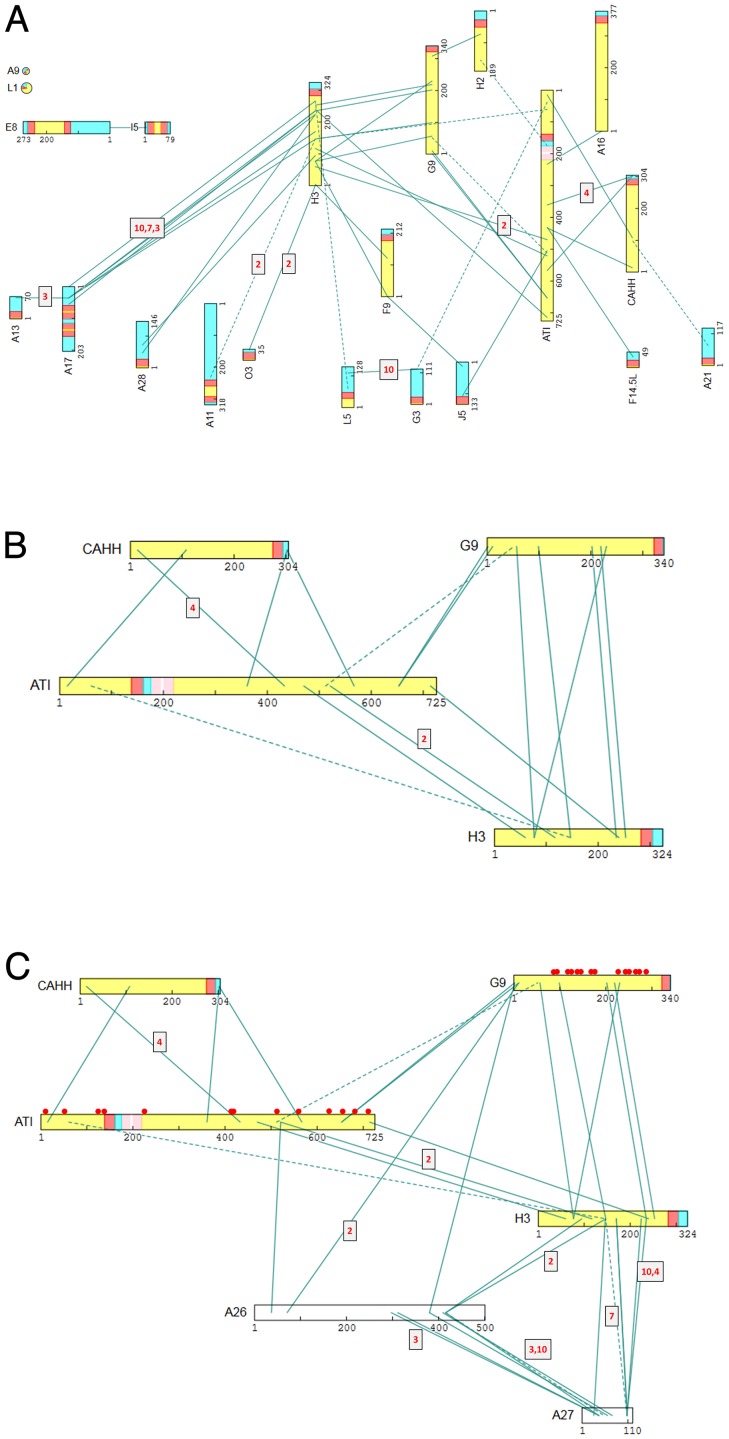
(**a**) All XL detected between virion TM proteins. Coloration as in Fig 8. Upper area: Proteins with XL to predicted ‘outside’ domains. Lower area: Proteins with XL primarily to predicted ‘inside’ domains. No XL were detected between protein A9 and L1 (upper left) and other members of the “Membrane” group, no XL at all were detected for A14 or I2 (not depicted) and protein A26, A27 and VENV(F13) are considered to be membrane-associated only. The remaining 21 TM proteins of the “Membrane” group are shown. ‘Minor’ predicted TM domains of ATI are colored pink. (**b**) ‘Major outside protein’ sub-network (proteins ATI, CAHH, G9, H3). Domain coloration as in panel A. (**c**) Six-member ‘attachment protein subnetwork’ (‘Major outside protein’ plus proteins A26, A27). Domains are colored as in panel A. Red circles: Positions of cysteines within the cysteine-rich proteins ATI and G9. Other details as in Fig 5.

For the majority of virion proteins with predicted TM domains, intra-protein XL were confined to either one or both sides of the predicted TM domain and not within or across it (S2A Fig). This pattern supported independent predictions of TM domain locations within TM proteins and suggested a propensity for crosslinkers to not act across lipid bilayers. Fig 9a shows all detected XL between TM proteins in the ‘membrane’ protein group (Table 3). TM proteins could be divided into two subsets whose major portions were classified as either ‘outside’ or ‘inside’ [68, 95] (Fig 9a, upper and lower regions respectively). Inter-TM protein XL appeared to involve only the major portion of each TM protein (Fig 9a), with extensive inter-protein crosslinking observed within the ‘outside’ subset, and also between the ‘outside’ and ‘inside’ subsets. A major contributor to the latter class was the cluster of A17-H3 XL discussed above. Other clear contacts at the interface of the two subsets mainly involved proteins H3 and ATI (ATI has been referred to by others as ‘A25’: In our notation, ‘A25’ refers to protein A2.5), and included the following connections: A28-H3, O3-H3, L5-H3, J5-F9, J5-ATI, F14.5-ATI G3-ATI and A21-CAHH (CAHH has been referred to by others as ‘D8’). The only direct XL observed within the ‘inside’ subset were a very highly detected XL between proteins L5 and G3 and a contact between A17 and A13 (Fig 9a, lower region).

As mentioned above, late in infection, for the purpose of virus dissemination, cowpoxvirus forms A-type inclusions by the coalescence of the non-essential cowpox ATI protein followed by virus embedding in the resulting inclusion via protein A26. Vaccinia ATI is a C-terminally-truncated version of the cowpoxvirus protein which cannot form inclusions. Vaccinia ATI is included in the TM protein set because of its predicted possession of a TM domain with 80% probability, with a 77% probability of ‘N-inside’ polarity [68] (S2B Fig). The predicted TM domain, located between residues 139 and 161, is flanked to the C-terminal side by two minor ones (20% probability, S2B Fig). Since Vaccinia ATI stood out among TM proteins in terms of the many (10 or more) XL spanning the predicted TM domain(s) (S2A Fig), we designated both the C-terminal and the N-terminal region as ‘outside’ (S2C Fig, Fig 9). This topology may arise if Vaccinia ATI were either not a TM protein at all (it may have no known functional requirement to be one), or if it were double-spanning (via the major one noted above plus one or both of the minor predicted TM domains), or if it were membrane-anchored via the multiple (six) acylation/myristoylation sites previously noted within the C-terminal half of the protein [96].

Within the ‘outside’ subset of TM proteins(above), multiple XL were strongly-detected between ATI and CAHH, H3 and G9 (Fig 9a and 9b). Into this ‘major outside protein’ sub-network could be plugged the membrane-associated proteins A26 and A27 to form a clear 6-member ‘attachment protein sub-network’ (Fig 9c). Of MV’s four known attachment proteins, H3, CAHH, A27 and A26 (the first three binding to cell surface glycosaminoglycans (GAGs) [64, 76, 97] and the latter binding extracellular matrix laminin [81]), all four were present in the ‘attachment protein sub-network’, supplemented by ATI and G9 (Fig 9c). The latter two may play a scaffolding role: Both are acylated/myristoylated and cysteine-rich (13 and 14 cysteines respectively), providing ample potential disulfide anchoring points to the virion infrastructure. Within this sub-network, only CAHH appeared to be disconnected from a direct interaction with H3, whose ‘outside’ domain otherwise formed a ‘hub’ for the 6-protein sub-network (Fig 9c). This was consistent with the role of H3’s outside domain as a hub for virion membrane proteins more generally (Fig 9a). In terms of previously known interactions among the six proteins: The cowpox ATI-A26 interaction has been demonstrated to require the 100–300 aa region of cowpoxvirus ATI [82]. A corresponding XL was detected, here, between positions 37 and 521 of A26 and Vaccinia ATI, respectively (Fig 9c, S1 Fig). By co-IP analysis, A26 has been reported to interact with G9 and A16 [98]. The A26-G9 interaction was manifest here via the N-terminus of G9 (Fig 9c, S1 Fig). Of the five known TM protein substrates for the Vaccinia-encoded redox system namely L1, A28, A21, L5, and H2 [2, 99], all except H2 and the orphan L1 protein (above) appeared in the ‘outside’ subset.

#### Two previously reported complexes

**Fig 10:**
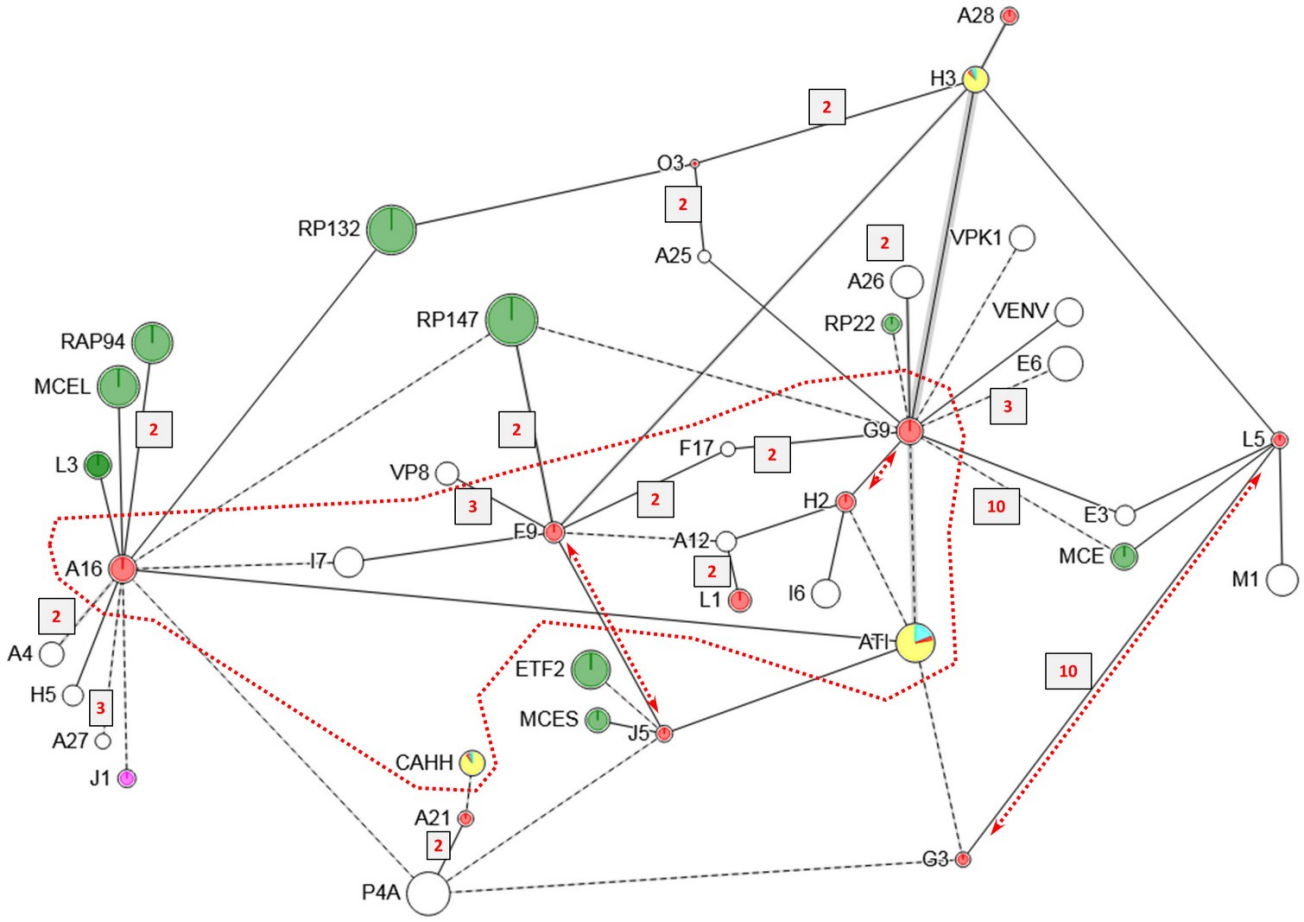
XL among EFC proteins and their detected partners (see also Table D in S1 Text). Proteins are depicted as circles whose areas correspond to chain length. Red fill: EFC proteins. Green fill: Proteins of mRNA biogenesis. TM proteins are multi-colored as described under Fig 8. Boxed numbers (red font): XL with DFscore > 1. Red dotted perimeter separates EFC proteins falling in the ‘outside subset’ and the ‘inside subset’ (inside and outside the perimeter, respectively). Double-ended red arrows: Three direct EFC-EFC protein interactions detected by crosslinking. Other details as in Fig 5.

EFC: A virion ‘entry-fusion complex’ (EFC) has been proposed comprising nine central components (proteins A16, A21, A28, G3, G9, H2, J5, L5 and O3) [100]. MV with mutations in these proteins are morphologically normal and transcriptionally active, and can undergo normal membrane wrapping and export from the cell. They can also bind the cell but are defective in penetration [2]. Loss of any one EFC component does not appear to affect the incorporation of others [2]. Two additional EFC-associated proteins (L1 and F9) are also required for cell entry by the virus but are not required for assembly or stability of the core EFC complex [100]. Fig 10 shows the 11 proteins and their detected crosslinking partners. All TM protein partners within the EFC are summarized in Table D in S1 Text. Of three previously reported EFC-EFC protein interactions (Table D in S1 Text, [100]) one (G3-L5) was confirmed by crosslinking (with a high DF score, Fig 10, Table D in S1 Text). Crosslinking led to the detection of two additional EFC-EFC protein interactions, namely F9-J5 and G9-H2 (Fig 10, Table D in S1 Text) in which the N-terminus of F9 connected directly to the N-terminal region of J5, and the C-terminal region of G9 connected directly to H2. At the current detection depth, other EFC members’ connections appeared to be mediated by third-party proteins.

Protein ATI appeared to mediate the connection of five EFC members, namely A16 (N-terminal region), H2 (C-terminal region), G3 (C-terminal region), G9 (N-terminal region) and J5 (C-terminal region). TM protein H3 also appeared to mediate the interaction of five EFC members, namely A28, F9, G9, L5 and O3, three of which (A28, F9, G9), showed multiple connections to H3 (S1 Fig) and may therefore have a more intimate H3 connection. Interestingly, EFC protein G9 was common to both the ATI and H3 sets, with ATI crosslinking to G9’s N-terminal region, and H3 interacting across a broad swath of the 340 aa G9 protein.

Overall, the EFC may fall into two parts (distinguished in Fig 10 via a red ring), namely, proteins from the ‘outside’ subset of Fig 9a (A16, F9, H2, G9 plus L1) and those in the inside subset (O3, A28, L5, G3, J5, A21), the former perhaps being tail-anchored and added late during virion maturation.

7PC: Another reported complex in the virion is the ‘seven protein complex’ (7PC) [101] mutations in whose members (A15, A30, G7, J1, D2, D3 and VPK2) have similar phenotypes relating to the association of viroplasm with growing crescents during early virion morphogenesis, and the appearance of vestigial IV. For example, A30 mutants show enlarged virosomes and empty IV or pseudo-IV with multiple membrane wrappings [102, 103]; G7 is required for the movement of crescents to the periphery of virosomes and the filling of crescents with viroplasm [104, 105]; the repression of J1 phenocopies the repression of A30 and G7 [106, 107], and A15 mutants show characteristic empty IV [101]. Association of the seven proteins in a common complex has been deduced by mutual pullouts from infected cell extract with epitope tagged A15, VPK2, D2 and D3 followed by immunoblotting for the other complex members [101]. Regarding direct interactions, A30 was shown to interact directly with G7 [104], proteins A30 and G7 both become unstable in the absence of J1 at the restrictive temperature [106, 107], and J1 has been shown to self-interact ([20, 107].

**Fig 11:**
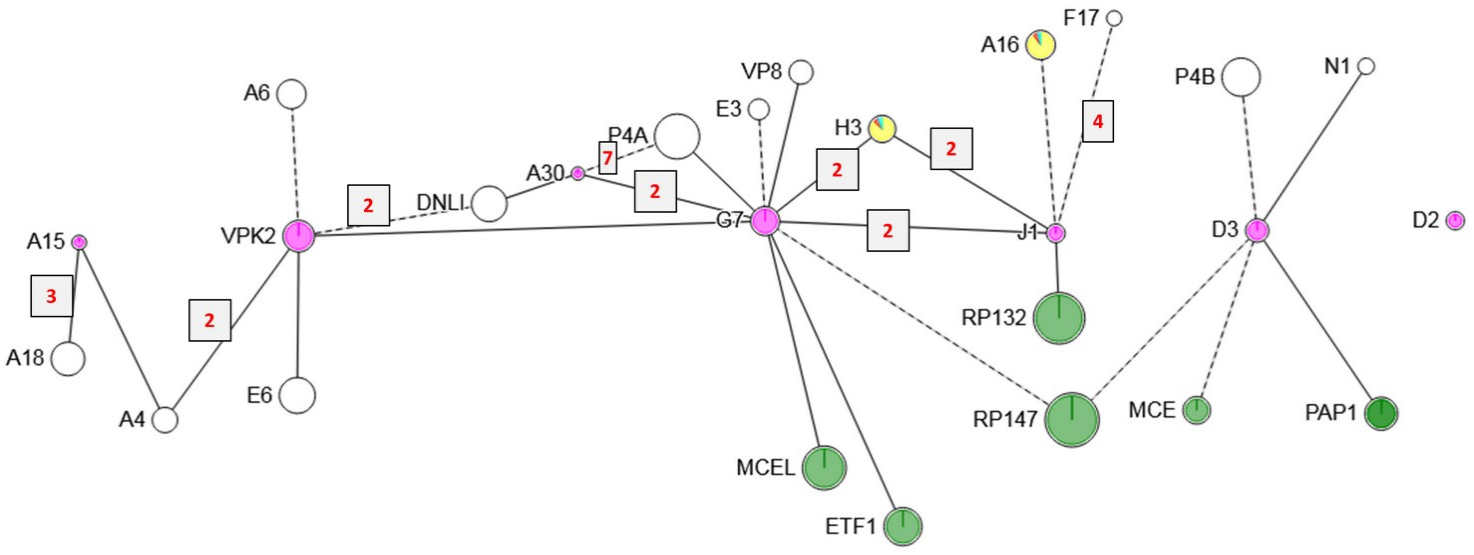
XL network among 7PC proteins and their detected partners. Proteins are depicted as circles whose areas correspond to chain length. Magenta fill: 7PC members. Other details as for Figs 10 and 5.

Fig 11 shows 7PC members and their crosslinking partners. No crosslinking partners were detected for protein D2. The remaining six proteins could be linked via three direct XL (G7-VPK2, G7-A30 and G7-J1) and two mediated contacts, namely via the extreme N-terminus of protein A4 and the N-terminal region (aa 54–75) of RP147. Protein G7 appeared as a ‘hub’, directly linking three other 7PC members (VPK2, A30 and J1), and perhaps interfacing an A15/VPK2/A30 sub-complex (nucleated at an N-terminal crosslinking hotspot of G7; Fig 11 left side) with a J1/D3 sub-complex contacting the C-terminal half of G7 (Fig 11 right side). Within the A15/VPK2/A30 sub-complex, structural protein A4 crosslinked to both A15 and VPK2. In addition, p4a fragment 3 crosslinked to G7 and A30. Consistent with the latter observation, p4a mutants show an aberrant and ‘empty’ IV phenotype [108] reminiscent of the 7PC mutants themselves. Contacts with the transcription/mRNA biogenesis apparatus appear to cluster around the J1/D3 sub-complex within which D3 appears to connect to both subunits of the PAP heterodimer (PAP1/MCE, Fig 11). With D3 also contacting protein p4b (which appears to be located predominantly at the interior face of the core wall, see Fig 6 and associated text) it is interesting to consider 7PC as perhaps a core wall-spanning complex. Regarding the J1/D3 sub-complex, the N-terminal region of TM protein H3 crosslinked to sites in J1 and G7 very close to the sites at which these two proteins crosslinked to one another.

#### Virion proteins of DNA binding/metabolism

**Fig 12:**
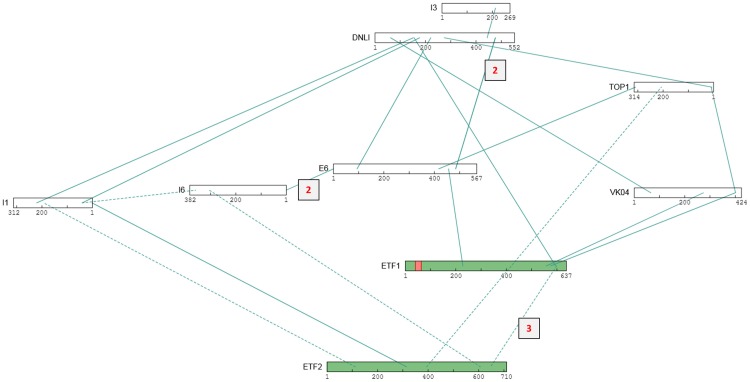
XL sub-network that includes ‘telomere-binding’ and other proteins from the ‘DNA’ functional group. Two transcriptional DNA binding proteins are also included, namely ETF1 and ETF2 (the early transcription factor heterodimer) along with protein E6. Other details as in Fig 5.

Vaccinia DNA ligase (DNLI), a nick sealing protein, showed 22 crosslinking partners (S1 Fig)—an unexpectedly large number for a specialized enzyme. Among these were four other members of the DNA binding/DNA metabolism group (Table 3), namely K4—the Vaccinia DNA nicking enzyme for genome telomeres [109], I1 –a Vaccinia telomere binding protein, I3 –an ssDNA binding protein [110, 111] and Vaccinia topoisomerase TOP1. Moreover, direct crosslinking was detected between I1 and I6, both of which are known to bind Vaccinia genome telomeres ([2, 109] and references therein). Fig 12 shows a crosslinking sub-network encompassing proteins DNLI, I1, I3, I6, K4 and TOP1, along with three proteins that seemed to couple quite well with the above network, namely the two subunits of the Vaccinia transcription factor heterodimer (ETF1 and ETF2) and protein E6.

We note that XL were detected very strongly between telomere-binding protein I1 and a centrally-located ‘outside’ domain of the double membrane-spanning TM protein E8 (S1 Fig). E8 is retained with the virion core in the presence of the core-stripping/activating reagent combination NP40/DTT, binds single-stranded DNA *in vitro* [112], and has been proposed to connect the viral genome with ER and viral membranes ([112, 113] and references therein). The very strongly detected XL between E8 and I1 suggested the targeting of genome telomeres by a complex containing E8 and I1. Four of E8’s 11 detected protein crosslinking partners were transcription-related (S1 Fig), all of whose XL were to E8’s terminal domains representing the opposite compartment to that targeted by protein I1 (S1 Fig). This raised the possibility that, in MV, the genome telomeres may be somehow membrane-compartmentalized away from the transcriptosome plus the bulk of the genome destined to be transcribed by it during early infection. This scenario lacks complete physical clarity, however, since the sub-network of Fig 12 would place the early transcription factor ETF in the telomere compartment while the crosslinking pattern for E8 (S1 Fig) would place the transcriptosome outside it. A provocative solution would place the dual-role (morphogenesis and early transcription) ETF in both compartments. Interestingly, virions from the temperature-sensitive mutant tsE8 assemble normally, but exhibit a transcriptionally inactive core [2]. We note that two topological polarities have been proposed for E8 and its interactor I5 as mentioned in the E8 section of S1 Fig.

#### Other enzymes and proteins

DUSP, the Vaccinia dual specificity phosphatase (H1) is reportedly resident in the lateral bodies of MV [10]. Although DUSP targets viral and cellular proteins and can dephosphorylate Vaccinia proteins A17, A14, and F17 *in vitro* [2], no interaction with these three substrates was detected here. Instead, XL were detected quite strongly between the C-terminus of DUSP and ATI and CAHH proteins of the attachment complex (Fig 9b and 9c), along with transcriptosome proteins RP132 and ETF2.

Mutants in the I7 cysteine protease are defective in the IV/IVN to MV morphogenic transition and in the processing of major structural proteins p4a, p4b and A4, and the TM protein A17 (A17 being processed earlier during morphogenesis and not subject to a Rifampicin blockade [2]). None of these proteins was represented among the crosslinking partners of I7 detected here (S1 Fig) perhaps because they are, in MV, enzymatic products rather than substrates. Among the six TM and membrane-associated XL partners for I7 was the TM protein H3 (discussed above), whose ‘outside’ domain crosslinked, in a strongly-detected fashion, to two positions towards the C-terminus of I7. This region of H3 also crosslinked to A17, raising the possibility of H3 providing a scaffold for I7 protease, employed in the AG|-dependent removal of the A17 N-terminus. Crosslinking of I7 to proteins PAP1, F9, A18 and A13 involved the extreme C-terminal regions of each partner; I7 crosslinking to RP35, E6 and CAHH involved the extreme N-termini of these partners. Like H3, none of these proteins contains an AG diamino acid target for I7-dependent proteolysis, and these XL could represent merely the flexing of protein termini.

In virions with repressed G1 metalloprotease, which is defective in the IV to MV morphogenic transition, P4a, P4b, VP8, G7 or A17 precursors are cleaved normally [114], albeit VP8 is cleavable by G1 in a transfection assay [115]. In apparent contrast to this, XL were detected between G1 and each of the three major structural proteins p4a, p4b and A4 though not VP8 (S1 Fig).

The C-terminus of the 65 aa F8 protein appeared as a Y2H interactor with VP8(L4) [20]. Consistent with this, crosslinking was clearly detected between the C-terminal regions of F8 and VP8 (S1 Fig).

#### Inter-protein XL partitioning analysis

S3 Fig shows the results of inter-protein XL partitioning analysis (see Materials & methods) for key structural and membrane proteins. For proteins showing higher bars +NP40 and/or +TCEP than -NP40 and/or -TCEP, a barrier to crosslinker access is apparently dissociated by NP40 and/or TCEP. Among the DNA group proteins, inter-protein XL involving telomere-binding protein I1 fell into this category, consistent with a “telomere” compartment in the virion that can be exposed upon treatment with NP40/TCEP. The barrier is not necessarily lipid, since the crosslinker used for the majority of experiments, namely DSS, is membrane-permeable [116]). By contrast, for proteins showing higher bars -NP40 and/or -TCEP, either these proteins or their crosslinking partners are apparently lost by pre-treatment with these reagents. Among the structural proteins, inter-protein XL for VP8 and A12 seemed strongly favored by -NP40 and/or -TCEP as were, to some extent, P4a and p4b suggesting that pre-treatment results in the extraction of either these proteins or their partners. By contrast, A4 and F17 (which are located on the exterior core wall surface, above [9]) were indifferent to pre-treatment (S3 Fig). In the membrane protein group, the interactions of proteins A26 and A27 with external partners seemed likewise sensitive to the presence of NP40 and/or TCEP while ATI and CAHH were indifferent. A26/A27 are known to be disulfide-bonded to the virion surface [83]. Overall, these result seem consistent with A26/A27 and VP8/A12 being connected to one another and to p4a/b via reducible disulfide bonds, while A4, F17, ATI and CAHH(D8) seem more resistant to disulfide reduction and/or may be anchored in other ways.

## Discussion

We have investigated the molecular structure of the Vaccinia virion, a highly non-stoichiometric protein assembly, via XL-MS. Analysis of protein-protein interactions in the virion *in situ* avoided the need for their preservation during virion extraction with reagents such as deoxycholate, an ionic detergent used for the release of virion core enzymes [16]. There was no requirement to rebuild virus protein complexes *de novo*, avoiding a need for the correct folding of challenging or insoluble structural proteins *in vitro* and/or in a heterologous system. Finally, multivalent/higher order complexes could be addressed that were not accessible via binary assays such as Y2H [20].

As in any XL-MS study, challenges included: The availability and appropriate spacing of crosslinkable sites at protein interfaces; good occupancy of crosslinking sites and robust reaction of both ends of the crosslinker; efficient laboratory digestion of crosslinked proteins (given the tendency of trypsin recognition sites, for example, to become derivatized); the detection of crosslinked peptide pairs against a large excess of non-crosslinked peptides in the same digest; rarity of inter-protein XL (the most informative kind) with respect to other kinds (intra-protein, intra-peptide, and single-ended XL); the tendency of large (more than double-size) crosslinked peptide pairs to ionize less efficiently during MS; inefficient fragmentation and combinatorial complexity of fragment ion mass spectra when simultaneously fragmenting peptide pairs, and the challenge of distinguishing true intra-molecular XL from those that may cross homomultimer interfaces. For Vaccinia as a target, the above issues were compounded by: Unknown permeability of the virion core to crosslinker; a protein abundance dynamic range in Vaccinia MV of 5000-fold [34] or more; a paucity of existing high resolution protein structures for validation, and the possibility of molecular heterogeneity arising from mixed viroforms in MV preparations and/or mixed proteoforms within a single particle.

Addressing the above challenges (most particularly the abundance range and sensitivity issues) we adopted a “strategy of experimental variation”, as explored initially in our analysis of the MV phosphoproteome [117]. For XL-MS this strategy involved a ‘multithreaded’ workflow (Fig 1) in which experimental steps were matrixed combinatorially (Table 1). In this way, individual XL were placed in a variety of ionic contexts for MS detection, and key interfaces were painted as clusters of alternative XL between closely spaced crosslinking sites. This was combined with the use of diverse XL search engines for the identification of crosslinked peptides, and the use of isotopically coded crosslinkers where available. Our 86-protein search database comprised the maximum set of viral proteins considered likely to be packaged [5]. For all but two of these proteins XL were detected, the exceptions being proteins A14 and I2. These two short proteins (90 aa, 73aa respectively) possess relatively few sites for crosslinking and trypsin cleavage (3 lys/2 arg; 4 lys/0 arg, respectively).

Due largely to the absence of strong corroborating data for our XL-MS dataset such as comprehensive atomic-resolution three dimensional structures, validation relied largely on statistics and trends. The effective FDR of 0.33% for the final dataset as a whole (“Results”), suggested a remarkably low level of bioinformatics noise. Consistent with this, non-target databases from uncorrelated proteomes, namely all human proteins or the non-packaged subset of Vaccinia proteins yielded very weak results in preliminary searches.

Alongside the detection of clear crosslinkome sub-networks (“Results”) were many single-detect inter-protein XL (DFscore = 1, S1 Fig). Notwithstanding the excellent bioinformatic signature for the dataset as a whole (above), it was difficult to ascertain to what extent the single-detect XL were real (from, for example, low abundance proteins, low abundance viroforms, inefficient XL, or poorly ionizing peptides), or represented biochemical noise (eg. virion dissociation pathways during virus preparation or specific experiments). On the one hand, evidence that single-detect XL were true positives included the tendency of single-detect crosslinking patterns within a protein sub-network to conform to patterns of XL with higher DFscore. For example, among the 22 inter-protein XL shown in Fig 5a, 18 were multi-detects vs. 8 single-detects, all contributing to the same overall crosslinking pattern. On the other hand, high DFscoring XL showed a higher ratio of biologically rational:non-rational XL than did single-detect XL, lending greater confidence to former. For example, among the 37 inter-protein XL in the dataset with DFscore > 5 (Table E in S1 Text), the number that were considered biologically rational exceeded the number designated non-rational by a factor of 9.5 while, among the single-detect XL from the same table, rational exceeded non-rational XL with a factor of only 1.5.

Transcriptosome proteins, albeit presumably packaged in relatively low abundance, nonetheless showed a number of strongly detected inter-protein XL (Tables F, G in S1 Text). Some of these, including some of the most strongly detected inter-protein XL in the dataset (S1C Table; Table F in S1 Text, orange), were between transcriptosome and membrane proteins (Table F in S1 Text), including ectodomains of the latter. These XL were considered biologically “non-rational” (above) since the transcriptosome is located within the virion core while the TM proteins surround it according to conventional models. They were strongly supported by their DFscores, were not filterable by raising score thresholds, and their DFscores did not drop when switching between singly- and dually-thresholded filtering (Materials & methods). We were therefore unable to falsify a hypothesis that contacts can occur between transcriptosome components and the ectodomains of membrane proteins, the significance of which is unclear. Possibilities for these resilient, yet ‘non-rational’ XL may include that: (a) the core wall is not a fundamental barrier to crosslinking (it is porous)—indeed the 7 nm inside-diameter pores that have been imaged in the core wall [9, 118] may be sufficiently large for the majority of Vaccinia polypeptides to pass through entirely if they are globular and approximately spherical [119], (b) TM and transcriptosome proteins are both implanted in the barrier (from opposite sites)–a situation, on the transcription side, observed in the cores of turreted Reoviruses [120, 121], (c) TM proteins are located in more than one compartment, (d) MV preparations contain developmental viroforms from a time prior to the full emergence of the core wall, (e) they are cryptically artefactual.

The dataset contained evidence for viroforms/proteoforms from proteolytic maturation. Peptides crossing known [2] sites of viral AG| specific proteolytic processing in proteins A17, VP8, G7, p4b and p4a (site2) can be found in tryptic digests of purified MV [5]. These peptides represent pre-cleaved proteoforms. Such peptides were also found in the current study, within crosslinked pairs, from proteins A17, VP8, A12 and G7 (Table H in S1 Text). XL connecting the N-terminal amino group of pre-cleaved A17 with the C-terminal region of the same protein (“Results”) may be an example of the same phenomenon. In some cases the crosslinker directly spanned an AG| processing site. Apparently, then, MV harvested from Hela cells late in infection followed by 2x sucrose gradient-purification were accompanied by immature viroforms that are detectable by highly sensitive MS. Among crosslinked peptides could be found no trace, however, of a characteristic and abundant marker of IV, namely the external scaffold protein D13 when using XL search databases that included this protein. Apparently, in MV, in which the external scaffold, along with fragment 2 of protein p4a (Fig 5b) are close to or below the detection limit, unprocessed forms of proteins A17, VP8 and A12 are still readily detectable. If, speculatively, MV preparations contain trace viroforms that appear morphologically mature (having already escaped the external scaffold and perhaps received tail-anchored and SFE proteins), but which still lack a fully formed interior and/or an impermeable core wall, then this may account for some of the more counter-intuitive XL detected here. Alternatively, some XL may represent structures that appear only transiently in the virion maturation pathway. Another possibility may be that MV particles, albeit fully mature, retain unprocessed proteoforms by design. Within an A17 homodimer, for example, one subunit might be processed and the other not.

Evidence for multiple viroforms/proteoforms also arose from interactions between p4a, p4b and TM proteins: p4a fragment 3 was found to be within crosslinking range of seven distinct membrane proteins (S1 Fig, Fig 6) and also within crosslinking range of the C-terminal region of p4b, while none of the seven membrane proteins were apparently within crosslinking range of p4b Fig 6, S2A and S2B Fig). Moreover, a crosslinking ‘hotspot’ in p4a fragment 3 (K736) interacted with three membrane proteins as well as p4b (S1 Fig), in the absence of any detectable crosslinking between the latter. While steric factors may allow p4a, p4b and membrane proteins to triangulate in a way that leaves all membrane proteins out of range of p4b, it seems also possible that membrane proteins and p4b may interact with alternate proteoforms of p4a. This could result from distinct and segregated p4a complexes within individual MV, or distinct viroforms in the virus preparation (e.g. the rearrangement of p4a fragment 3 during maturation).

In conclusion: Here, we have covered the crosslinkome of a relatively small whole organism in depth, detecting inter-protein XL for all but two of the 86 proteins that represent the maximal virion proteome. Strategies were developed to detect XL in a proteome covering a wide abundance dynamic range and with minimal pre-existing crystallographic information, allowing the reconstruction of several key virion protein complexes. The challenge of synthesizing the data into an extended understanding of the internal molecular architecture requires some knowledge of intra-particle protein stoichiometry.

## Materials & methods

### Materials

Vaccinia virus was purified by sucrose or tartrate gradient as described [5] and protein quantitated using BCA (ThermoFisher Inc.), determining concentrations to be between 1 and 3.5 mg ml^-1^. DSS-H12, DSS-D12, DSG-H6, and DSG-D6 were obtained from Creative Molecules Inc. BS3-H4, BS3-D4, BS(PEG)5, BS(PEG)9, Zeba Spin Desalting Column (7K MWCO), and Lys-N were obtained from Thermo Scientific. DSS, bis(sulfosuccinimidyl)suberate (BS3), and disuccinimidyl glutarate (DSG) were used as 1:1 mixtures of DSS-H12/DSS-D12 (‘DSS-H12/D12’), BS3-H4/BS3-D4 (‘BS3-H4/D4’), and DSG-H6/DSG-D6 (‘DSG-H6/D6’) respectively. Trypsin, dimethyl sulfoxide (DMSO), Benzonase, iodoacetamide, n-LS, adipic acid dihydrazide (ADH), 4-(4,6-dimethoxy-1,3,5-triazin-2-yl)-4-methylmorpholinium chloride (DMTMM), 1-[bis(dimethylamino)methylene]-1H-1,2,3-triazolo[4,5-b]pyridinium 3-oxid hexafluorophosphate (HATU), and cyanogen bromide (CNBr) were from Sigma-Aldrich. GluC, AspN, LysC, LysN and ArgC were from Promega. AspN was from Roche Diagnostics. C18 and SCX filters were obtained from 3M. N,N-Diisopropylethylamine (DIPEA) was from Alpha Aesar. Centrifugal concentrators (Vivacon, 10kDa MWCO) were from Sartorius Stedim Biotech.

### Virus pre-treatment

Prior to crosslinking, virus was washed 5x with phosphate buffered saline (PBS), pH 7.4, by centrifugation and resuspension. For some experiments, washed virus pellets were then resuspended in 10 μL of 0.1 M triethylammonium bicarbonate (TEAB, pH 8.5) and supplemented with an equal volume of 2x ‘pre-treatment’ buffer comprising either 0.1 M TEAB, 0.1% NP40 (pH 8.5), or 0.1 M TEAB, 0.1% NP40, 80 mM TCEP (pH 8.5), followed by 2 min incubation.

### Amine-amine crosslinking

The method of ref. [122] (‘xQuest crosslink method’) was used with some modifications. Pre-treated virus suspension (above), or intact virus suspended in 0.1 M TEAB (pH 8.5), was supplemented with 1/10 volume of 10x crosslinking buffer (0.2 M 4-(2-hydroxyethyl)-1-piperazineethanesulfonic acid (HEPES), KOH, pH 8.2). Crosslinker, dissolved freshly in DMSO, was then added at a final concentration of 7.5 mM. Following 30–60 min incubation at 37°C, samples were quenched by adding 1 M ammonium bicarbonate (AmBic) to a final concentration of 50 mM followed by 30 min incubation at 37°C.

### Carboxyl group crosslinking

ADH with either HATU/DIPEA or DMTMM: Pre-treated virus suspension (above) was supplemented with 10x ADH-XL buffer (0.2 M HEPES-NaOH, pH 7.2) to 1x ADH-XL buffer (final) then supplemented with ADH and HATU (dissolved separately in 1x ADH-XL buffer) to final concentrations of 6 mM and 9.2 mM respectively. 100% DIPEA was then added to a final concentration of 46 mM. After 120 min incubation at room temperature with continuous shaking, crosslinked virus was exchanged into 50 mM AmBic using a spin desalting column (Zeba, ThermoFisher, Inc.) following the manufacturer’s instructions. In some experiments, HATU/DIPEA were replaced with DMTMM, using concentrations of ADH and DMTMM described [123].

ADH/EDC/NHS or EDC/NHS alone: Pre-treated virus suspension (above) was supplemented with 10x ADH-XL buffer (0.2 M HEPES-NaOH, pH 7.2) to 1x ADH-XL buffer (final) then supplemented with ADH (dissolved separately in 1x ADH-XL buffer) to a final concentration of 6 mM. N-(3-dimethylaminopropyl)-N′-ethylcarbodiimide hydrochloride (EDC) and N-hydroxysuccinimide (NHS), dissolved separately in 1x XL buffer were then added at final concentrations of 8 mM and 10 mM, respectively. After 120 min incubation at room temperature, free crosslinker was removed by spin desalting into 50 mM AmBic (above).

ADH with EDC or EDC alone: Pre-treated virus suspension (above) was supplemented with 10x MES buffer (0.1 M 2-(N-morpholino)ethanesulfonic acid (MES), 20 mM NaCl, pH 4.7) to 1x MES buffer (final), then supplemented with ADH and EDC (dissolved separately in 1x MES buffer) to final concentrations of 6 mM and 2 mM, respectively. After incubation for 120 min at room temperature, free crosslinker was removed by spin desalting into 50 mM AmBic (above). For some experiments (EDC-alone crosslinking) ADH was omitted.

### Solubilization of crosslinked virus for protease digestion

Crosslinked virus samples in 50 mM AmBic were disaggregated by supplementing with 0.5 M TCEP, 1 M TEAB and solid urea or guanidine, then diluting to achieve a final formulation of 8 M urea, 0.1 M TEAB, 10 mM TCEP, pH 8.5 (urea buffer) or 6 M GuHCl, 0.1 M TEAB, 10 mM TCEP, pH 8.5 (guanidine buffer). In some experiments, crosslinked virus suspension in 50 mM AmBic was instead supplemented with an equal volume of 2x detergent solution to achieve 0.5% sodium deoxycholate (SDOC), 12 mM n-laurosarcosine (n-LS), 5 mM TCEP, 50 mM TEAB, pH 8.5 (final). After 30 min incubation at 37°C, some samples were alkylated with iodoacetamide at either 5 mM (if supplemented with urea or GuHCl), followed by 30 min incubation in the dark) or 10 mM (if supplemented with detergents), followed by 15 min incubation in the dark. Some samples were then incubated with Benzonase (250 units) for 60 min. All samples were then diluted with 50 mM AmBic for cleavage, according to the manufacturer’s recommendation for tolerable denaturant (below).

### Cleavage

Cleavage employed the following reagents/reagent combinations: Trypsin, ArgC, GluC, AspN, LysN, LysC, or Trypsin+GluC, Trypsin+AspN, ArgC+AspN, ArgC+GluC, AspN+GluC or CNBr+Trypsin.

For digestions containing GluC, samples were diluted to a final urea concentration of 0.5 M. For digestion with LysN, samples were diluted to a final urea concentration of either 1 M or 5 M. For all other proteases, samples were diluted to final denaturant concentrations of either 0.6 M GuHCl, 1 M urea, or 0.1% SDOC/2.4 mM n-LS/1 mM TCEP. With the exception of DigDeApr experiments (below), a protease:substrate ratio of 1:50 or 1:100 was used. With the exception of LysC, which was used for 72 hr at room temperature, all protease digestions were overnight at 37°C.

For CNBr+Trypsin digestion, quenched amine-amine crosslinking samples (above) were supplemented with 100% formic acid (FA) to 70% (final) followed by the addition of one crystal (~20–100 molar excess) of CNBr and overnight incubation at room temperature in the dark. After evaporation to dryness under vacuum, samples were redissolved in urea buffer (above), followed by 30 min incubation at 37°C in the dark. Samples were then diluted to 1 M urea with 50 mM TEAB (pH 8.5), and trypsin added to an estimated enzyme-to-substrate ratio of 1:100 followed by overnight incubation at 37°C. A fresh equivalent of trypsin (same amount) was then added, followed by a further 4 hrs digestion. Undigested material was precipitated by centrifugation at 14,000 g for 2 min followed by resuspension in 70% FA and re-digestion with CNBr and trypsin following the same method.

### DigDeApr

This was done following ref. [124] with modifications. Briefly, samples were digested with either trypsin alone (enzyme:substrate ratio of 1:2500) or Trypsin+AspN, Trypsin+GluC or AspN+GluC (1:1:2500). After overnight incubation at 37 °C, samples were centrifuged into a centrifugal concentrator (10kDa MWCO, Vivacon) at 2500 x g. After collection of flow through, the filter was washed by centrifugation at 2500 g with 8 M urea, 0.1 M TEAB pH 8.5 then with 2 M urea, 0.1 M TEAB pH 8.5 (Wash buffer) for 2 min at 2500 x g. Flow through and wash-throughs were combined. Using a new collection vial, urea buffer was added to the filter which was then inverted and spun for 2 min at 2500 x g. The process was repeated and the combined urea buffer washes were brought to 1 M urea with 0.1 M TEAB then treated again with the same protease combination at an enzyme:substrate ratio of 1:100 (for GluC digestion, samples were diluted to 0.5 M urea, 100 mM TEAB) overnight at 37 °C.

### C18-SCX

All cleaved samples were acidified with FA to 2% FA final then desalted as described [125] using stacked C18-SCX filters. After washing the filters, peptides were transluted from the C18 to the SCX phase using 80% CH3CN/0.1% FA (translution buffer). Peptides were eluted with 5% NH_4_OH/80% CH_3_CN (Buffer X) or with six steps of 20% CH_3_CN/0.5% FA containing ammonium acetate in the range 160–800 mM followed by a final step of Buffer X. Elutions were dried under vacuum then re-dissolved in 0.1% FA in water for MS.

### nanoLC-MS/MS

nanoLC-MS/MS was performed using an LTQ Velos Pro Orbitrap mass spectrometer with Easy-nLC 1000 (ThermoFisher). 2 microL injections were followed by a segmented LC gradient (solvent A = 0.1% FA in water, solvent B = 0.1% FA in CH_3_CN), progressing from 0 to 10% B over 10 min then to 35% B over 230 min. Some runs used a straight gradient of 0–35% B over 135 min. Precursor spectra were acquired in FT mode at a resolution of 100,000 (centroid) in the range 200–2000 Th. For isotopic pairs with 12 Da mass split (DSS crosslinker), the top 3 most intense ions were selected for HCD activation (above a precursor signal threshold of 150) on the basis of isotopic pairs with m/z spacing of either 4.02524, 3.01893 or 2.41515 (representing +3 to +5 charge-states), and intensity ratio better than 2:1. For a 6 Da mass split (DSG crosslinker), m/z deltas for isotopic pair selection were 2.01456, 1.51092 or 1.20874. For a 4 Da mass split (BS3 crosslinker), m/z deltas were 1.34156 or 1.00616. Both isotopic partners were fragmented. HCD activation used a normalized collision energy (NCE) of 45, activation time of 0.1 mSec and an isolation width of 2 m/z. MS2 spectra were acquired in FT mode with a resolution of 7500 (centroid). The dynamic exclusion list size was 500, exclusion duration was 60 sec, repeat duration was 30 sec and the repeat count was 2, with early expiration enabled. Charge state screening was enabled, with rejection of 1+ and 2+ and unassigned charge states.

Data acquired for xQuest were activated in IT-CID mode instead of HCD. Here, NCE was 35, activation Q = 0.25 and activation time was 10 mSec. For non-isotopic crosslinkers, the 10 most intense ions in each precursor spectrum were subjected to HCD fragmentation, as above, if above a minimum signal threshold of 250 (or 2000 in some early experiments).

### Bioinformatics

Protein names used throughout this report follow entry names in the UniProtKB Vaccinia WR reference proteome minus the species identifier suffix. They are comprehensively cross-referenced to other naming schemes in Table S1 of ref. [5]. Instrument raw files were converted to mgf, mzXML or mzML using MSConvert by ProteoWizard. Using the resulting data, XL were identified using the following XL search engines: Protein Prospector [126], pLINK [127], xQuest (in combination with xProphet) [122], Kojak [128] (in combination with ‘Percolator’ [129–131]), ECL [132] and ECL2 [133], as follows:

Protein Prospector: Instrument raw data files were converted to mgf format then uploaded to Protein Prospector via the UCSF online server using parameters outlined in Table I in S1 Text. Non-standard, PEGylated bis(sulfosuccinimidyl)suberatecrosslinkers (BSPEG5 and BSPEG9) were imputed as user defined parameters. The results file from each run was generated using the program’s Search Compare function. Results were sorted by ascending expectation value and “Report type” was set to “crosslinked peptides”. ‘SD-E’ = ScoreDiff–log_10_(Exp2) ([126], Robert Chalkley, Personal communication) where ScoreDiff is the difference in score between the top- and second-ranked peptide 1 in the search output for a crosslinked pair, Exp2 is the score for peptide 2.

pLink: pLink was downloaded from pFind Studio. A parameter file was configured for each experiment and a folder created, containing mgf files pertaining to that experiment along with the search DB. The ‘pLINK.ini’ configuration file was modified for each experiment to include the path to the mgf and search DB and search parameters (Table I in S1 Text). The enzyme.ini and xlink.ini files were modified for any non-standard cleavage specificities/combinations and crosslinkers, respectively. Results files for loop linked and mono linked peptides were generate using “non-interexport” and “drawpsm”, respectively. pLink was run through the flow.exe application.

xQuest: The xQuest VMware package was installed on a Windows PC. Directories were created following instructions provided with xQuest. Search parameters are given in Table I in S1 Text. Files “Xmm.def” and “xquest.def” were modified for the relevant crosslinker isotopic mass, shift and ion charge states. A text file was created containing the mzML file name and parameter files for xProphet. xQuest, then xProphet, were run from the command line. Results were viewed on the xQuest webserver then downloaded. Values reported by xProphet as "FDR" may be Percolator-derived q-values.

Kojak: Kojak and Percolator [134] were installed and run in Linux from the command line. Folders were created for mzML formatted data and search results. The program’s configuration file was modified to contain all relevant crosslinkers and the paths to individual data files. Parameters are outlined in Table I in S1 Text. Digestion specificity rules were based on the parameters provided.

ECL/ECL2: ECL and ECL2 were installed on a Java-capable Windows PC and run from the command line. The program’s parameter file was modified to contain search parameters given in Table I in S1 Text. Crosslinker masses were entered manually.

Percolator: For Kojak and ECL, FDR was converted to a q-value using the program ‘Percolator’ [134], run from the Linux command line. For Kojak, Percolator input comprised “inter”, “intra”, and “loop” search output files. q-value can be regarded as the expected proportion of false positives among all features as or more extreme than the observed one [132, 135] or, alternatively, the minimal FDR threshold at which a given peptide-spectral match is accepted [130, 131].

Data assembly: Using in-house code, XL search engine/Percolator/xProphet outputs corresponding to various nanoLC-MS/MS runs in various experiments were parsed in their native formats, accepting individual XL to a single unified dataset according to dual score thresholds for each program including in-house-calculated FDR for Protein Prospector (see above and Table 2). The resulting dataset was then sorted by ascending exp_Mr. Groups (blocks) of masses matching to within 25 ppm were annealed, then each block that contained multiple accession/PeptideSeq/ProteinPos was sorted and divided into distinct sub-blocks of ions that were tagged with a common ‘ambig code’ (representing sub-blocks having functionally isomeric mass but had been assigned, by XL search engines, distinct apparent identities). The resulting ‘mature blocks’ each represented a unique combination of exp_Mr and accession/PeptideSeq/ProteinPos.

This dataset was reformatted/collapsed into a matrix with one row per mature block, and one column for each nanoLC-MS/MS run in the project. The matrix was filled with XL search engine identifiers to indicate all engines identifying a specific mature block member in a specific nanoLC-MS/MS run and the number of times identified. Each row was assigned a DFscore as the sum of search engine identifiers/times identified by that engine that had been assigned to the row. Groups of mature blocks sharing a common ambig code were likelihood-scored against one another as follows: If they all represented intra-protein XL or all represented inter-protein XL, then the ambigscore assigned to those mature blocks was a simple proportion of its DFscore/∑(DFscores for all blocks sharing a common ambig code). If they were a mixture of intra-protein and inter-protein, then intra-protein mature-block(s) were scored 1.0 and inter-protein mature-block(s) 0 (assuming the intra-protein XL to be correct by default). If the ambiguity was simply in choice between multiple lysines within an otherwise identical peptide, both choices were scored 1.0 since both reflect the same approximate position within the same protein partners. Finally, for every specific position in a specific protein represented by multiple rows in the matrix: If the intra-protein XL were discovered by multiple engines and the inter-protein XL were discovered by one only, the latter were annotated as “filterable”. The resulting annotated matrix was written to an Excel worksheet then copied to a second sheet which was re-sorted by protein position then accession.

A ‘discard matrix’ (comparable in structure to the above, ‘passing’ matrix) was generated representing all XL ions in the above assembly that passed threshold1 but were rejected after failing threshold2. Each row of the ‘discard’ matrix was annotated with: (a) DFscore; (b) whether the XL (Accession1/Accession2/ProteinPos1/ProteinPos2) was also present in the passing matrix (above; this criterion being denoted ‘also’ in the following discussion) and (c) ‘biological rationality’ (‘BR’) based on six groups of functionally-related virion proteins (Table 3), annotating”Y”, if the two crosslinked proteins were in same BR group, and “N” if one was from the ‘membrane’ group and the other from the ‘transcription’ group.

Networks and sub-networks for individual accessions or groups of accessions were rendered using CrosslinkViewer [136]. Using in-house code, rows in the passing matrix (above) were picked provided either one or both crosslinked proteins did not match accessions within a user-definable excluded-accession group. ‘Filterable’ rows of the matrix (above) were excluded. The list of picked rows was supplemented with those from the ‘discard’ matrix (above) if DFscore > 1 or ‘also’ = “Y” or BR = “Y”. Rows with common Accession1/Accession2/ProteinPos1/ProteinPos2 were then collapsed summing DFscores, and the resulting dataset reformatted for input to CrosslinkViewer. The resulting DFscores were rendered. If 100% of matrix rows for a given Accession1/Accession2/ProteinPos1/ProteinPos2 had been flagged as ambig (above), then the XL was flagged to be rendered with a broken line. Protein monolinks were ignored in all data operations.

Domain prediction: TM regions were predicted using program TMHMM [68, 95]. Domain boundaries were predicted using DomPred [137, 138]. Output traces show endpoint density profiles for PSI-BLAST alignments generated between a query sequence and a database in which all sequence fragments had been removed.

ROC analysis of the global crosslinking dataset: Each of the 81 proteins in the dataset for which crosslinked partner proteins were found, was flagged according to membership of one of two ‘biological rationality’ groups in Table 3 (‘Membrane’ and ‘Transcription’), and total number of distinct crosslinking partner proteins was printed alongside. In each of two replicates of this listing was printed the # of partners belonging specifically to one of the two groups and the list was sorted (descending) according to proportion of total partners belonging to the specific group. After incrementing four number series at each row in the list that contained either: a membrane protein, not(a membrane protein), a transcription protein and not(a transcription protein), then proportionating each series to a scale from 0 to 1, ROC curves were drawn based on the proportionated values. In ROC space, points above and below the line of no-discrimination (diagonal) represent positive (better than random) and negative correlation, respectively such that a curve representing perfect positive correlation would ascend vertically from (x,y) = (0,0) to (0,1) then travel horizontally to (1,1). Perfect negative correlation would yield the converse curve: (0,0) to (1,0) to (1,1).

Inter-protein XL partitioning analysis: For each XL ion in the global XL dataset (each row of S1A Table) representing an inter-protein XL, DFscores from each experiment (column) in which the ion was detected were binned according to whether sample pre-treatment included or excluded NP40 or TCEP (-NP40, +NP40, -TCEP or +TCEP). The resulting tetra-bin DFscore values for individual ions were accumulated on a per-accession basis, according to the accession on each side of the crosslink. The accumulated four DFscore values for each accession were then converted to a proportion of the summed DFscore across the four bins (‘POSD’) for that accession, and the resulting POSD values were finally normalized to the mean POSD per accession for each of the four pre-treatment conditions. See legend to S3 Fig for further details.

### Distance measurement

Distance analysis of the form shown in ref. [29]) was generated using ‘TopoLink’ [38] installed on a computer cluster and run from the command line, in combination with 14 relevant pdb files (Table B in S1 Text). Before calculating Euclidean and SAS distances for each experimental XL, “inputfile.inp” was modified to include the crosslinker type, maximum linker distance and reactive residues. All lys-lys Euclidean and SAS distances were also calculated within the 14 structures, setting maximum linker distance to 100 Å. Each of the resulting four distance datasets, in spreadsheet format, was binned for display as a histogram. The mean and standard deviation (SD) from each “all lys-lys distances” histogram informed a normal (Gaussian) curve overlay. For each “experimental XL distances” histogram, Ln(mean) and ln(SD) informed a log-normal curve overlay. “Experimental XL distances” and “all lys-lys distances” datasets were compared to one another via the Kolmogorov Smirnov test, run using the Excel plugin XLSTAT (https://www.xlstat.com/en/).

## Supporting information

S1 Fig Crosslinked partners (circles whose areas correspond to chain length) for each protein (rectangle whose length corresponds to chain length) in the crosslink dataset, one protein per page. Above the target protein are partners with a single XL, and below are those with two or more distinct crosslinks. Green fill: Proteins in the transcriptosome group (Table 3). Magenta fill: Proteins in the ‘7PC’ group. TM proteins—yellow, red and cyan fill: ‘Outside’, TM and ‘inside’ domains, respectively (see legend to Fig 8). Green lines: Inter-protein XL. Numbers (red font) in gray squares with black border: DFscore (crosslinks showing no DFscore had a DFscore of 1). Dashed crosslinks are ‘ambiguous’, ie. they are members of a group of distinct crosslinked peptide pairs whose experimental ion masses differed by less than the annealing mass tolerance (25 ppm, Materials & methods). Although the isomeric group members likely represent distinct crosslinks (with distinct sequencing ions), they are flagged here simply to note that they would not be discriminatable on the basis of parental ion mass alone. Some dotted (‘ambig’) XL overlay non-dotted ones that are inapparent. White numbers in black boxes: Ambiguity scores (see Materials & methods). For proteins A9, A46, D2 and SODL, for which no XL partners were found, intra-protein XL are shown.
(PDF)

S2 Fig (**a**) Intra-protein crosslinks within all TM proteins. Yellow, red, cyan fill: ‘Outside’, TM, ‘Inside’ domains, respectively (see legend to Fig 8). Mauve loops (above protein): Crosslinked peptides from the same protein sequence. Although depicted as intra-molecular (‘intra-protein’) XL, formally, any of them could, instead, span homomultimer subunits. Red loops (beneath protein): Identical crosslinking site from the same protein for both members of the crosslinked peptide pair (a *bona fide* indicator of homomultimerization). Vaccinia ATI protein is included in the TM protein group due to its predicted possession of a TM domain with 80% probability (albeit this was a lower probability than for the other TM proteins, see below). With few exceptions, intra-protein crosslinks followed the discrete domains predicted by the program TMHMM [68], ie. did not show membrane-spanning ‘Inside’-to-’Outside’ XL. Proteins containing apparent exceptions to this are indicated with a red star (*) at the C-terminus (right-hand column of proteins). Although lipid bilayers (~50 Å in thickness [141]) are substantially beyond the 11 Å span of crosslinkers such as DSS, a number of experiments involved brief NP40+TCEP pre-treatment with the likelihood of liberating proteins from the virion envelope. Nonetheless, TM proteins can retain rigidity and structural integrity in their (bundled) bilayer-spanning portions even in the absence of a lipid barrier [142, 143], and TM domains are typically impoverished in charged residues such as lysines, which comprised the crosslinking targets in most of our experiments. The paucity of XL spanning predicted TM domains provided additional validation for the XL dataset as a whole. No intra-protein XL were detected for TM proteins F14.5 and I5, which are therefore not depicted. (**b**) TMHMM prediction of TM domain(s) in Vaccinia protein ATI. In contrast to all other TM proteins, whose TM domain prediction posterior probability (red) approached 1.0 (100%), for ATI it was 0.8 with two additional candidate TM domains scoring 0.2. (**c**) ATI shown with the two minor TM domains from panel B colored pink. Speculatively, these may combine into a single (‘slippery’) membrane-spanning domain with higher probability than either one alone. If ATI is a membrane double-spanning protein, its N- and C-termini would be on the same side of the membrane. These are arbitrarily colored yellow (‘Outside’ color) but could equally be both inside.
(PDF)

S3 Fig Crosslink partitioning analysis (Materials & methods) for inter-protein XL. Briefly, Y infers the number of XL detects for an accession in the presence (+) or absence (-) of virion pre-treatment reagents NP40 and TCEP, as a proportion of total XL detects for the accession, and after each of the four resulting values were then normalized for the different number of XL detects per accession for each treatment condition. Y > 1 and Y < 1 infer that the stated pre-treatment tends to stimulate or suppress crosslinking, respectively, with respect to an average protein in the virion crosslinkome. Actual pre-treatment conditions (Fig 1) were deconstructed to yield the bars shown: The -NP40/-TCEP (‘None’) condition was binned as both–NP40 and–TCEP, the +NP40 alone condition was binned as both +NP40 and–TCEP, and the +NP40/+TCEP condition was binned as both +NP40 and +TCEP. Values +TCEP alone are therefore inferred. Upper-left, upper-right and lower panels: Key structural, membrane and ‘DNA group’ proteins, respectively.
(PDF)

S1 Table A, B. 4609 confidently-identified unique-mass crosslink ions for the project (rows) after dual-thresholding and annealing of common masses (Materials & methods). Ions are matrixed against crosslinking samples (columns H to BJ). Other than SCX peptide fractionations, in which a sample represents the combined search results from multiple nLC-MS/MS runs, each column represents the results of a single nLC-MS/MS run. Column BK: DFscore. Columns BL, BM: Ambig score and correlating rows considered mutually ambiguous XL, respectively. Column A yellow highlights: Inter-protein XL. Column headers are colored by virion pre-treatment (Blue: Intact virion; red: NP40 only; Green:NP40/TCEP). (**A**) Crosslink dataset sorted by ion mass Columns BN–BS: Search engine correlations. (**B**) Crosslink dataset sorted by Accession/Position. Column BN: ‘Filterable’ ion identities (Materials & methods). Colored highlights (columns F, G and corresponding samples): Crosslink positions representing neither lysine nor full-length protein N-terminus. The great majority (“2”) represent the N-terminus of the demethionylated protein. Others represent either an other-than-amine-specific crosslinker, or a lysine in a different Vaccinia WR sequence. **C**. All crosslinked inter-protein pairs in the dataset, ranked by descending inter-protein DFscore (summed DFscores for all crosslink ions for the protein pair; column C). Column D shows ‘biological rationality’ (see main manuscript). Non-random coverage of inter-protein crosslinking space is apparent, with inter-protein crosslinking space dominated by p4a-p4b XL (inter-protein DFscore = 699).
(XLSB)

S1 Text Table A. Proteins in the XL search database in relation to their virion packaging status, and for which XL were not detected. Table B. Partial and complete X-ray crystallographic structures covering the crosslinked portions of proteins in the XL search database. Table C. Numbers of crosslinking ions detected for the three predicted fragments of protein p4a and two predicted fragments of protein p4b. Table D. EFC interactions noted previously and in the current study. Table E. DFscoring inter-protein XL in the dataset, ordered by descending DFscore. Table F. Matrix of all detected interactions between transcriptosome proteins (listed on row 1) and membrane (TM plus membrane-associated) proteins (listed in column 1). Table G. Matrix of all detected transcriptosome-transcriptosome protein interactions. Table H. Crosslinked peptides in the dataset spanning AG| sites known to be cleaved during virion maturation. Table I. Parameters used with crosslink search engines.
(PDF)
