# Supplementary material for: The Vaccinia virion: Filling the gap between atomic and ultrastructure
Source: PLoS Pathog. 2019 Jan 7;15(1):e1007508. doi: 10.1371/journal.ppat.1007508 (PMC6336343; doi:10.1371/journal.ppat.1007508)
Supplement: S1 Fig — Above the target protein are partners with a single XL, and below are those with two or more distinct crosslinks. Green fill: Proteins in the transcriptosome group (Table 3). Magenta fill: Proteins in the ‘7PC’ group. TM proteins—yellow, red and cyan fill: ‘Outside’, TM and ‘inside’ domains, respectively (see legend to Fig 8). Green lines: Inter-protein XL. Numbers (red font) in gray squares with black border: DFscore (crosslinks showing no DFscore had a DFscore of 1). Dashed crosslinks are ‘ambiguous’, ie. they are members of a group of distinct crosslinked peptide pairs whose experimental ion masses differed by less than the annealing mass tolerance (25 ppm, Materials & methods). Although the isomeric group members likely represent distinct crosslinks (with distinct sequencing ions), they are flagged here simply to note that they would not be discriminatable on the basis of parental ion mass alone. Some dotted (‘ambig’) XL overlay non-dotted ones that are inapparent. White numbers in black boxes: Ambiguity scores (see Materials & methods). For proteins A9, A46, D2 and SODL, for which no XL partners were found, intra-protein XL are shown. (PDF) [file ppat.1007508.s001.pdf]

7PC

A15, A30 , D2, D3, G7, J1, VPK2

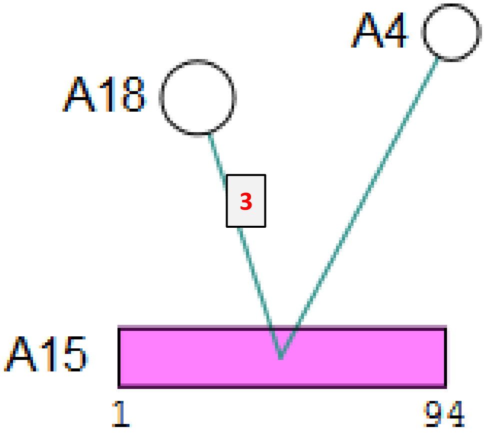
A18
A4
3
A15
1
94

A30-49/P4A-876: 0.080

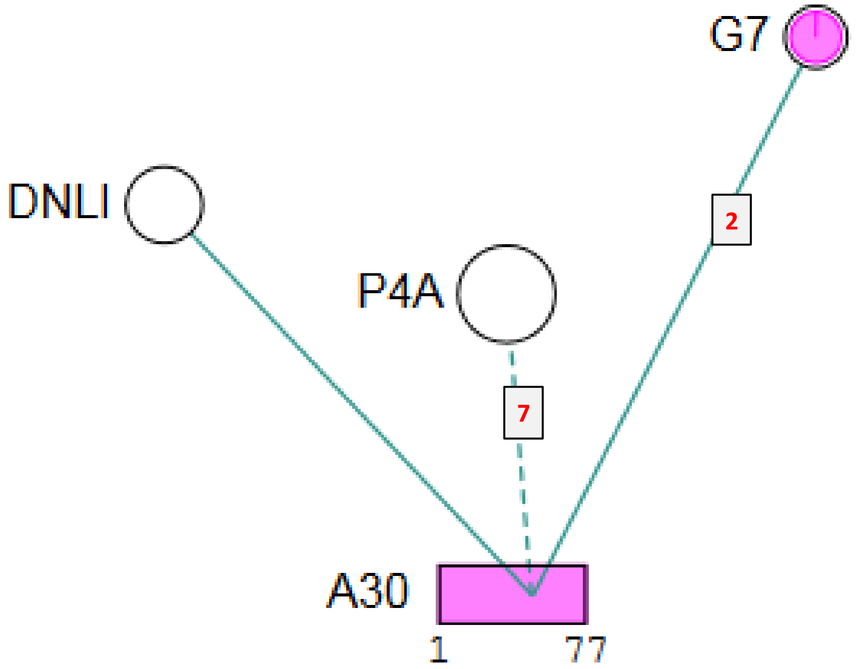

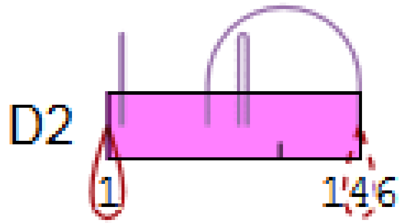
D2
1
146

D3-1/MCE-8: 0

D3-7/RP147-54: 0

D3-232/P4B-189: 0

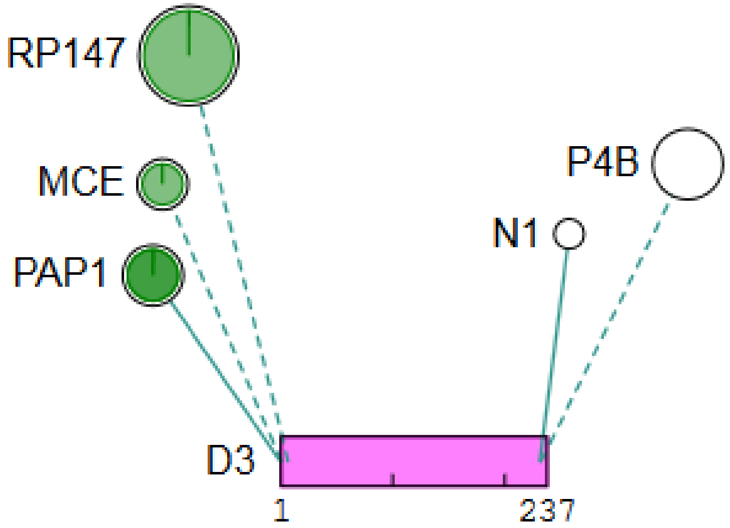

G7-1/RP147-75: 0

G7-345/E3-165: 0

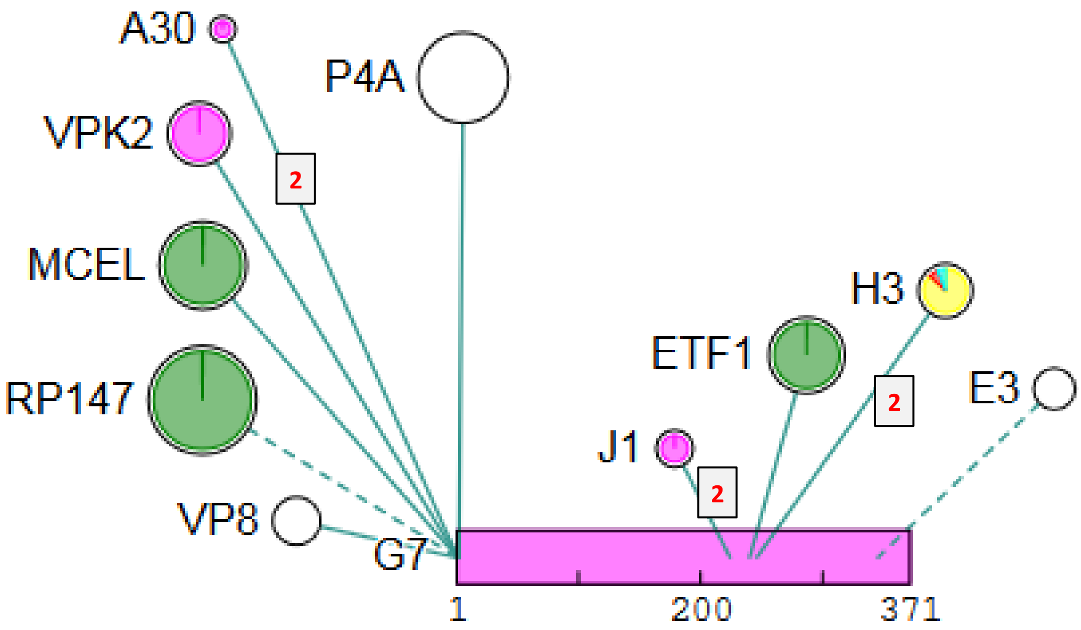

J1-2/A16-206: 0

J1-74/F17-74: 0

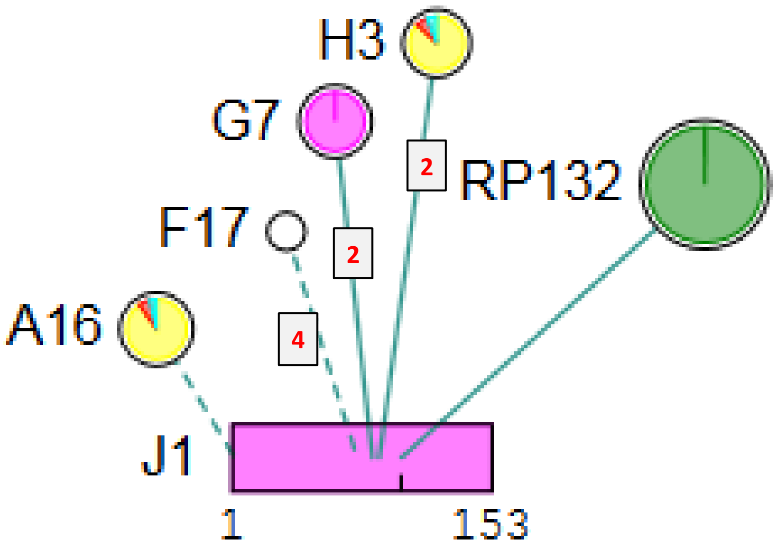

VPK2-1/DNLI-303: 0.667

VPK2-205/A6-352: 0.500

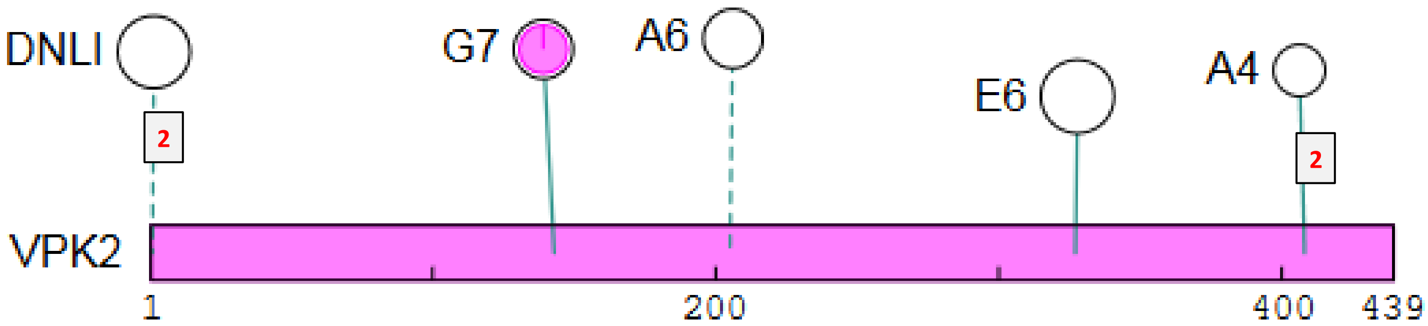

EFC/EFC-associated

A16, A21, A28, F9, G3, G9, H2, J5, L1, L5, O3

A16-22/RAP94-516: 0

A16-116/A27-37: 0

A16-133/P4A-754: 0

A16-149/I7-126: 0

A16-153/RP147-493: 0

A16-194/A4-33: 1.000

A16-194/A4-42: 1.000

A16-206/J1-2: 0

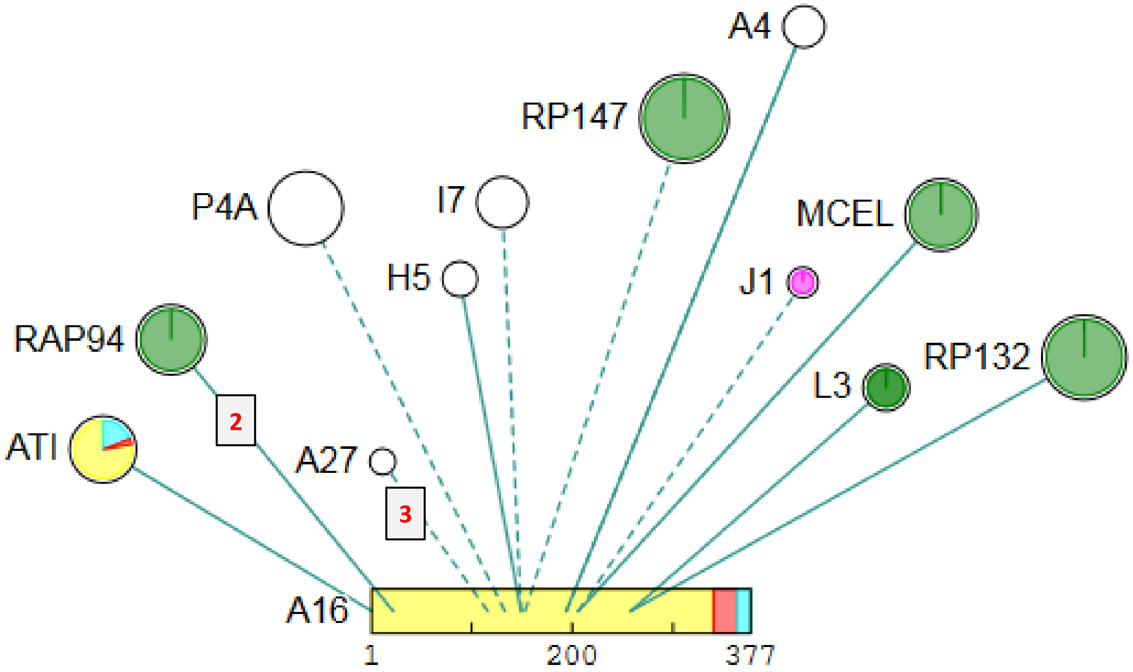

CAHH-100/A21-63: 0

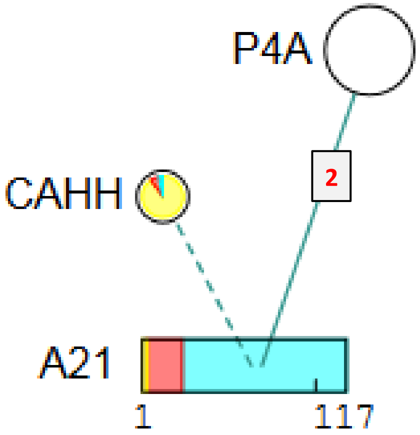

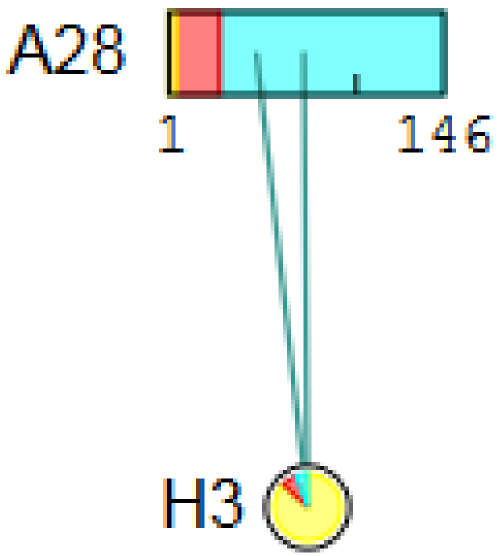
A28
1
146
H3

F9-0/A12-75: 0

F9-203/RP147-148: 0.111

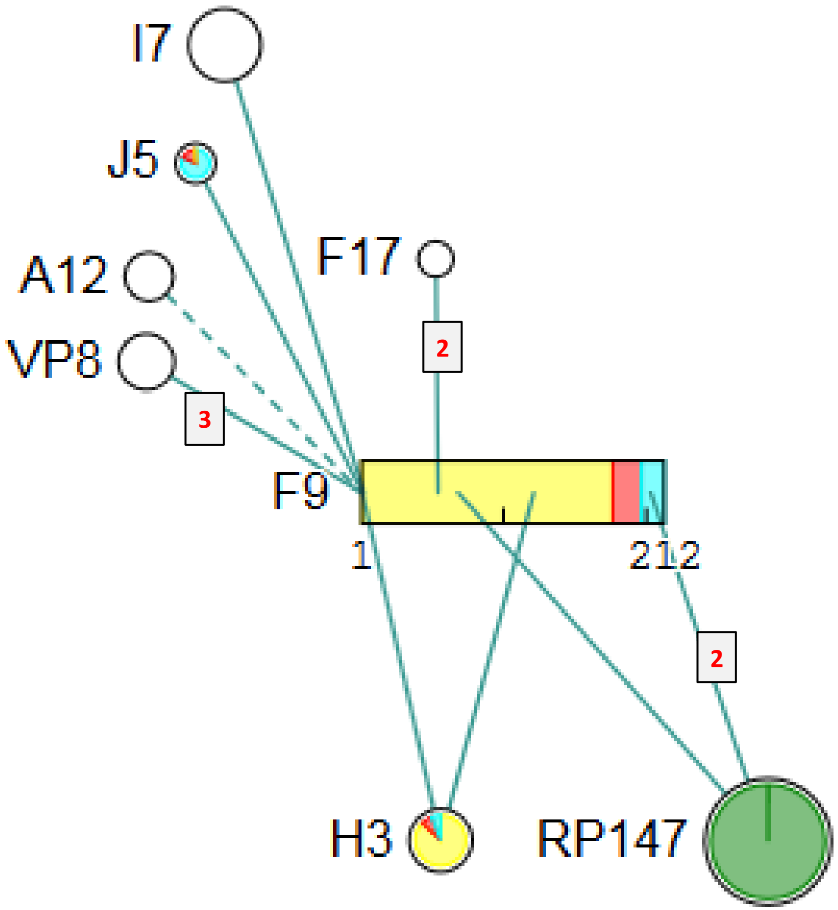

G3-88/P4A-876: 0.167

G3-111/ATI-38: 0.250

G3-111/L5-109: 0.750

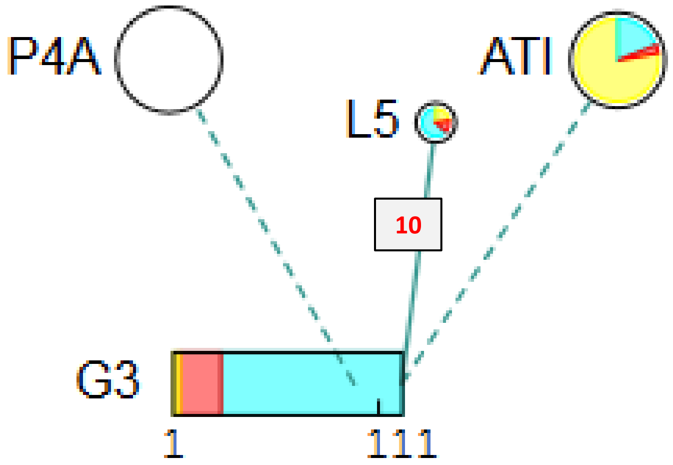

G9-52/ATI-512: 0

G9-55/RP147-956: 0.500

G9-57/RP22-1: 0

G9-58/E6-278: 0.143

G9-58/MCE-226: 0.857

G9-58/VPK1-121: 0

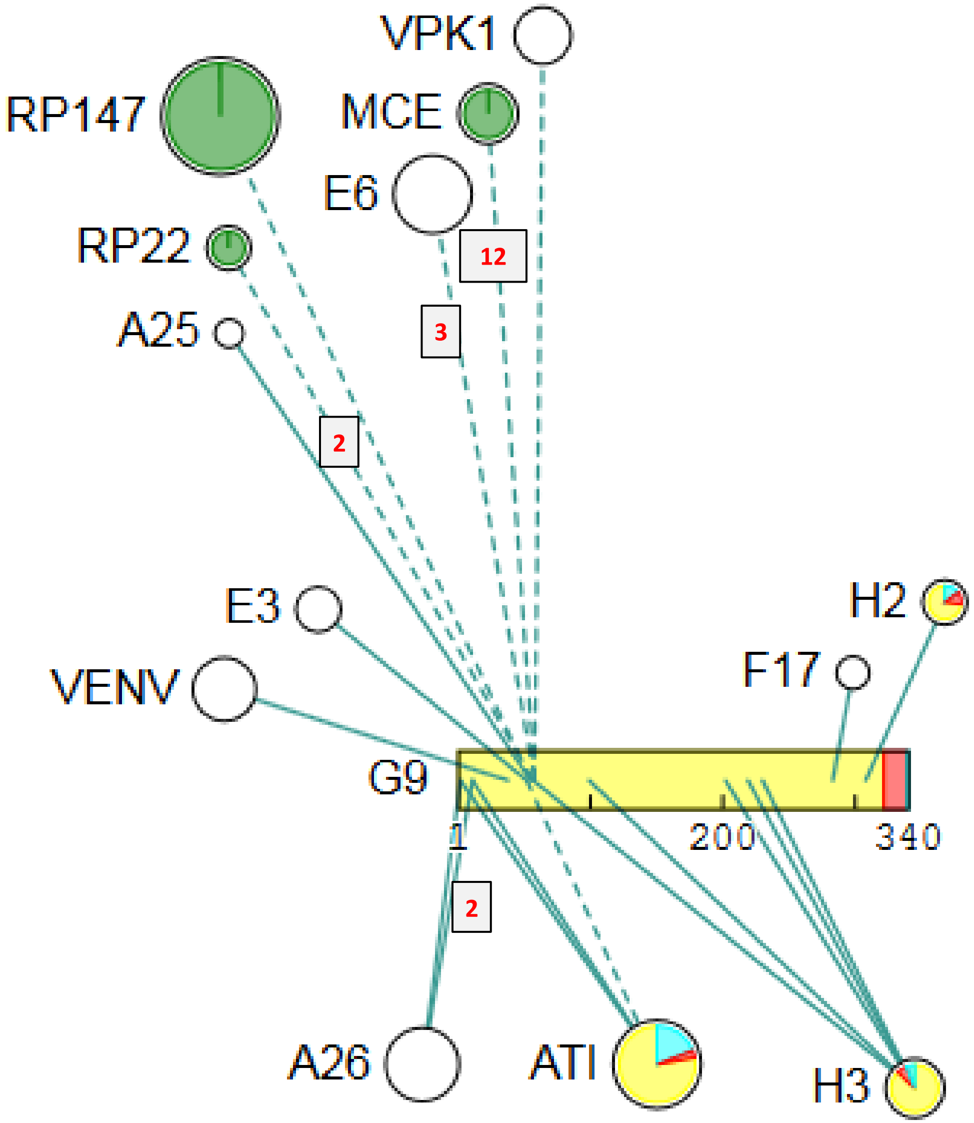

H2-155/ATI-175: 0.500

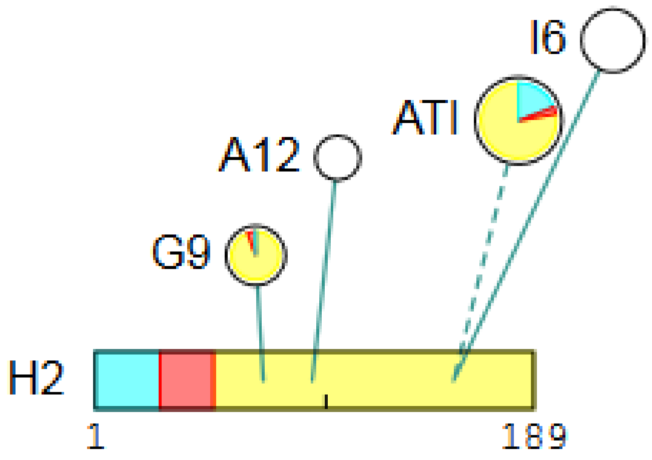

J5-82/ETF2-279: 0.500

J5-92/P4A-736: 0

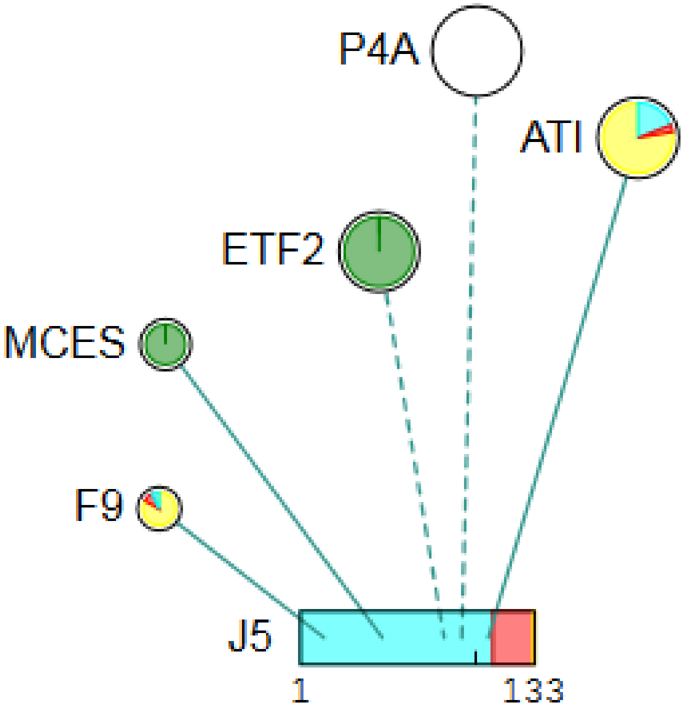

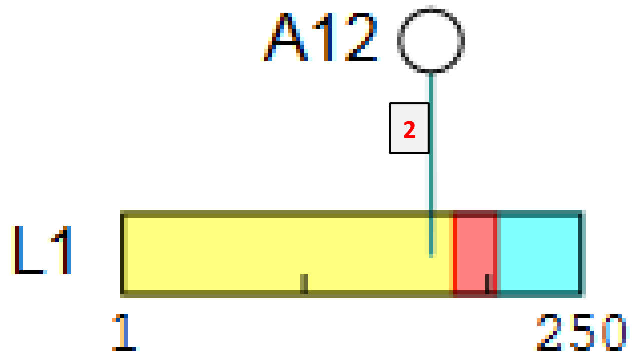
A12
2
L1
1
250

G3-111/L5-109: 0.750

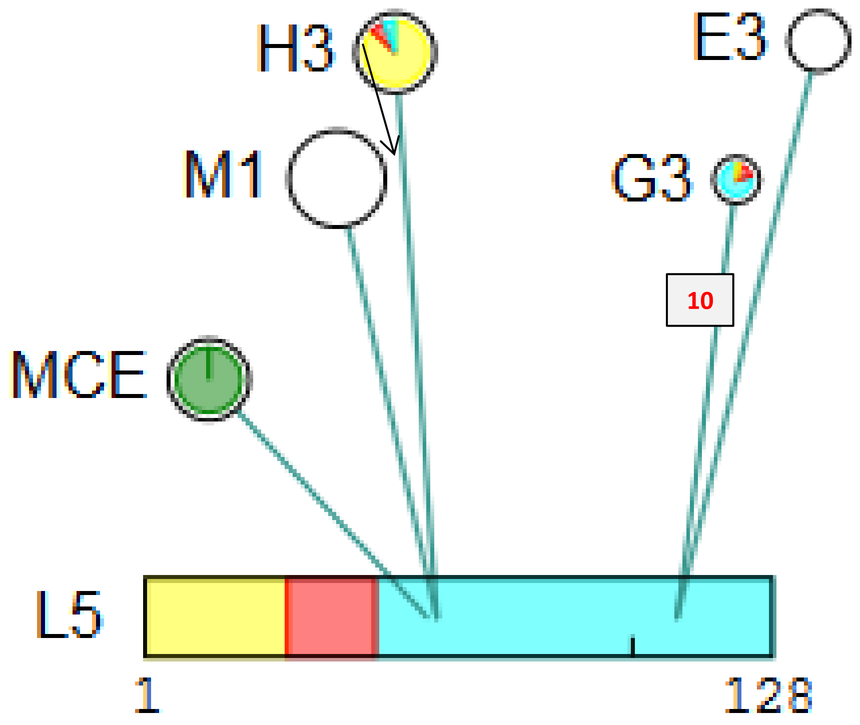

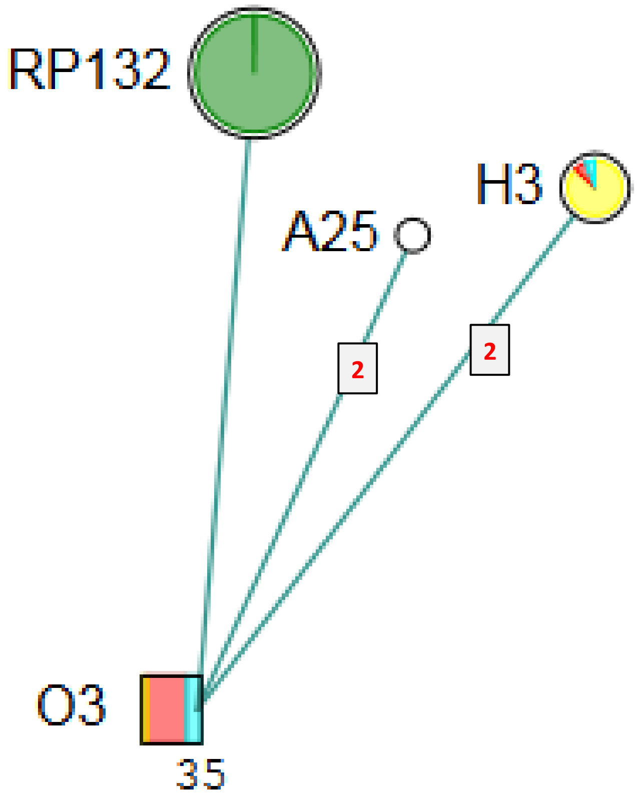

RP132
H3
A25
2
2
O3
35

Other transmembrane proteins

A9, A13, A17, ATI, CAHH, E8, F14.5, H3, I5

(Undetected: A14, I2)

No external XL.

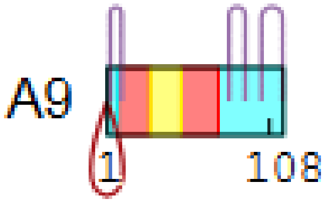

A9

```
# WEBSEQUENCE Length: 108
# WEBSEQUENCE Number of predicted TMHs:  2
# WEBSEQUENCE Exp number of AAs in TMHs: 38.70797
# WEBSEQUENCE Exp number, first 60 AAs:  30.70481
# WEBSEQUENCE Total prob of N-in:        0.81034
# WEBSEQUENCE POSSIBLE N-term signal sequence
WEBSEQUENCE     TMHMM2.0        inside       1     6
WEBSEQUENCE     TMHMM2.0        TMhelix      7    26
WEBSEQUENCE     TMHMM2.0        outside     27    45
WEBSEQUENCE     TMHMM2.0        TMhelix     46    68
WEBSEQUENCE     TMHMM2.0        inside      69   108
```

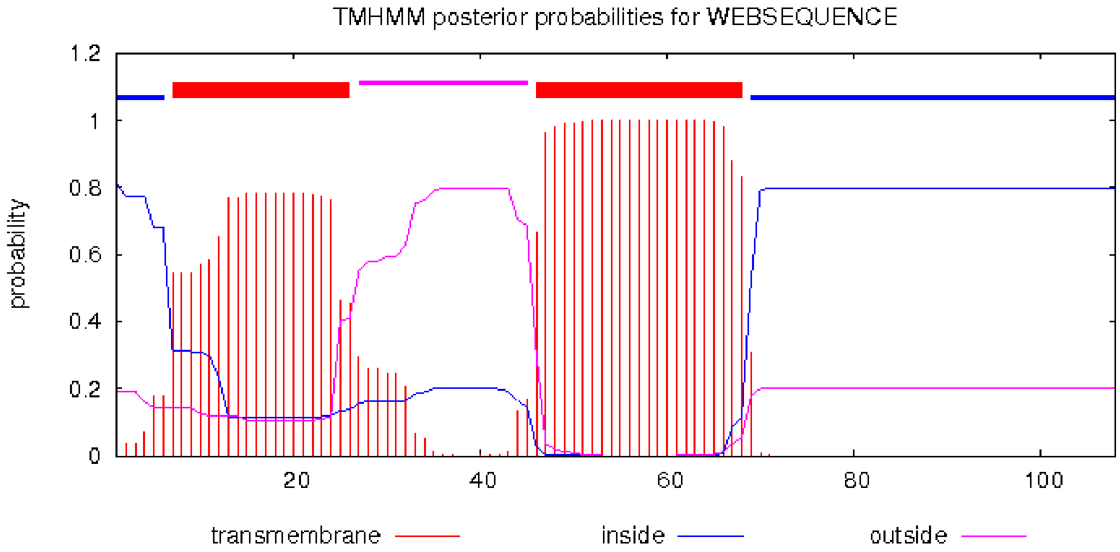

A13-49/E5-67: 0

A13-67/I7-90: 0

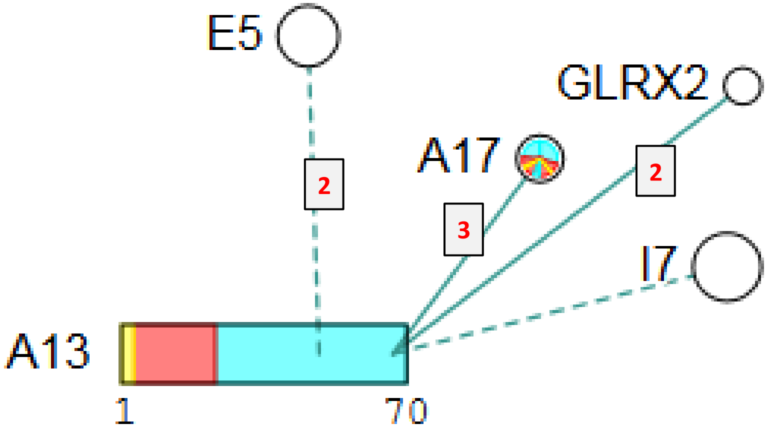

A17-36/H3-224: 0

A17-36/A27-98: 0

A17-180/F8-1: 0

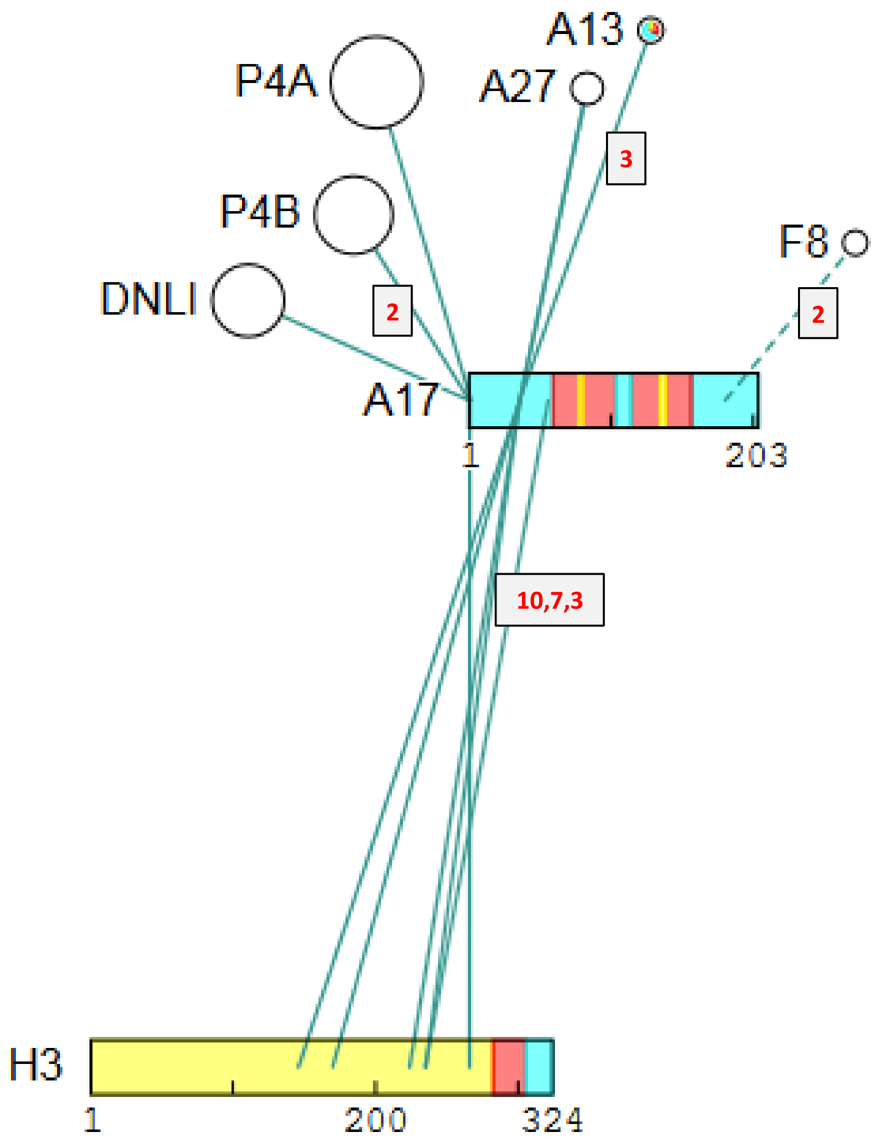

As prior slide, showing intra-protein also

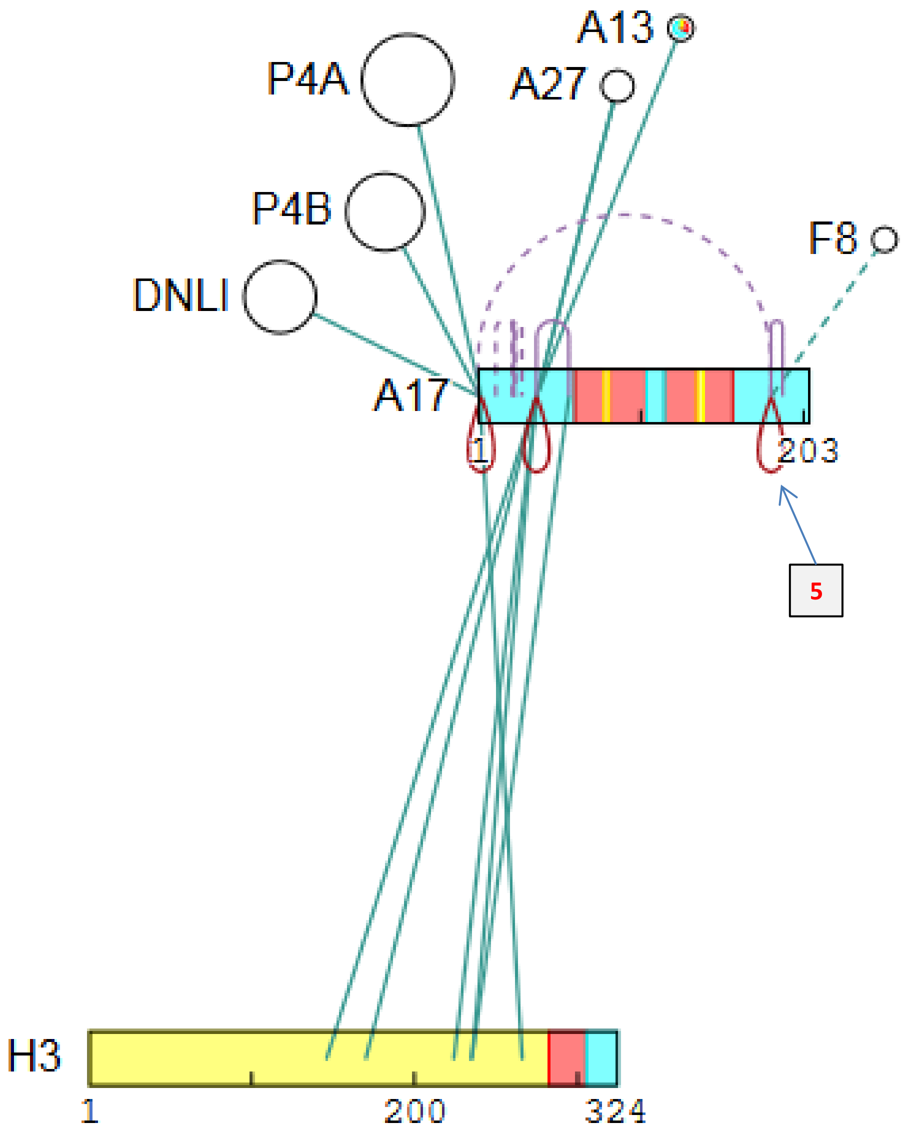

ATI-38/G3-111: 0.250

ATI-61/H3-147: 0.500

ATI-175/H2-155: 0.500

ATI-512/G9-52: 0

ATI-572/A12-88: 0

ATI-572/P4A-876: 0

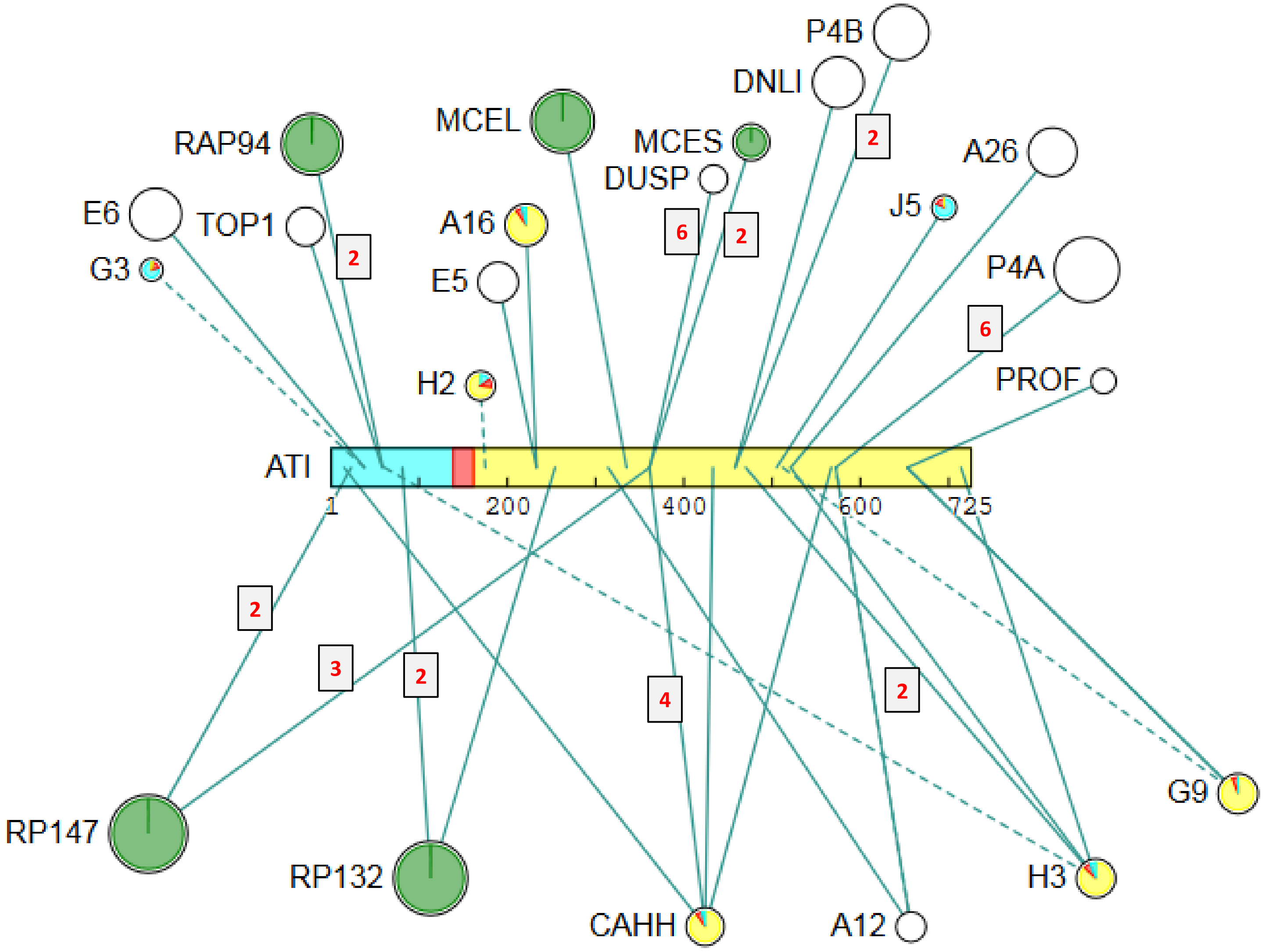

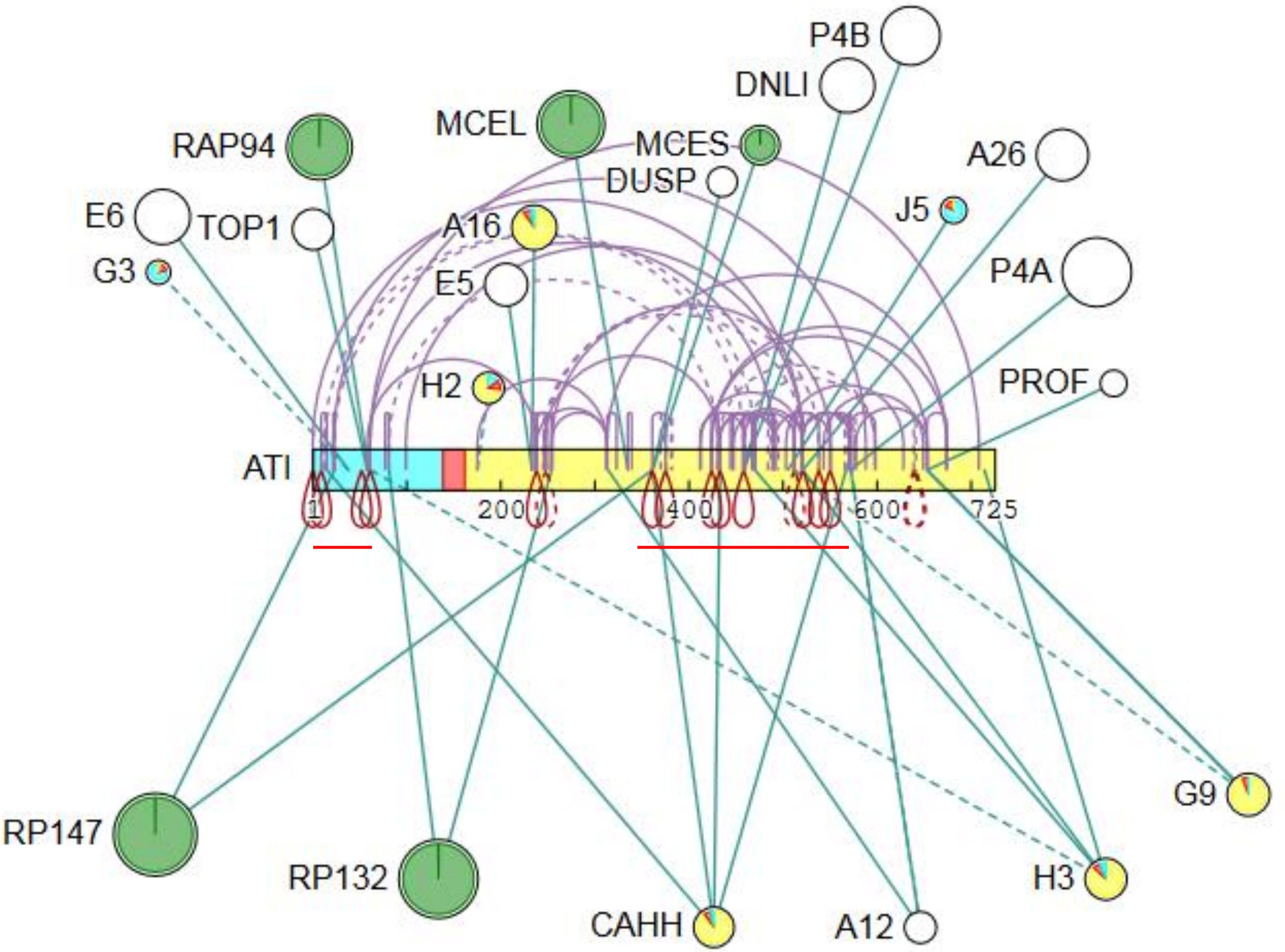

Horizontal red lines: Two dense regions of high-scoring intra-links = self-association?

CAHH-14/I7-146: 0

CAHH-100/A21-63: 0

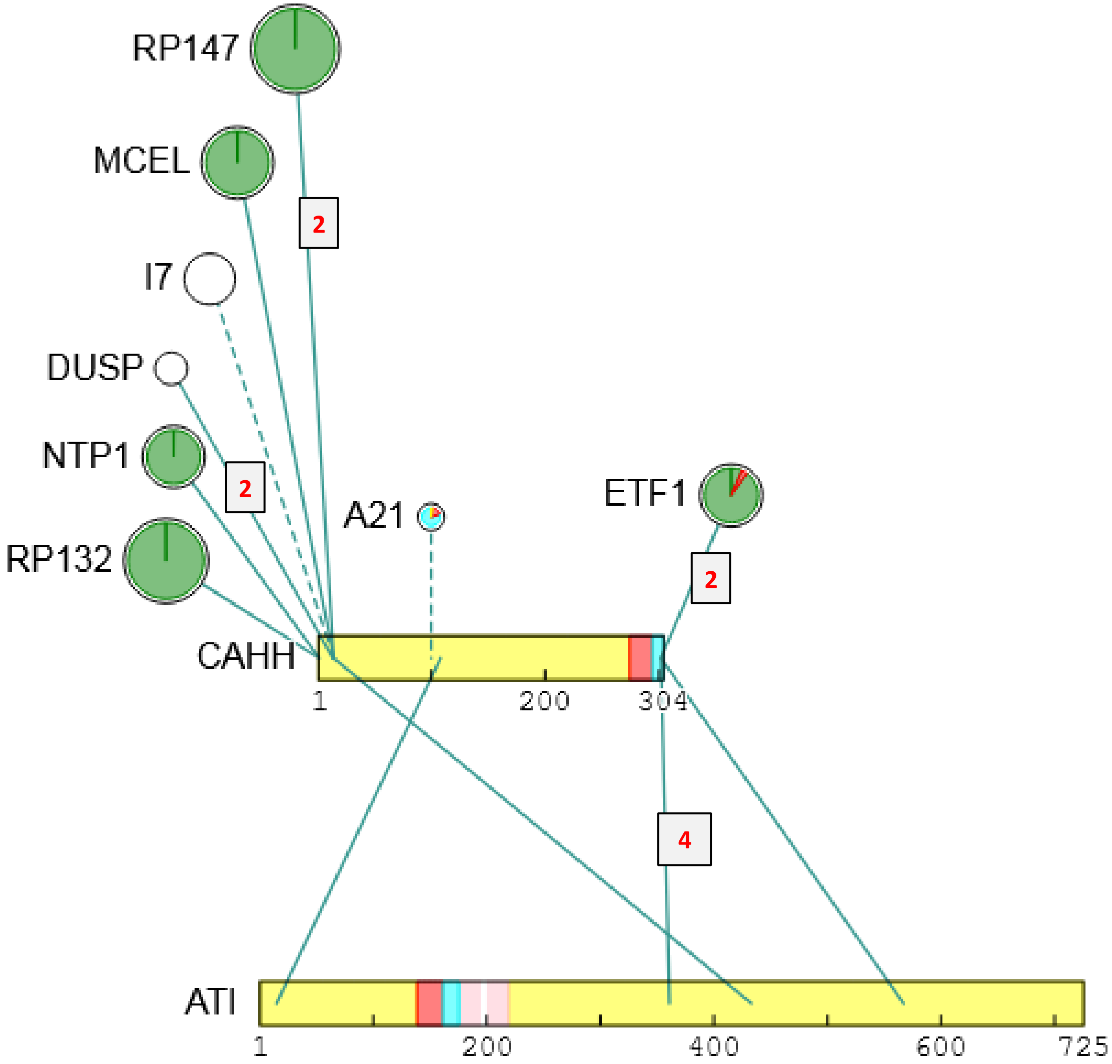

E8 Griffiths topology (Residues 1 – 124 argued to be 'inside'):
Doglio et al. (2002) suggests N-term 120 aa is on the cytoplasmic side due to antibody reactivity. aa144-237 would then be on non-cytoplasmic side.

E8 may loop around so that both inside (cyan) domains => transcriptosome

E8-1/A12-167: 0

E8-79/ETF1-229: 0

E8-201/I1-178: 1.000

E8-267/E6-552: 0

E8-267/RP147-603: 0

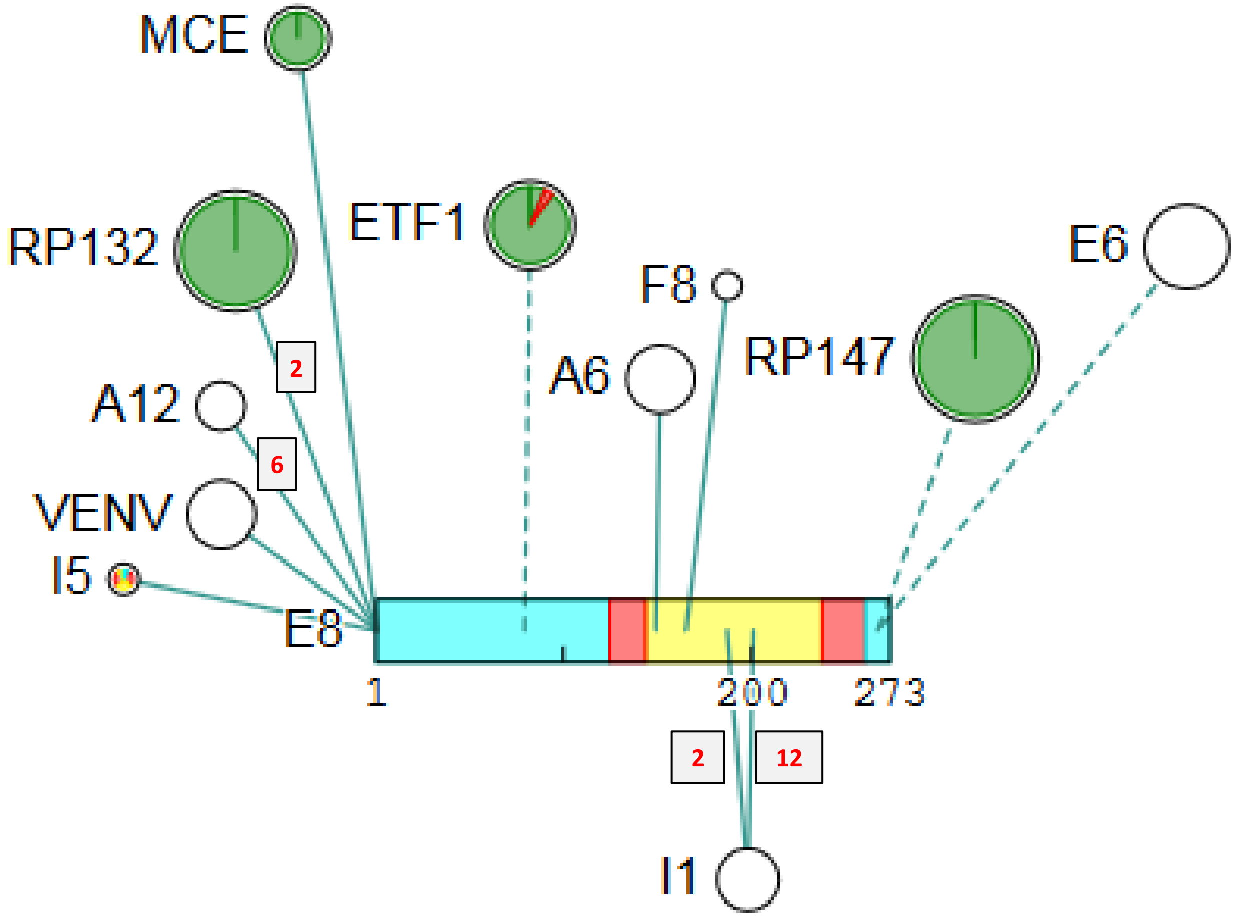

E8 TMHMM topology (Residues 1 – 124 argued to be 'outside'):
TMHMM suggests with 55% probability 'N-outside'
aa144-237 would then be on cytoplasmic (cyan) side.

Here, E8 may loop around so that both outside (yellow) domains => transcriptosome.

E8-1/A12-167: 0

E8-79/ETF1-229: 0

E8-201/I1-178: 1.000

E8-267/E6-552: 0

E8-267/RP147-603: 0

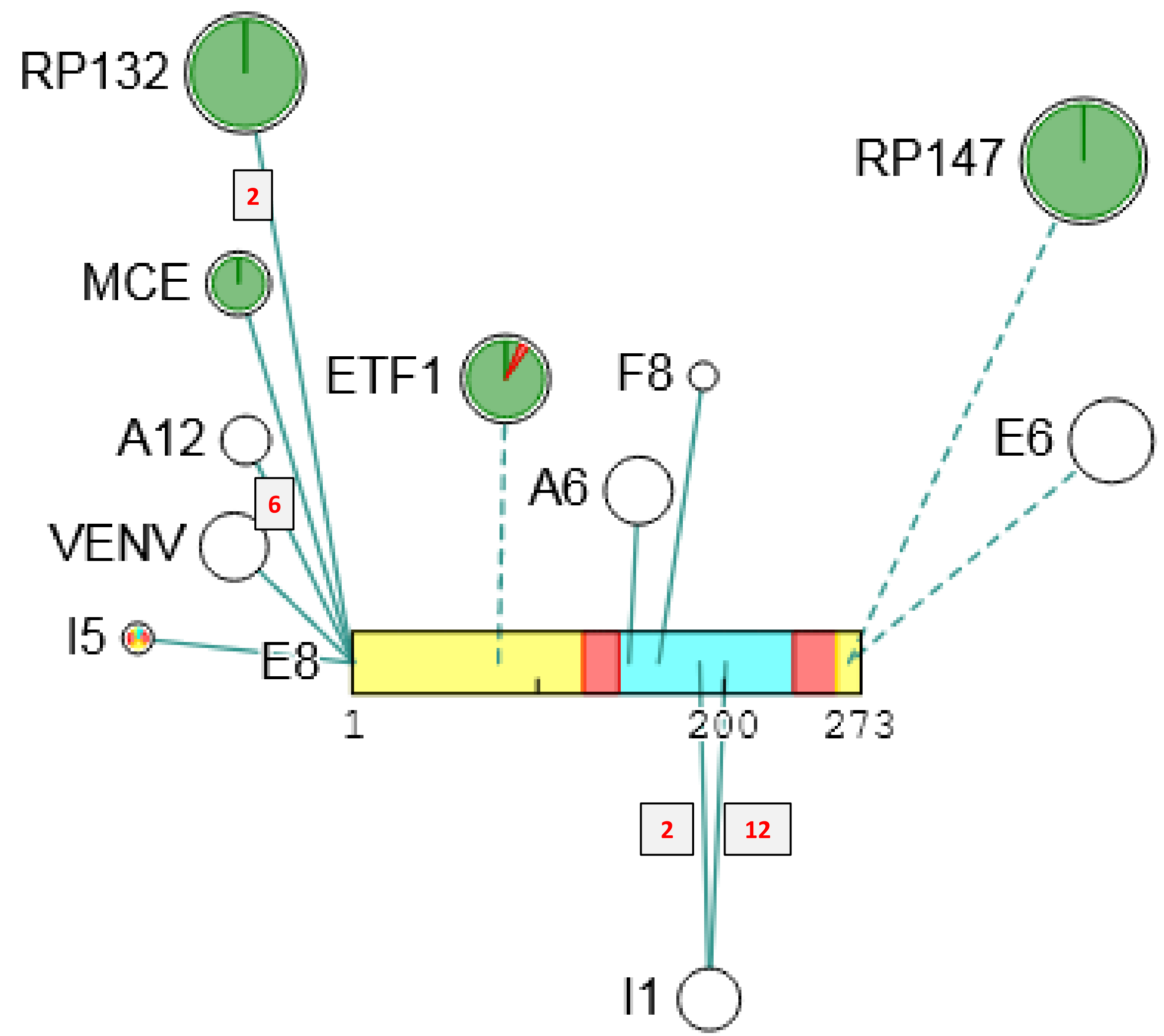

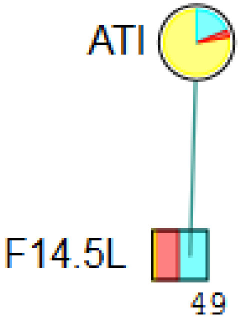
ATI
F14.5L
49

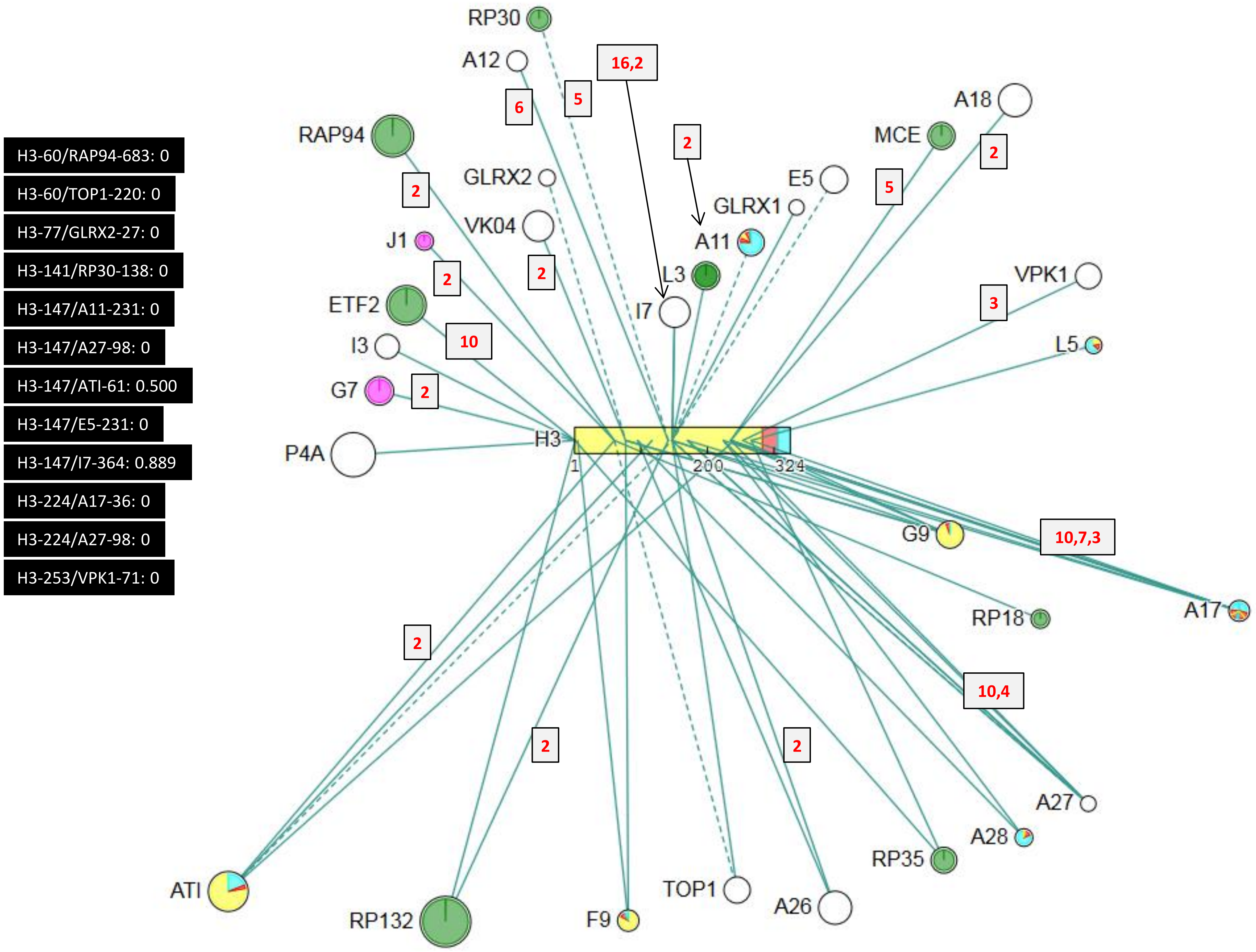
H3-60/RAP94-683: 0
H3-60/TOP1-220: 0
H3-77/GLRX2-27: 0
H3-141/RP30-138: 0
H3-147/A11-231: 0
H3-147/A27-98: 0
H3-147/ATI-61: 0.500
H3-147/E5-231: 0
H3-147/I7-364: 0.889
H3-224/A17-36: 0
H3-224/A27-98: 0
H3-253/VPK1-71: 0
RP30
A12
16,2
6
5
A18
RAP94
2
MCE
2
2
GLRX2
E5
5
GLRX1
2
VK04
A11
J1
2
2
L3
VPK1
ETF2
I7
3
10
I3
L5
G7
2
H3
P4A
1
200
324
G9
10,7,3
RP18
A17
2
10,4
2
2
A27
A28
RP35
ATI
TOP1
A26
RP132
F9

I5 is shown with N-inside topology (next page: TMHMM gives probability for N-inside = 55%).

Below: I5 N-terminal 'inside' (cyan) domain crosslinks to inside domain of E8, only if E8 is in 'Griffiths' topology (four pages back) as opposed to 'TMHMM' topology (three pages back).

PARADOX: TMHMM topology is incorrect for either E8 or I5 according to the E8-I5 crosslink.

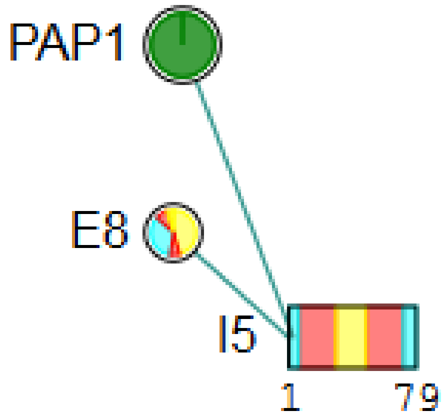

TMHMM result

HELP with output formats

```
# WEBSEQUENCE Length: 79
# WEBSEQUENCE Number of predicted TMHs:  2
# WEBSEQUENCE Exp number of AAs in TMHs: 45.57927
# WEBSEQUENCE Exp number, first 60 AAs:  34.33226
# WEBSEQUENCE Total prob of N-in:        0.55770
# WEBSEQUENCE POSSIBLE N-term signal sequence
WEBSEQUENCE     TMHMM2.0        inside       1     6
WEBSEQUENCE     TMHMM2.0        TMhelix      7    29
WEBSEQUENCE     TMHMM2.0        outside     30    48
WEBSEQUENCE     TMHMM2.0        TMhelix     49    71
WEBSEQUENCE     TMHMM2.0        inside      72    79
```

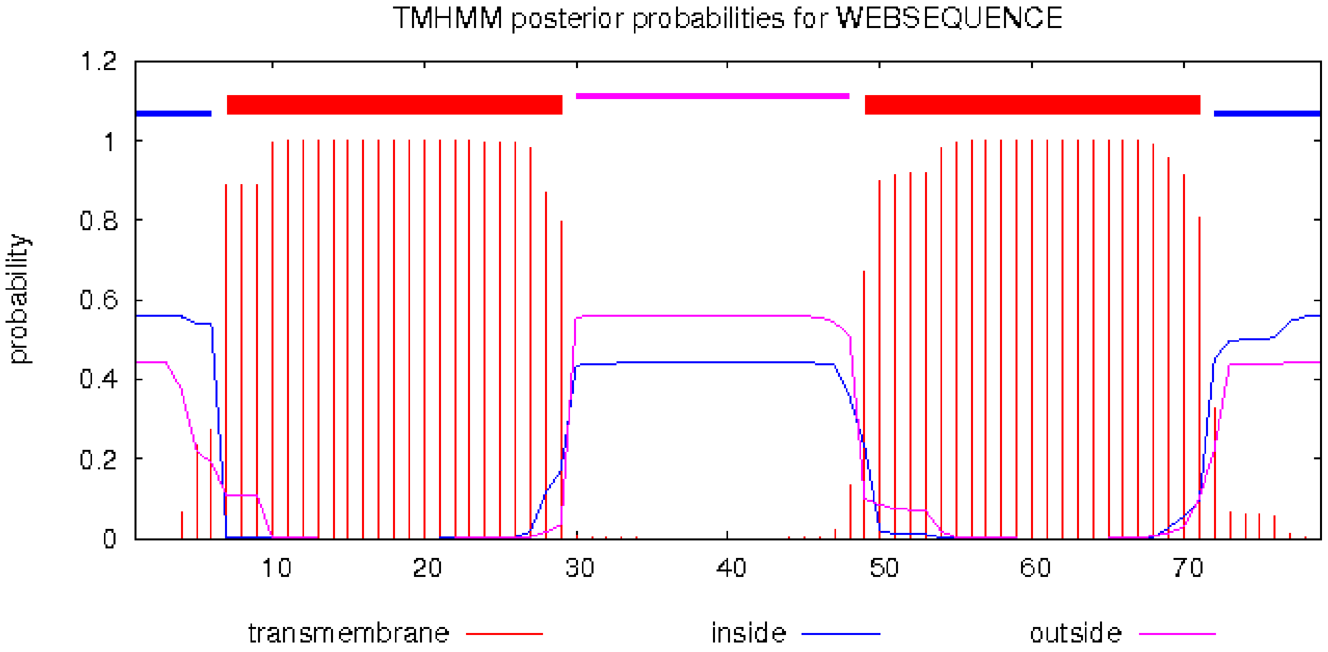

VMAPs

A6, A11

A6-3/MCEL-843: 0.500

A6-136/VP8-175: 0

A6-317/DUSP-8: 0

A6-352/VPK2-205: 0.500

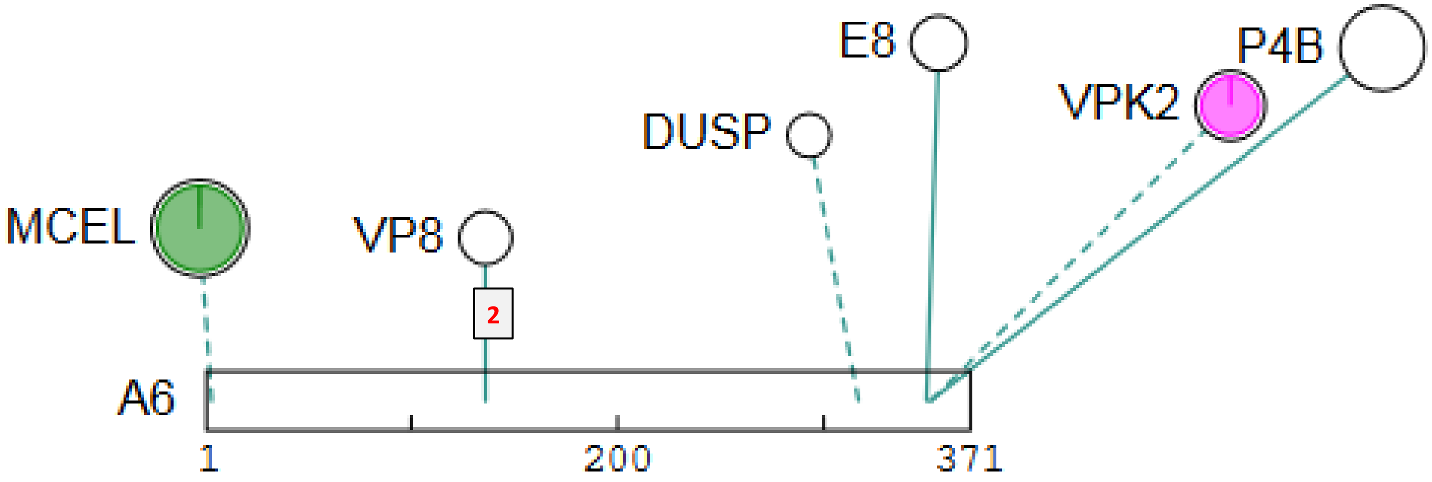

A11-1/MCEL-797: 0.143

A11-185/ETF2-584: 0.500

A11-231/H3-147: 0

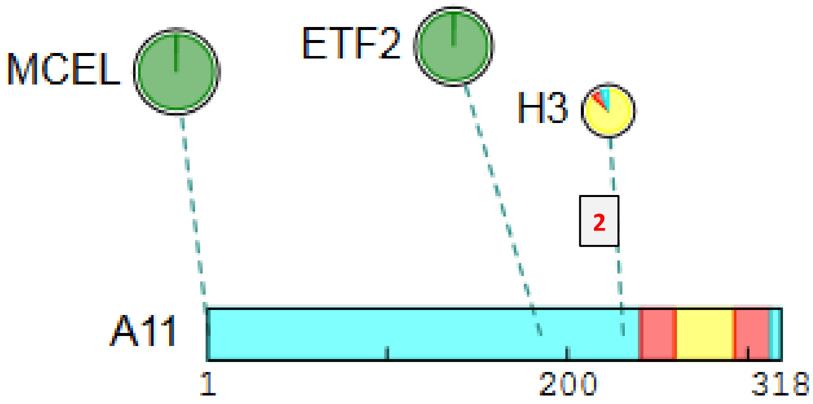

Membrane-associated non-TM proteins

A26, A27, VENV(F13)

A26-37/RP147-147: 0

A26-70/L3-18: 0

A26-306/MCEL-588: 0

A26-380/DNLI-399: 0.500

A26-415/A27-48: 0

A26-415/P4A-660: 0

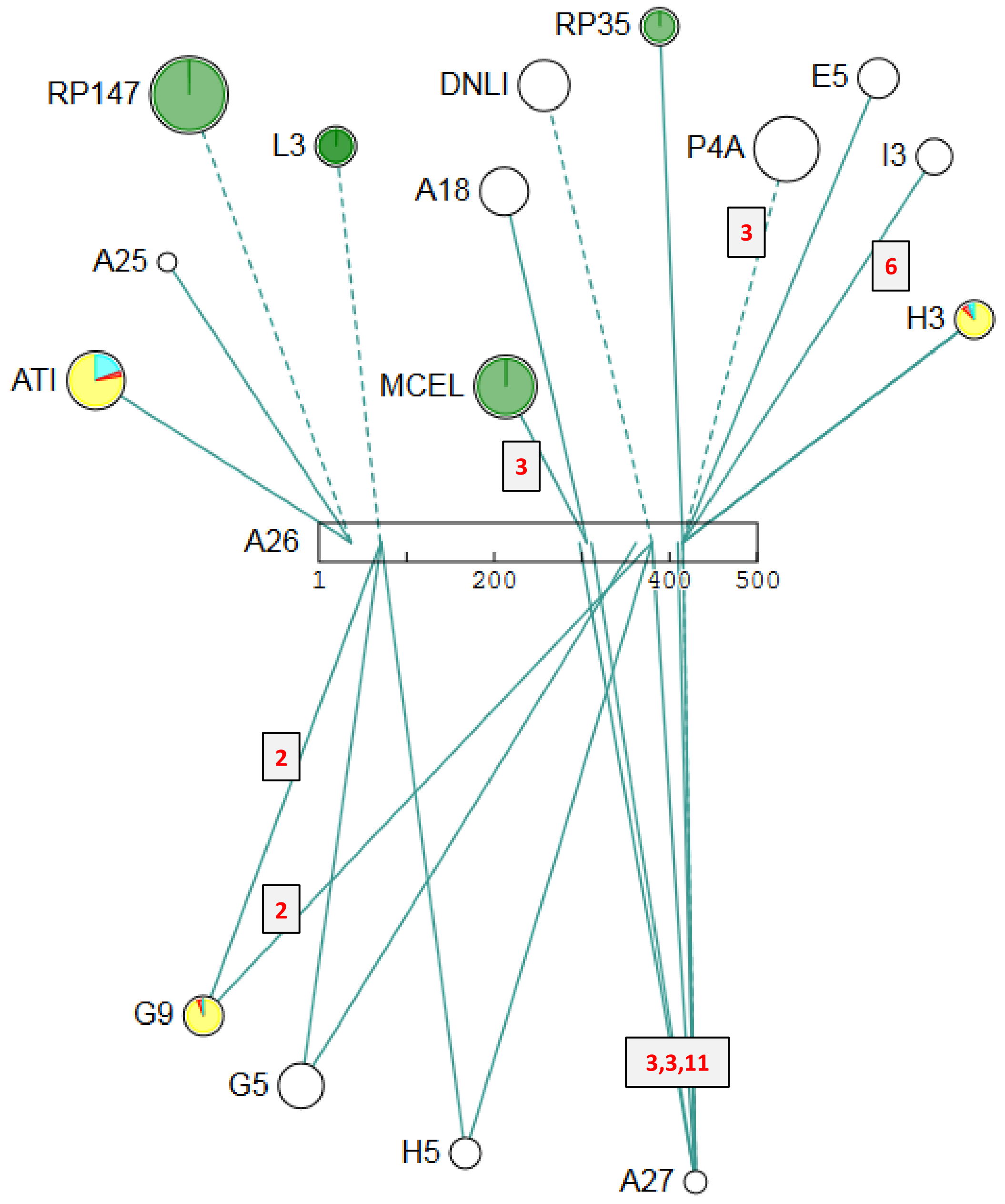

A27-37/A16-116: 0

A27-48/A26-415: 0

A27-57/E5-0: 0

A27-57/RP147-956: 0

A27-98/A17-36: 0

A27-98/H3-224: 0

A27-98/H3-147: 0

A27-99/MCEL-136: 0.500

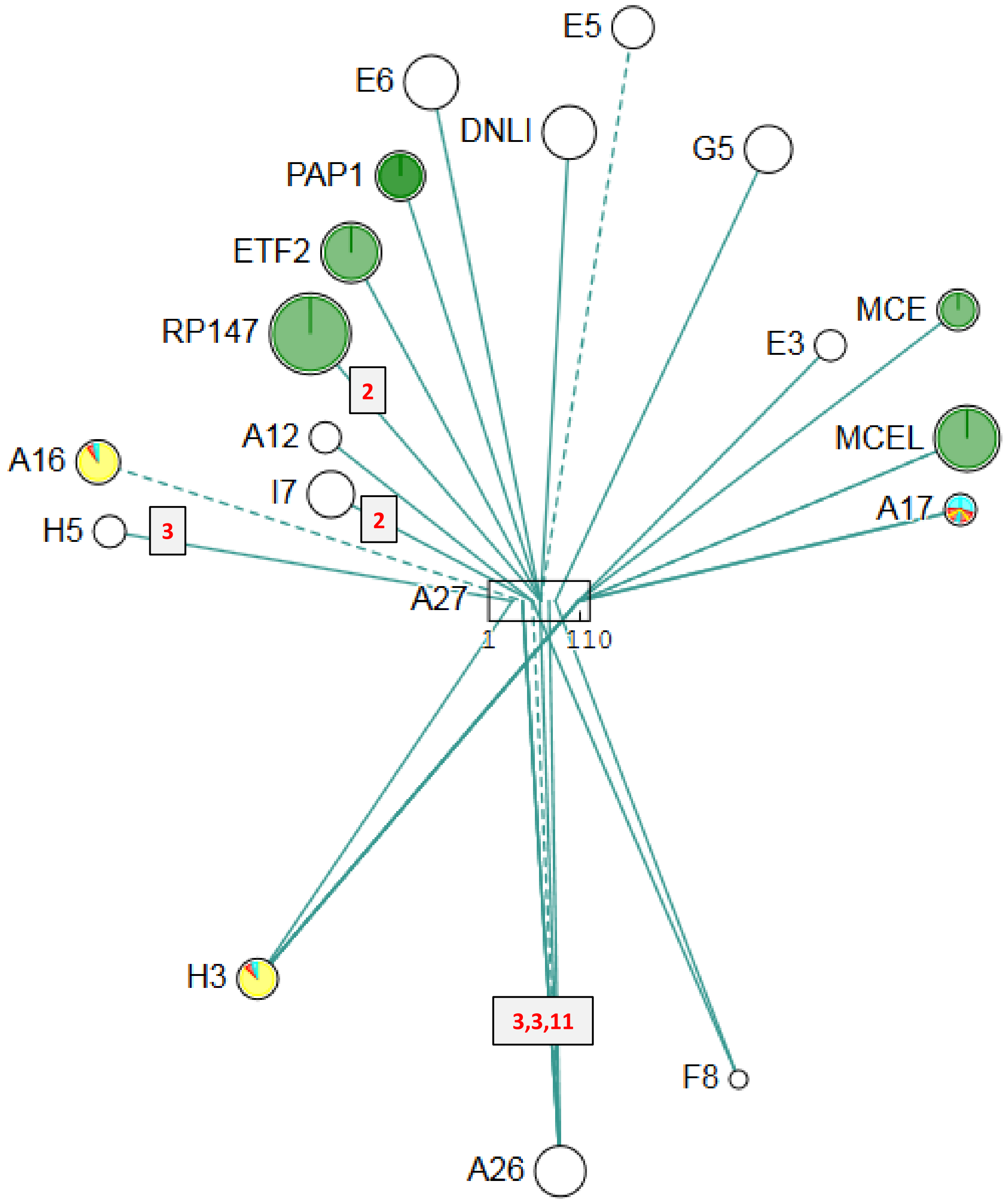

VENV-348/NTP1-324: 0

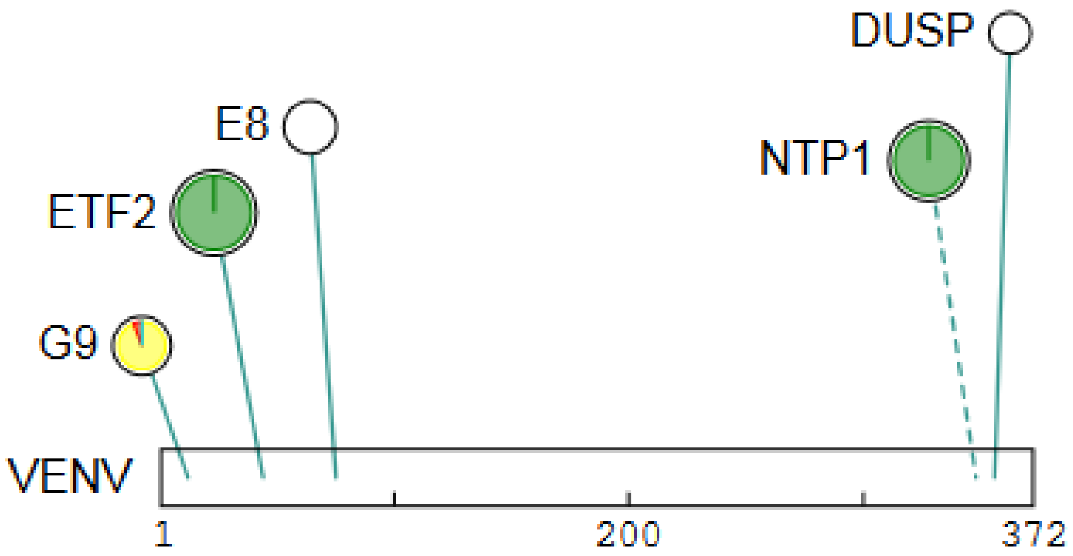

Transcriptosome (mRNA biogenesis) proteins

ETF1, ETF2, L3, MCE, MCEL, MCES, NTP1, PAP1, NPH2, RP147, RP132, RAP94 , RP35 , RP30 , RP22 , RP19 , RP18, RP07

ETF1-229/E8-79: 0

ETF1-252/F17-50: 0

ETF1-308/P4A-736: 0.333

ETF1-575/RP132-421: 0.500

ETF1-575/RP22-1: 0

ETF1-599/ETF2-647: 0

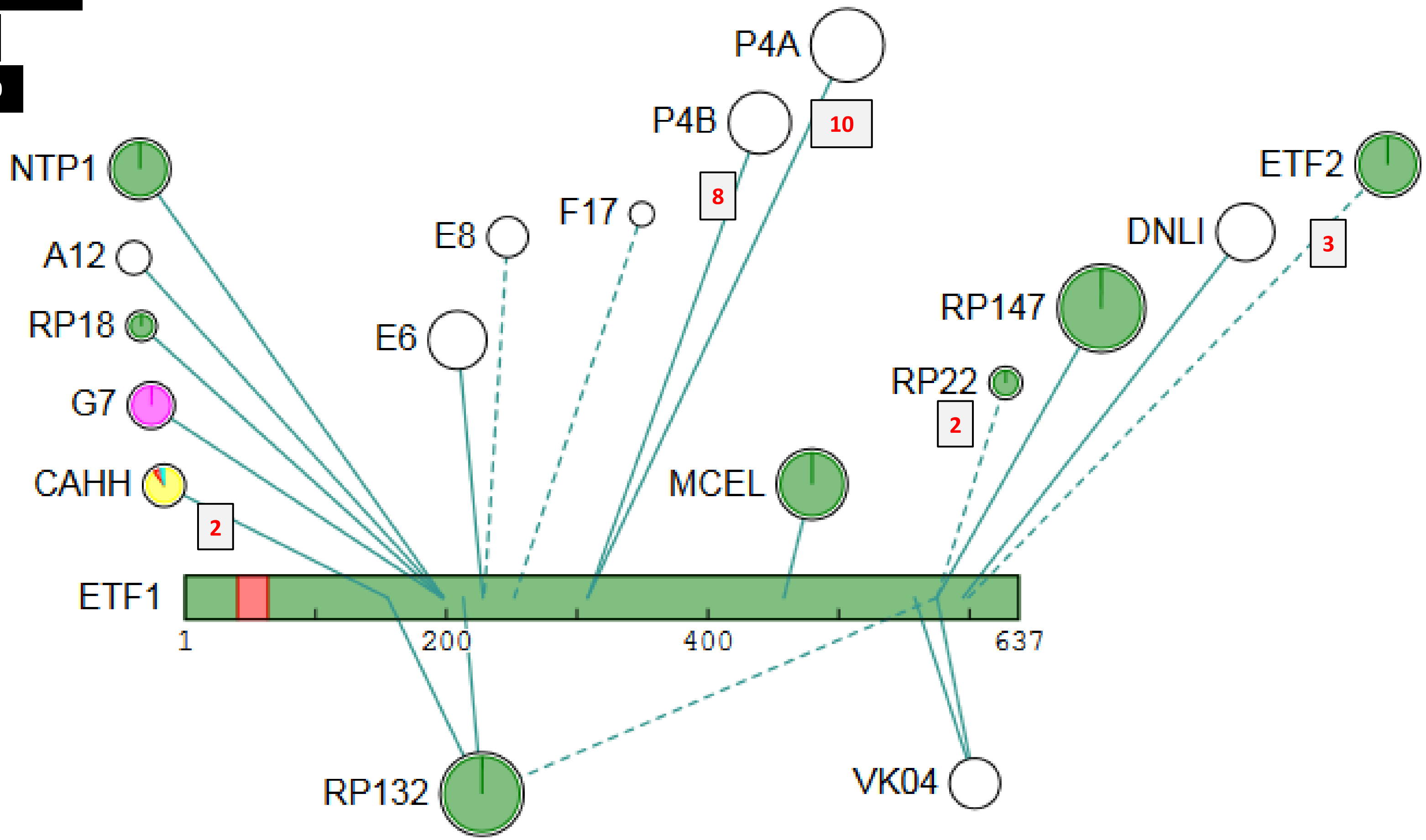

ETF2-67/P4B-185: 0

ETF2-112/I1-193: 0

ETF2-279/J5-82: 0.500

ETF2-381/MCEL-719: 0

ETF2-394/TOP1-207: 0

ETF2-584/A11-185: 0.500

ETF2-607/I6-305: 0.333

ETF2-647/ETF1-599: 0

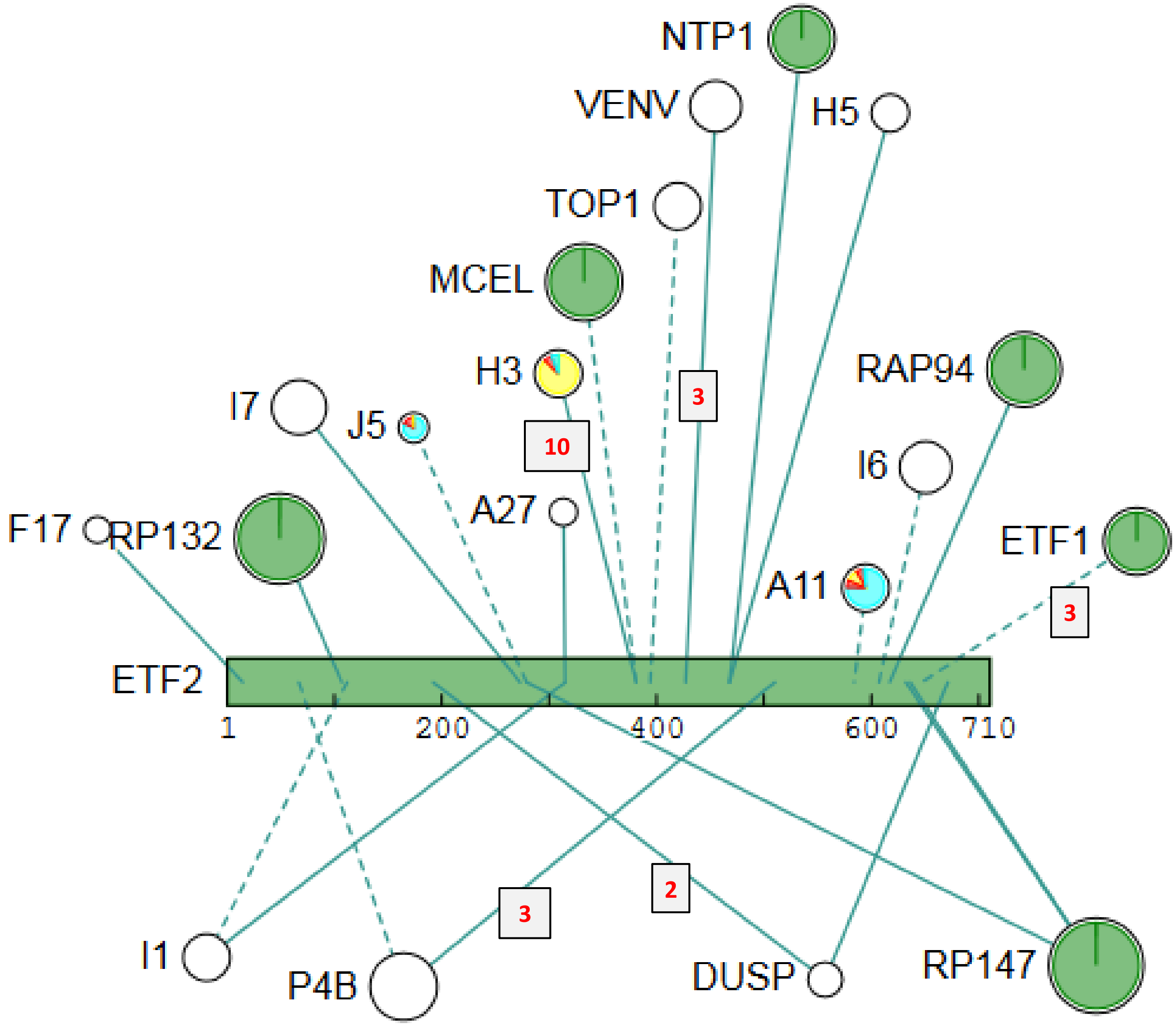

L3-18/A12-167: 0

L3-18/A26-70: 0

L3-22/F17-74: 0

L3-142/E3-165: 0.500

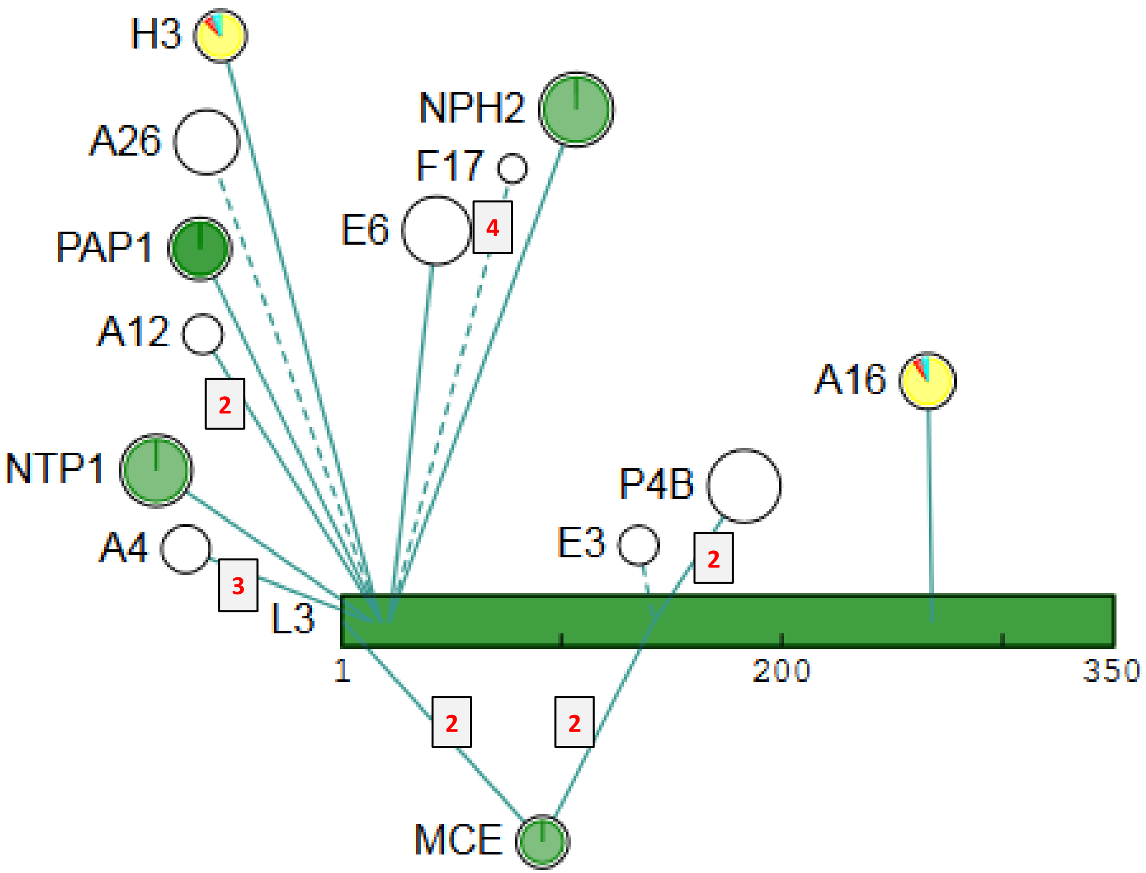

MCE-8/D3-1: 0

MCE-226/G9-58: 0.857

MCE-316/E6-440: 0.500

MCE-321/P4A-876: 0.018

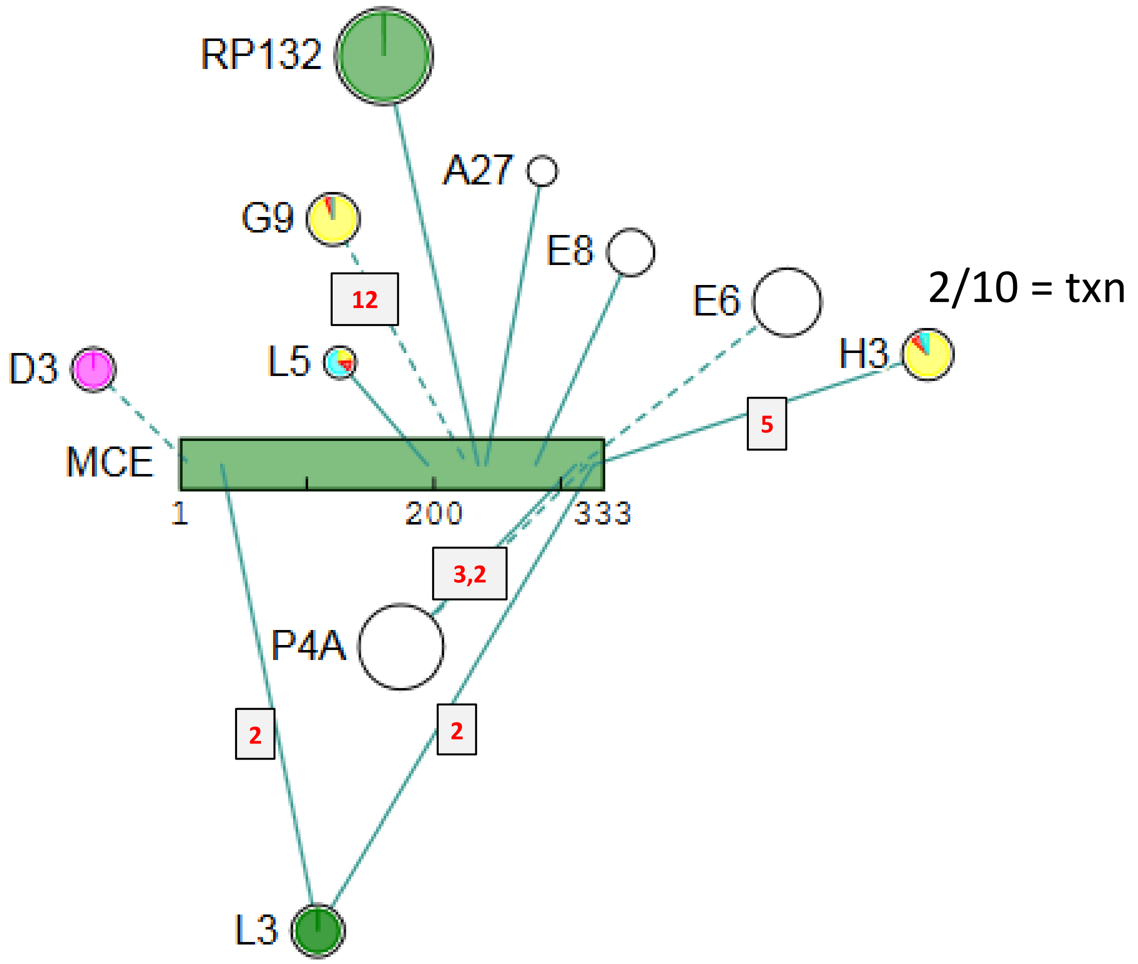

MCEL-136/A27-99: 0.500

MCEL-136/G5-87: 0.500

MCEL-136/RP18-47: 1.000

MCEL-308/NTP1-280: 0

MCEL-386/RP19-156: 0

MCEL-433/P4B-83: 0.500

MCEL-559/RP132-1046: 0

MCEL-588/A26-306: 0

MCEL-719/ETF2-381: 0

MCEL-797/A11-1: 0.143

MCEL-843/A6-3: 0.500

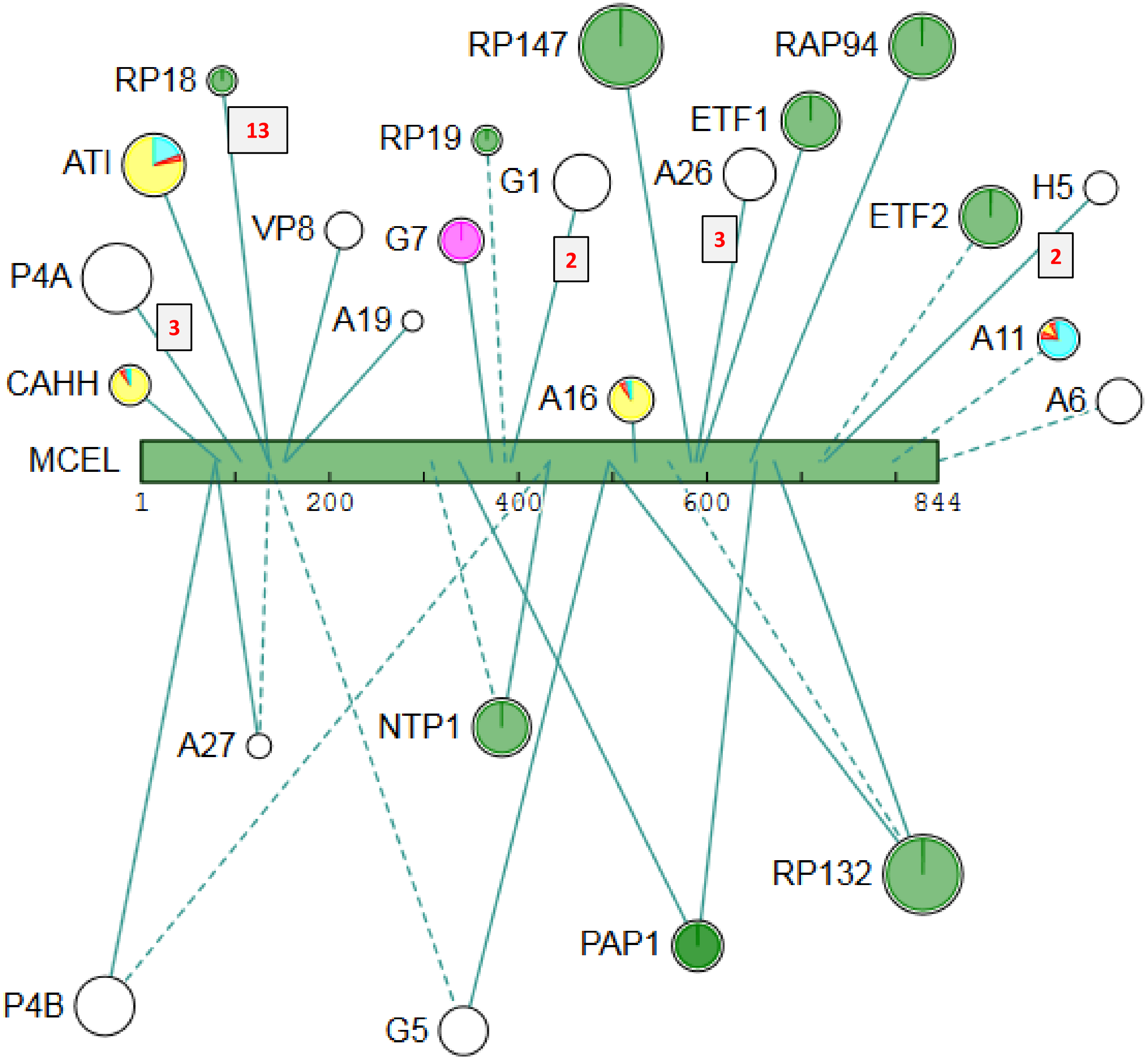

MCES-6/GLRX1-35: 0

MCES-95/PAP1-170: 0.500

MCES-223/RP147-242: 0

MCES-277/P4B-83: 0.500

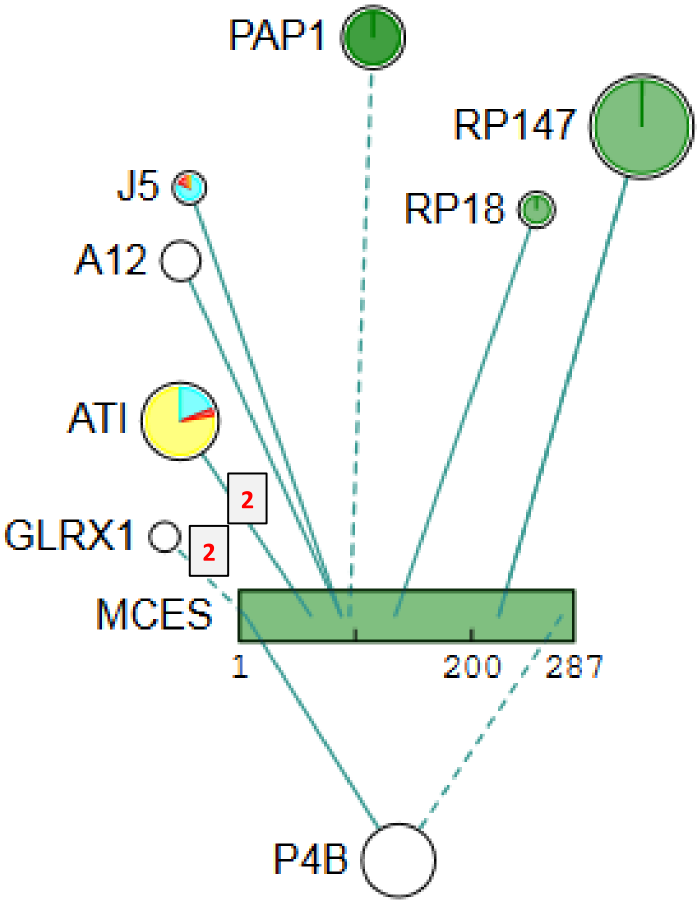

NTP1-32/E11-62: 0
NTP1-56/G5-337: 1.000
NTP1-56/G5-350: 1.000
NTP1-153/RP147-724: 0.500
NTP1-175/RAP94-177: 1.000
NTP1-280/MCEL-308: 0
NTP1-324/VENV-348: 0
NTP1-569/F17-74: 0

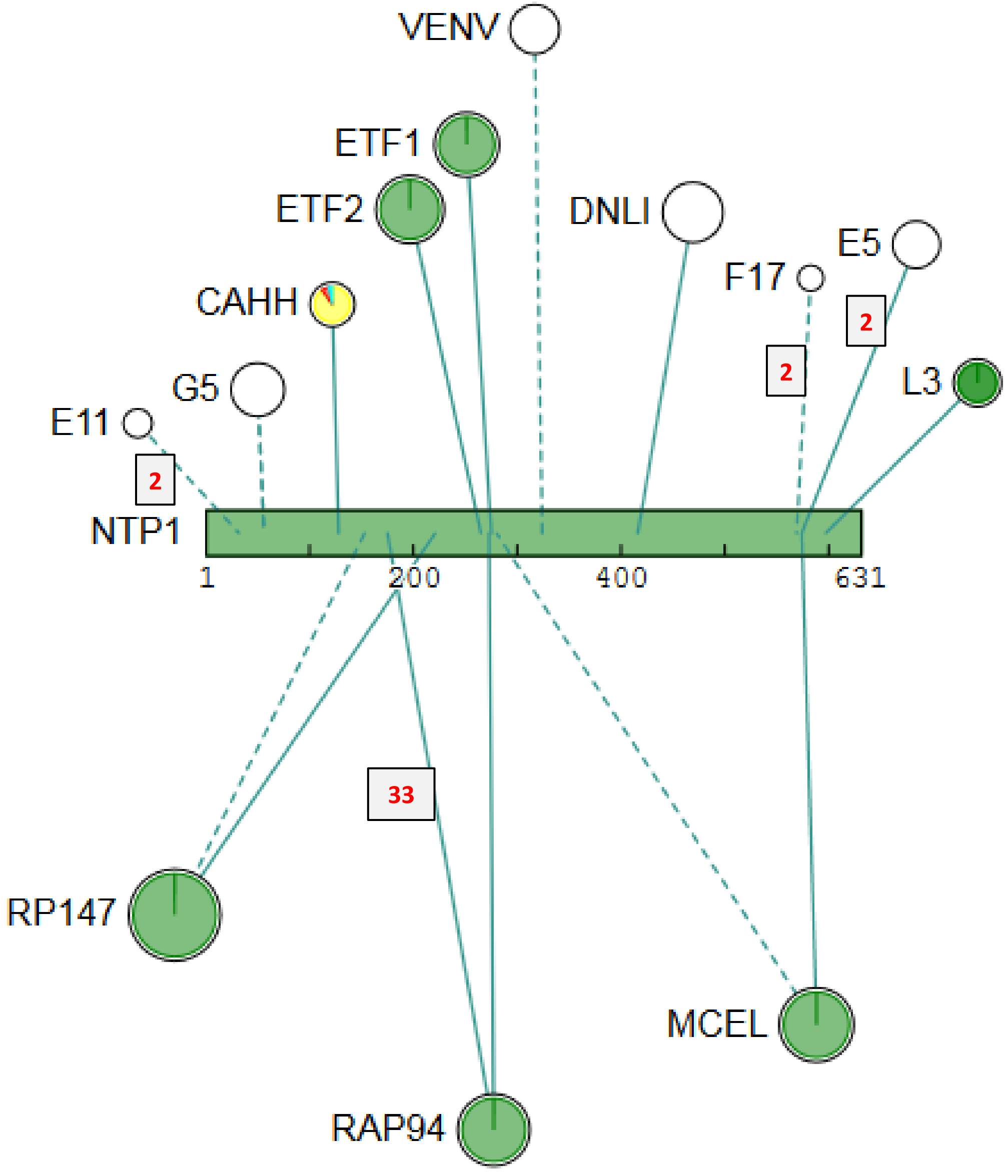

PAP1-170/MCES-95: 0.500

PAP1-462/A12-167: 0

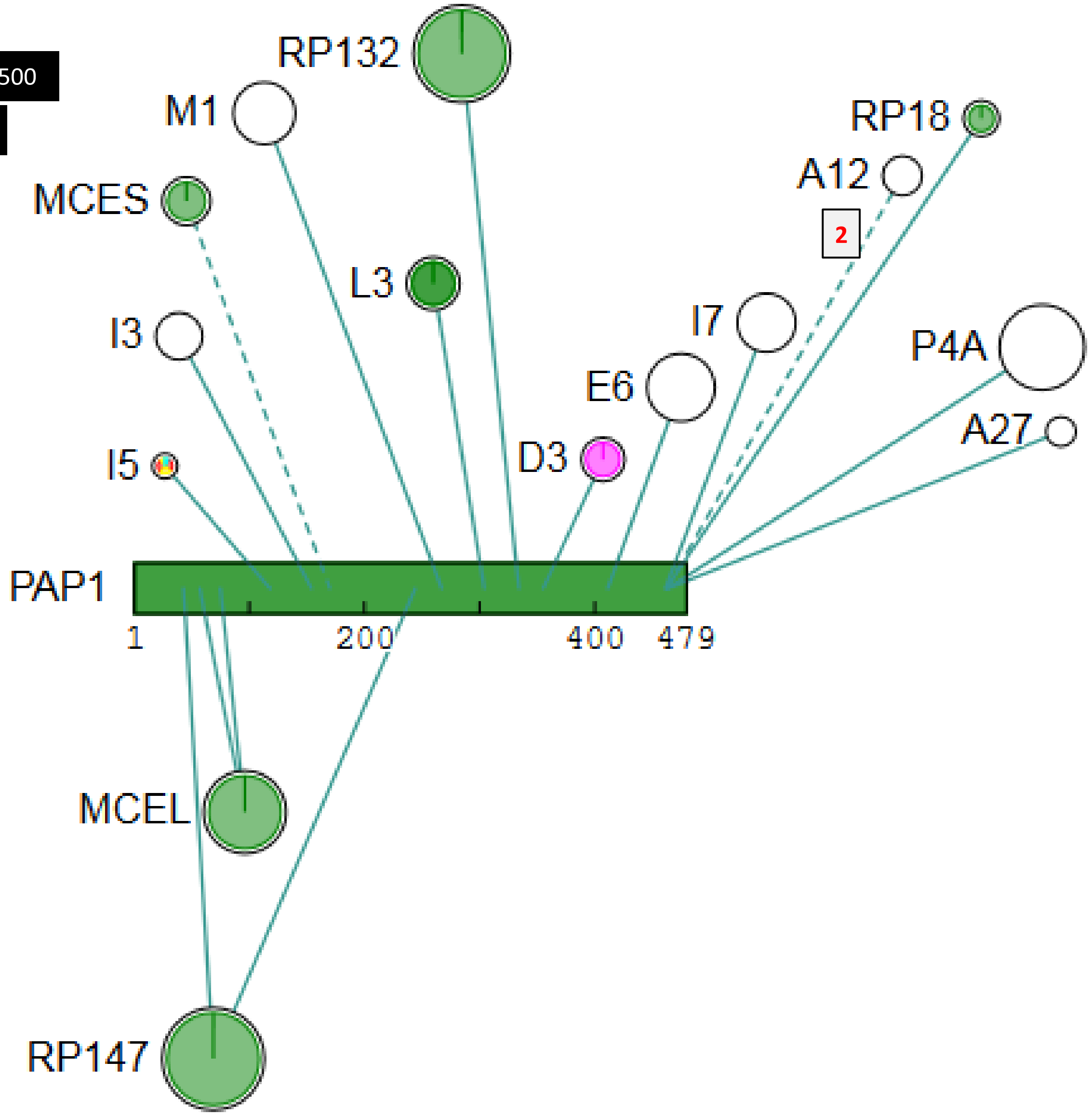

NPH2-71/I1-37: 0

NPH2-622/P4A-876: 0.167

NPH2-627/P4A-508: 0

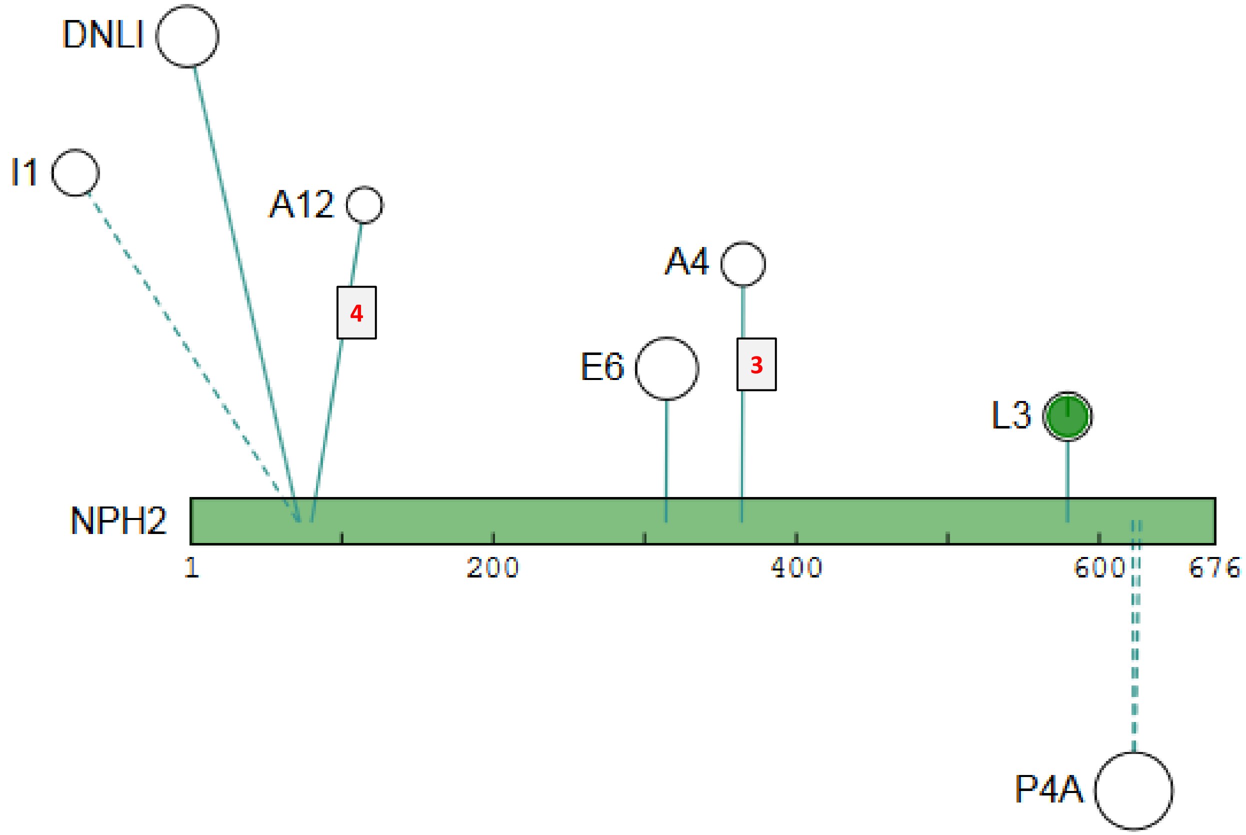

RP147-1/VP8-153: 0

RP147-37/F17-17: 0

RP147-54/D3-7: 0

RP147-75/G7-1: 0

RP147-127/E5-314: 0

RP147-147/A26-37: 0

RP147-148/F9-203: 0.111

RP147-242/MCES-223: 0

RP147-493/A16-153: 0

RP147-566/A19-1: 0

RP147-566/I7-53: 0

RP147-566/TOP1-74: 0

RP147-603/E8-267: 0

RP147-724/NTP1-153: 0.500

RP147-951/N1-78: 0

RP147-956/A27-57: 0

RP147-956/G9-55: 0.500

RP147-974/RP132-452: 0.500

RP147-1029/P4A-876: 0.286

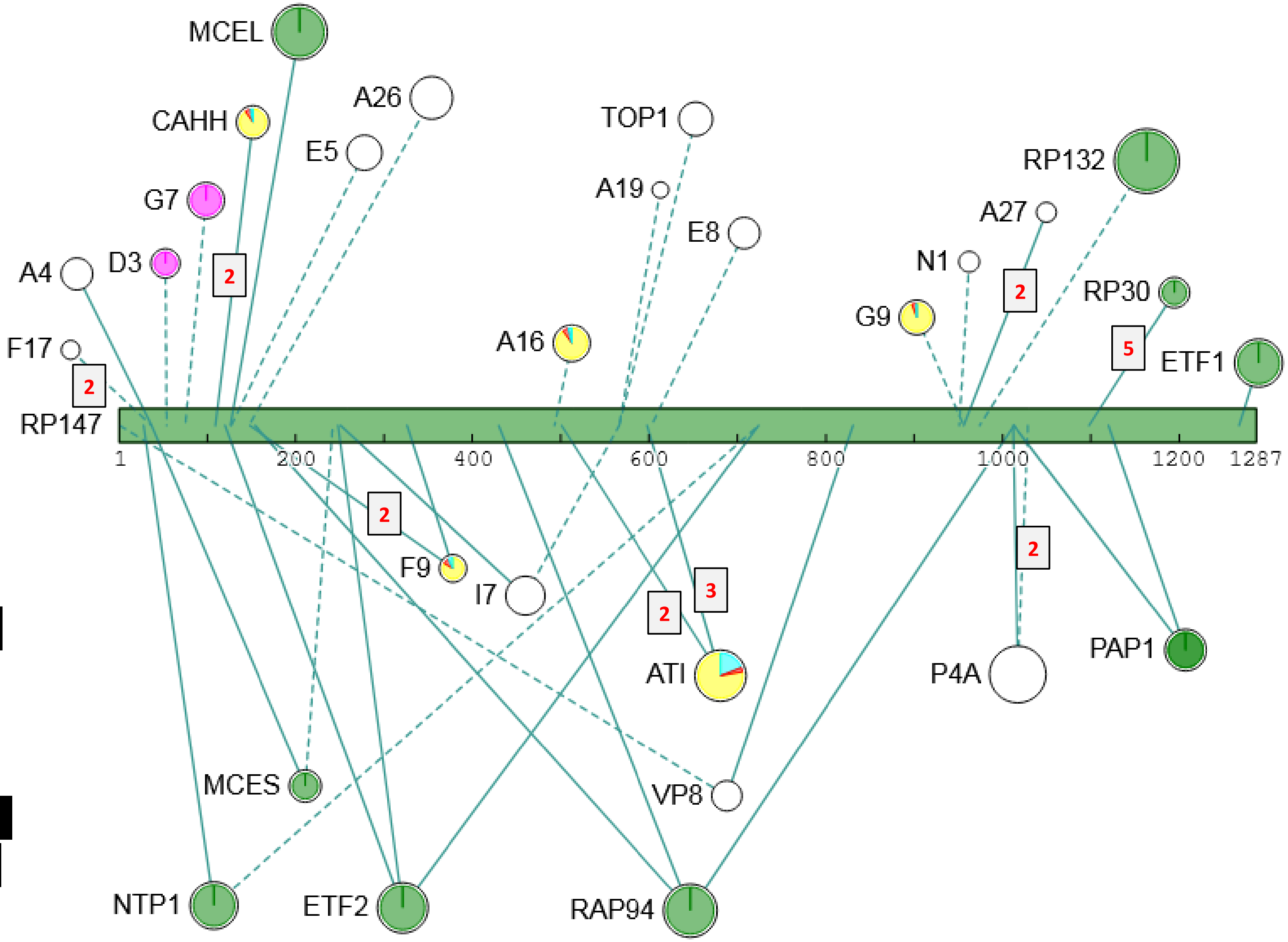

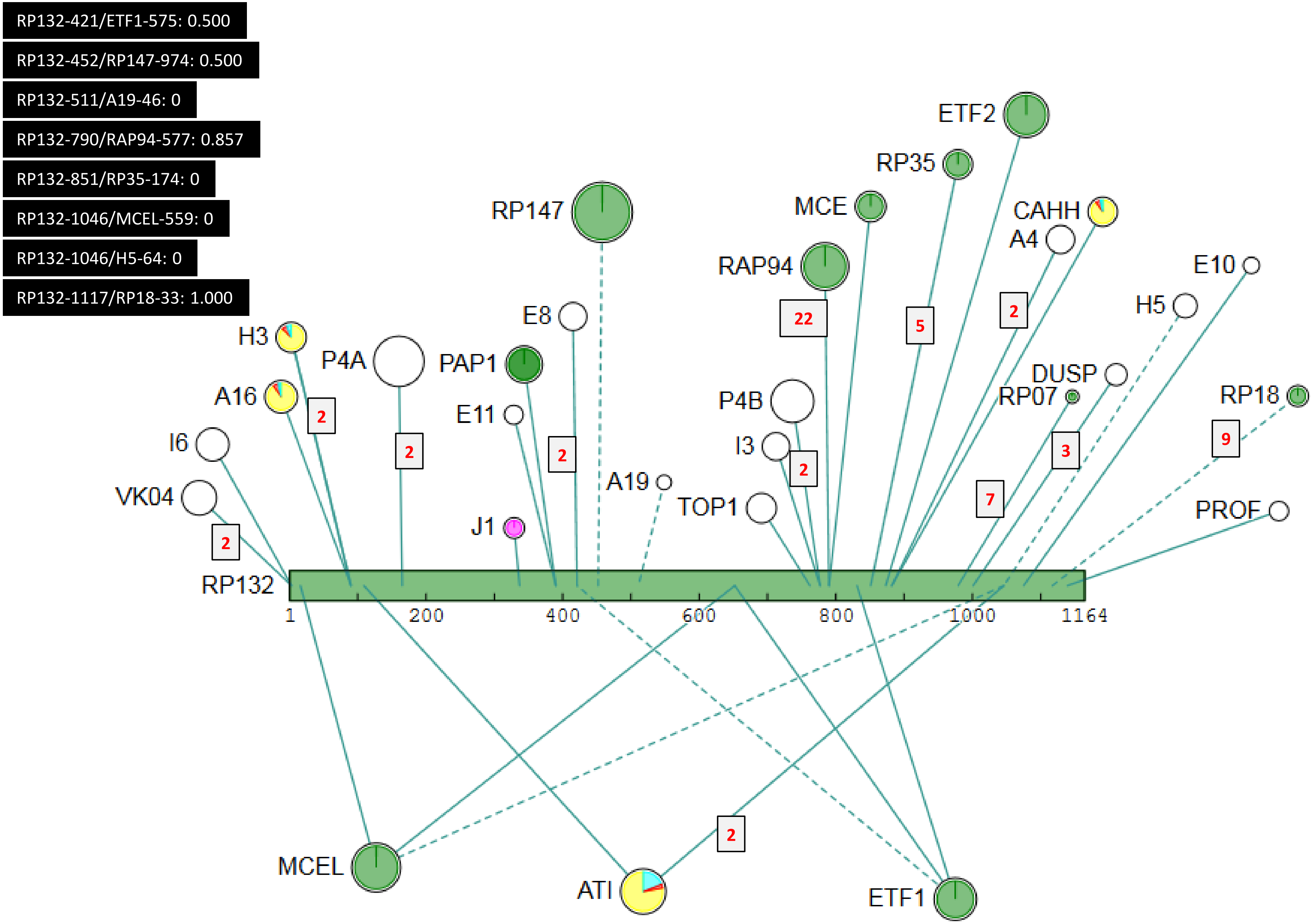
RP132-421/ETF1-575: 0.500
RP132-452/RP147-974: 0.500
RP132-511/A19-46: 0
RP132-790/RAP94-577: 0.857
RP132-851/RP35-174: 0
RP132-1046/MCEL-559: 0
RP132-1046/H5-64: 0
RP132-1117/RP18-33: 1.000
ETF2
RP35
MCE
CAHH
A4
RP147
RAP94
E10
22
5
2
H5
E8
H3
P4A
PAP1
DUSP
RP07
RP18
A16
2
P4B
E11
2
2
I6
I3
2
3
9
A19
VK04
TOP1
7
PROF
J1
2
RP132
1
200
400
600
800
1000
1164
2
MCEL
ATI
ETF1

RAP94-177/NTP1-175: 1.000

RAP94-206/E5-61: 0

RAP94-333/GLRX1-1: 0.500

RAP94-516/A16-22: 0

RAP94-577/RP132-790: 0.857

RAP94-683/H3-60: 0

RAP94-683/RP30-101: 0.500

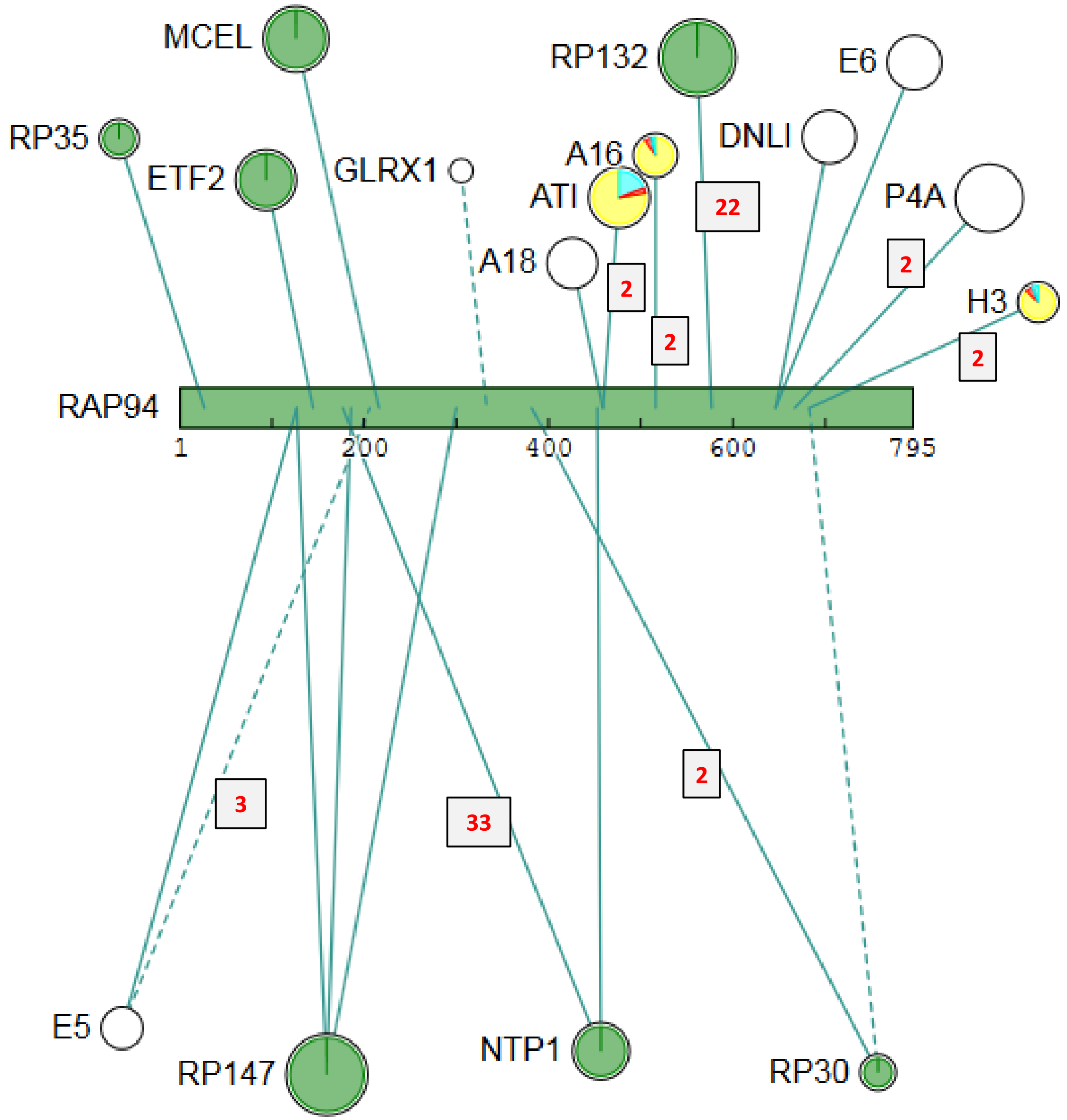

RP35-67/DNLI-477: 0

RP35-174/RP132-851: 0

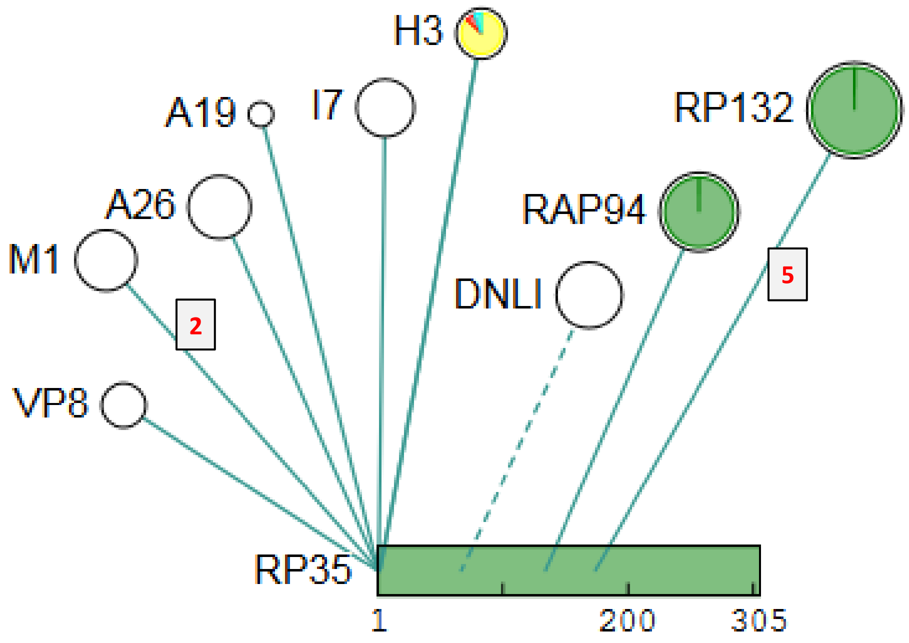

RP30-54/E3-3: 0.500

RP30-80/VP8-153: 0

RP30-85/P4B-83: 0

RP30-101/RAP94-683: 0.500

RP30-138/H3-141: 0

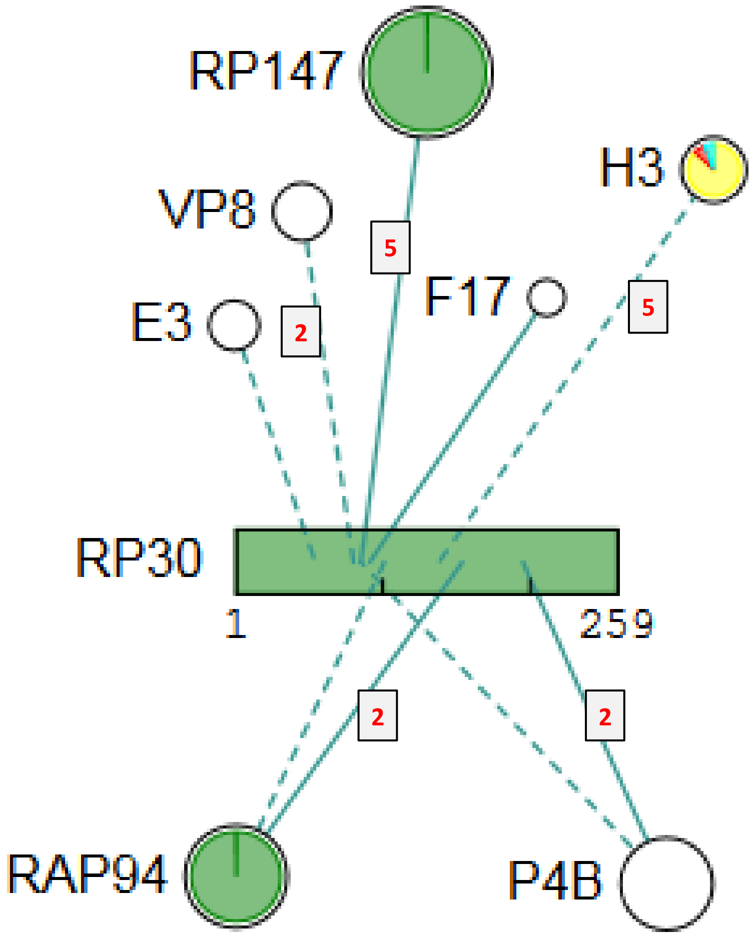

RP22-0/G5-90: 0

RP22-1/ETF1-575: 0

RP22-1/G9-57: 0

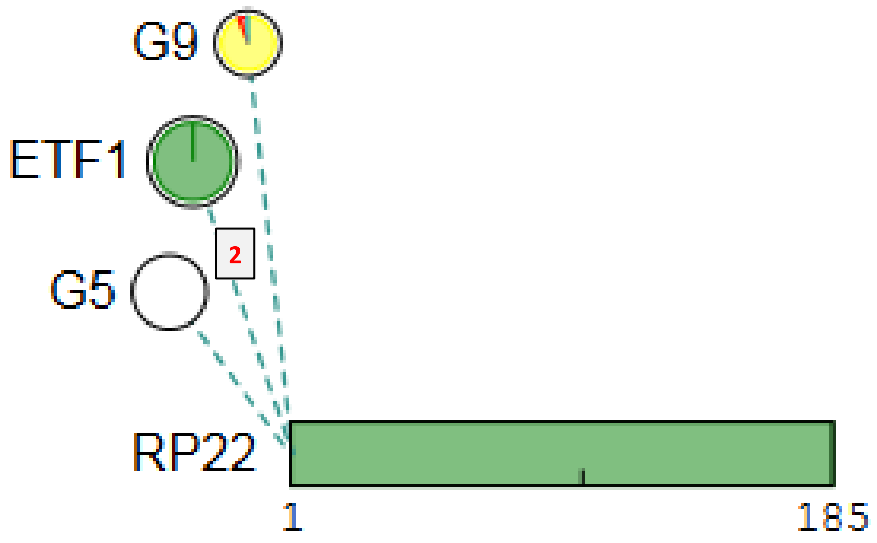

RP19-13/E6-404: 1.000

RP19-14/E6-404: 1.000

RP19-156/MCEL-386: 0

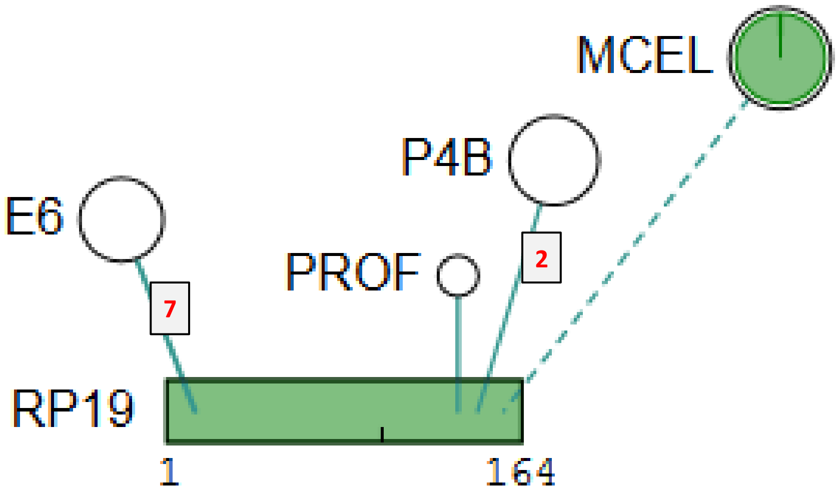

RP18-33/RP132-1117: 1.000

RP18-47/MCEL-136: 1.000

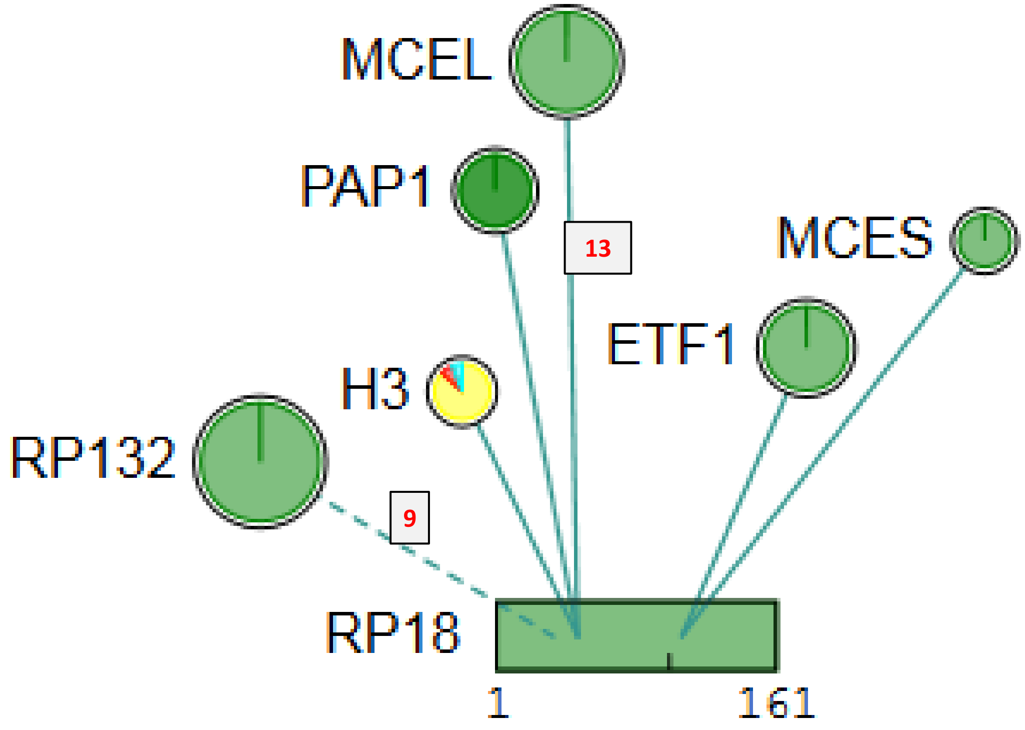

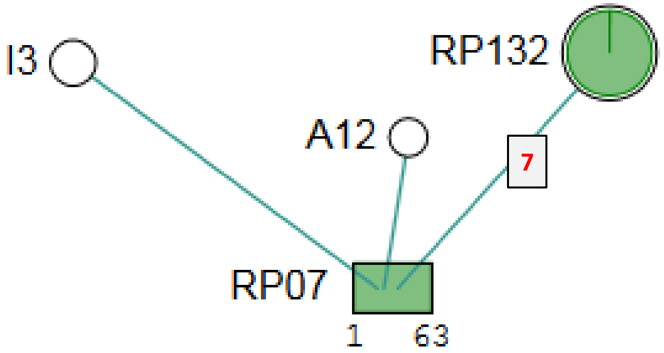
I3
A12
RP132
7
RP07
1
63

DNA-related proteins

DNLI, G5, H5, I1, I3, I6, K4, TOP1, VP8

DNLI-0/P4B-83: 0

DNLI-177/A19-0: 0.500

DNLI-303/VPK2-1: 0.667

DNLI-397/H5-25: 0

DNLI-399/A26-380: 0.500

DNLI-477/RP35-67: 0

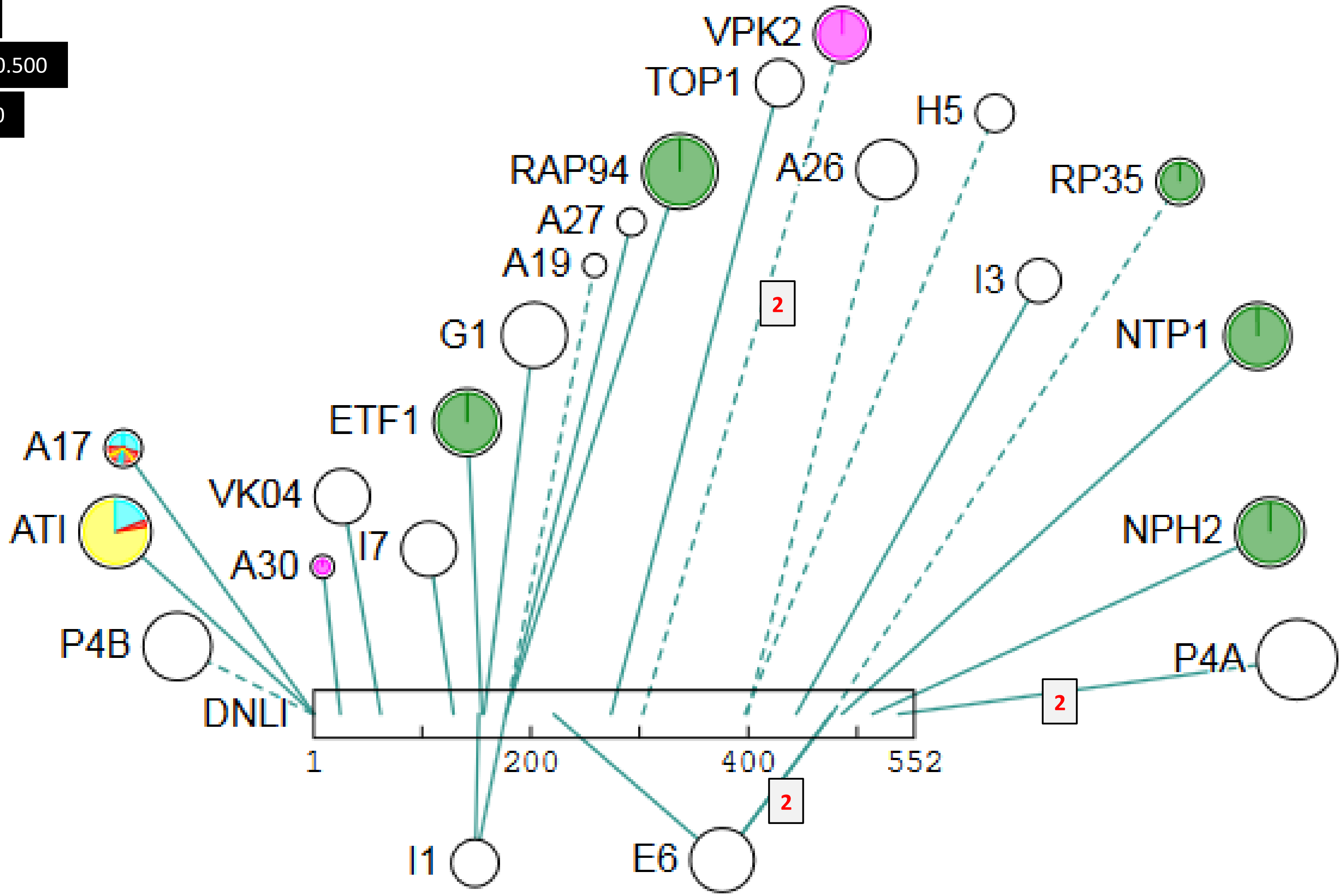

G5-87/MCEL-136: 0.500

G5-90/RP22-0: 0

G5-337/NTP1-56: 1.000

G5-350/NTP1-56: 1.000

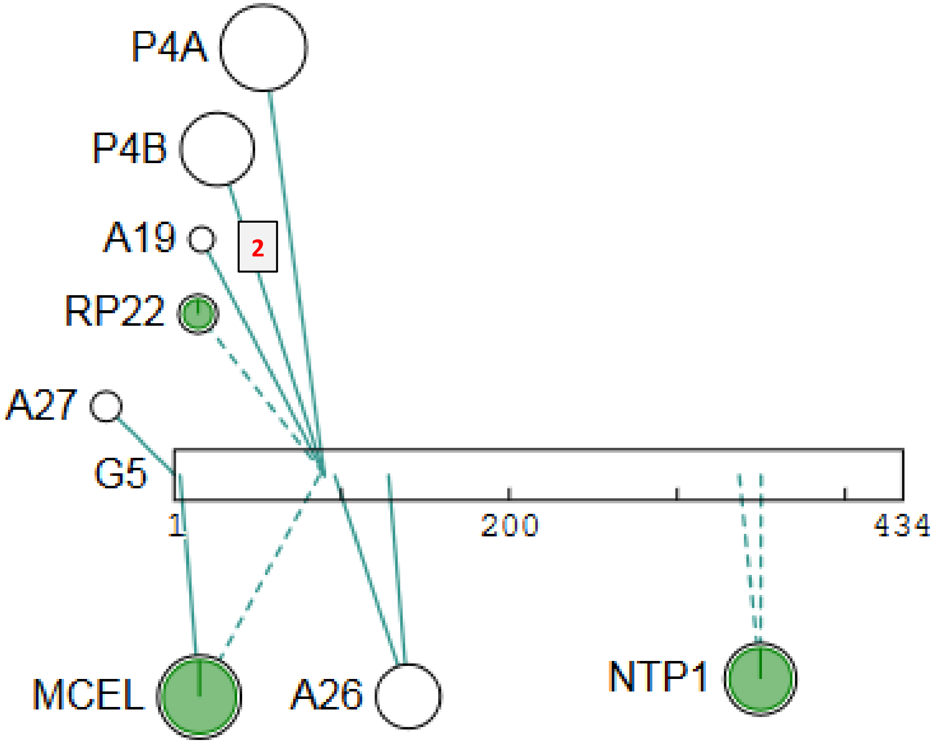

H5-1/E11-56: 0

H5-25/DNLI-397: 0

H5-25/P4A-487: 0.500

H5-64/RP132-1046: 0

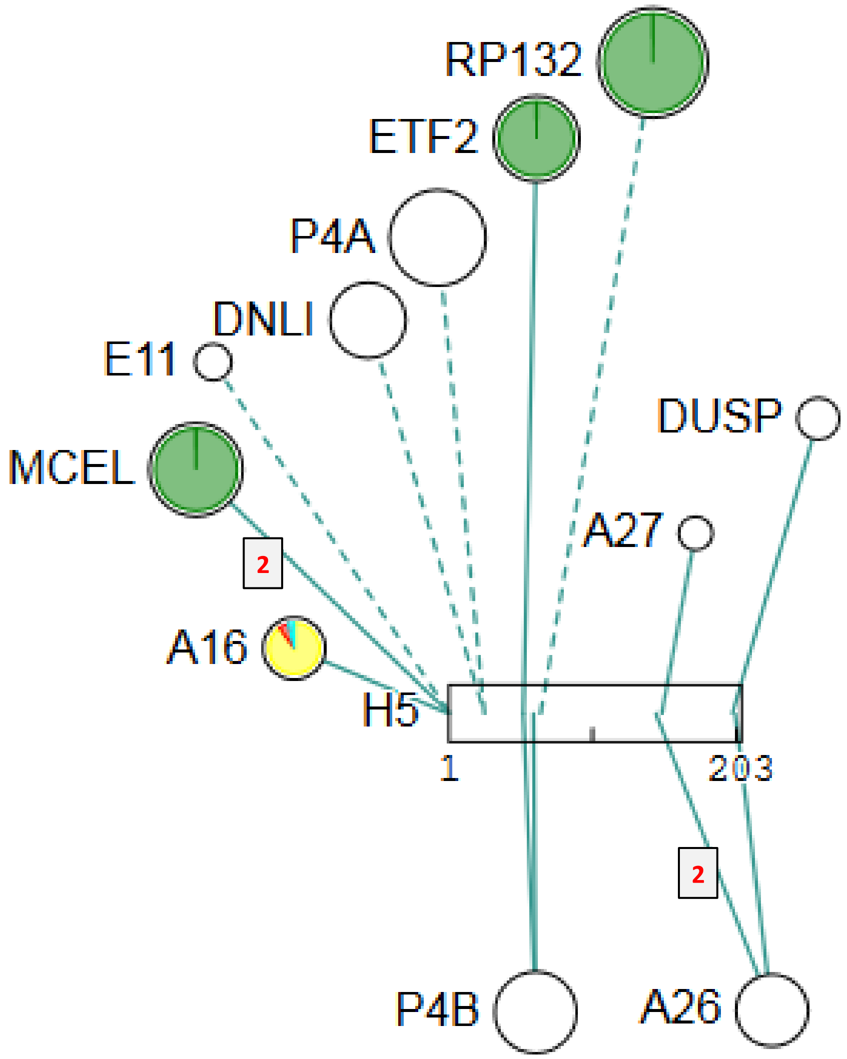

I1-37/NPH2-71: 0

I1-41/I6-359: 0

I1-178/E8-201: 1.000

I1-193/ETF2-112: 0

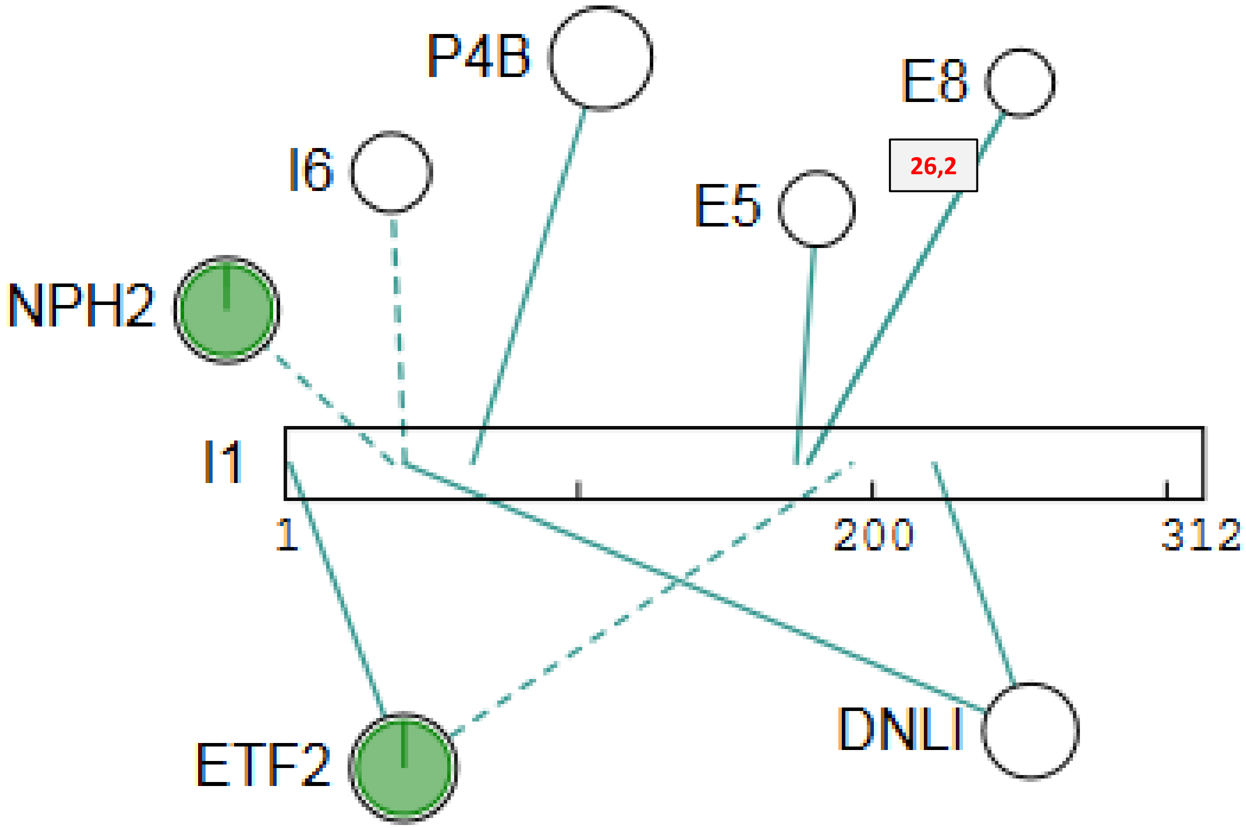

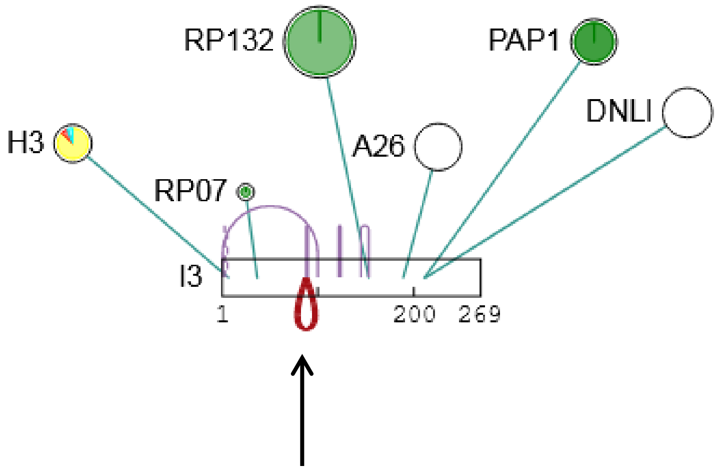

Self-interaction region of Greseth *et al*. [Traktman] 2012? At least three overlaid self-links (86-86, 88-88, 90-90)

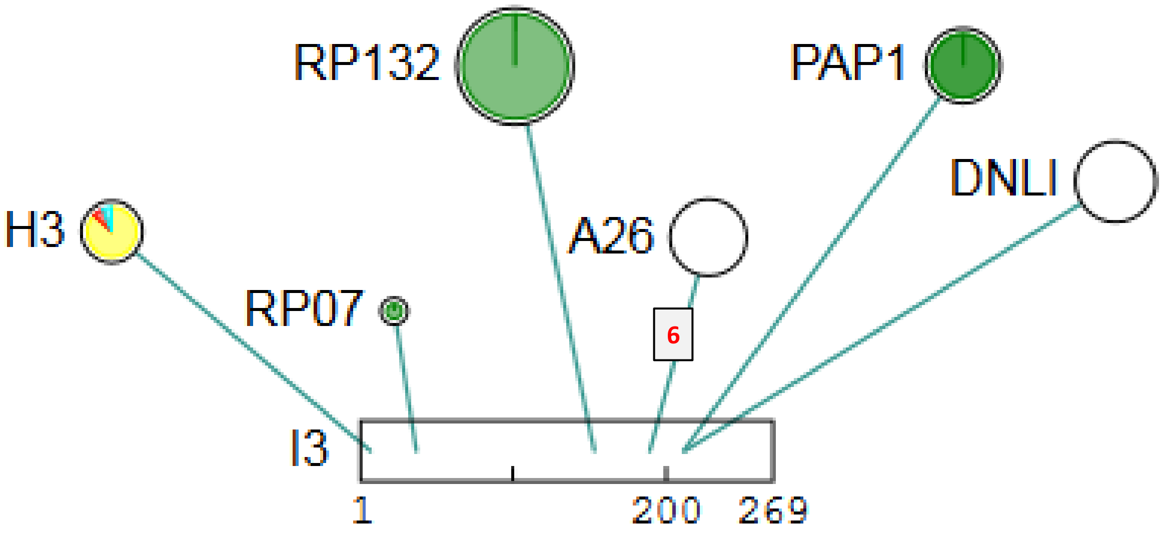

I6-305/ETF2-607: 0.333

I6-354/P4B-134: 0

I6-359/I1-41: 0

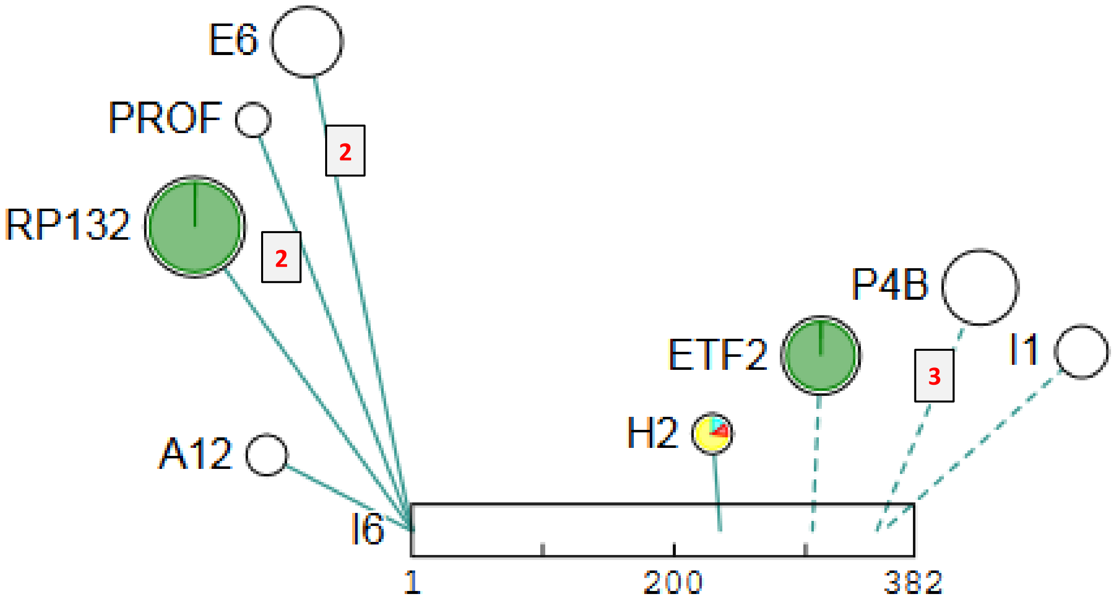

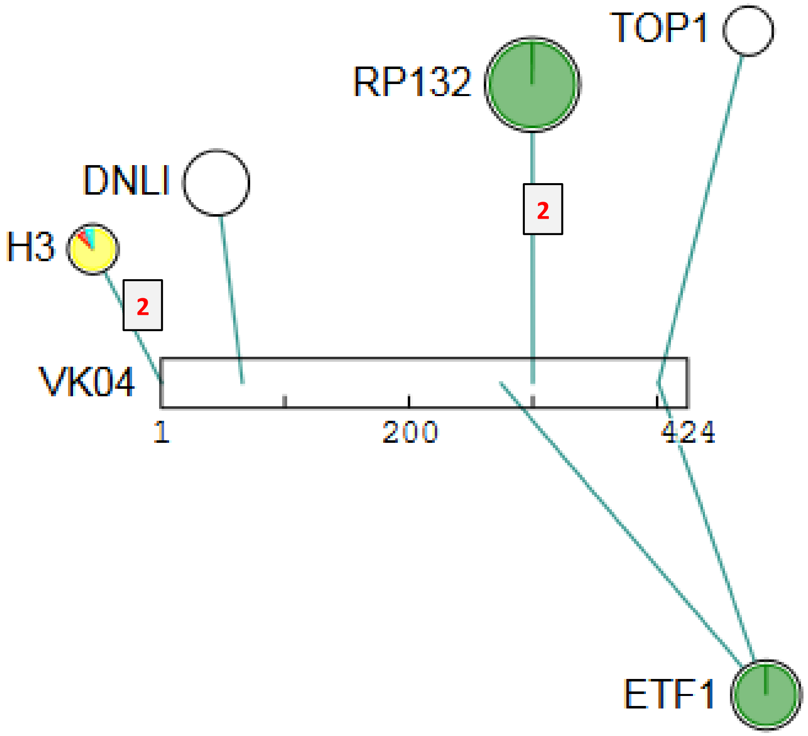
TOP1
RP132
DNLI
H3
2
2
VK04
1
200
424
ETF1

TOP1-74/RP147-566: 0

TOP1-167/P4A-876: 0.500

TOP1-169/I7-414: 0.500

TOP1-169/P4A-1: 0

TOP1-207/ETF2-394: 0

TOP1-207/P4A-876: 0

TOP1-220/H3-60: 0

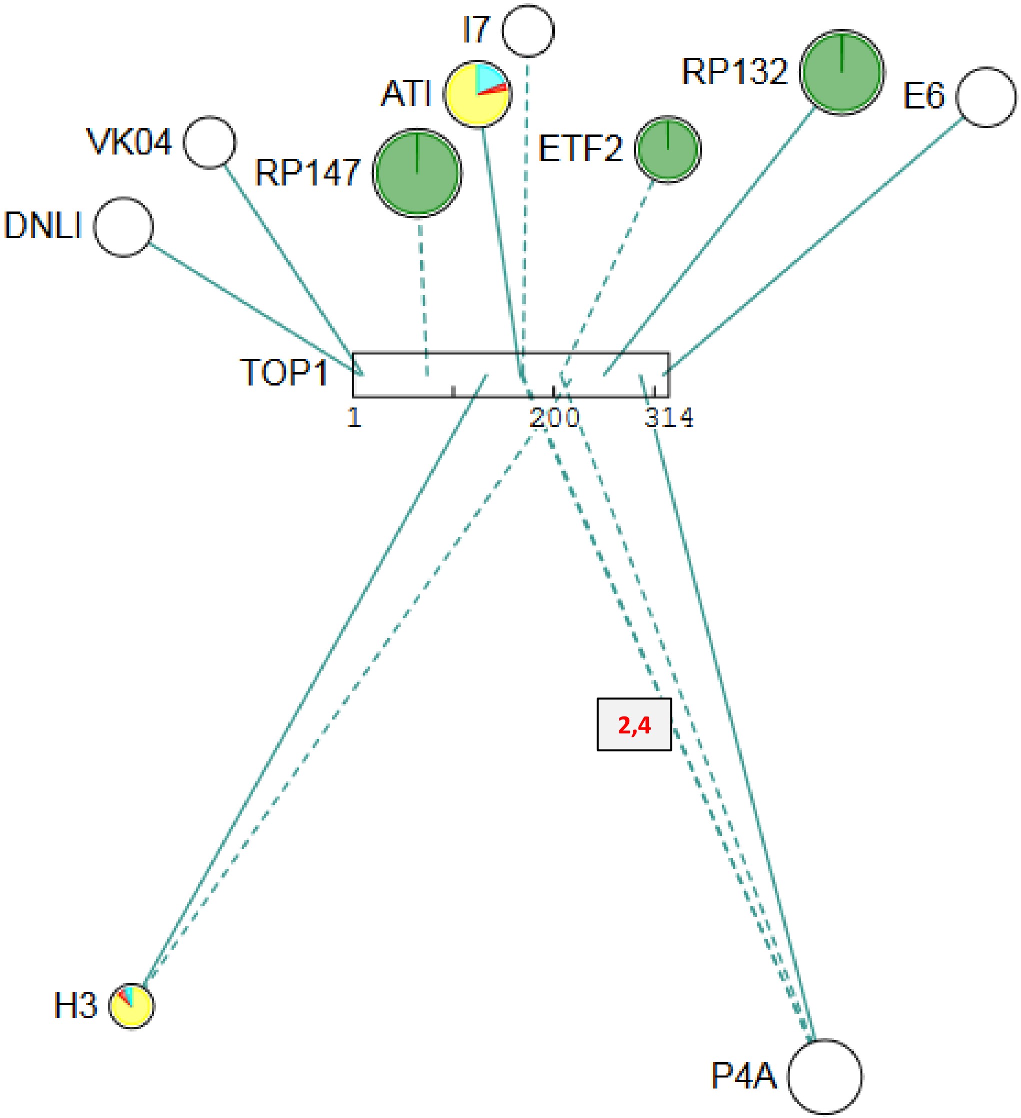

VP8-125/P4B-83: 0
VP8-153/RP147-1: 0
VP8-153/RP30-80: 0
VP8-175/A6-136: 0
VP8-204/A12-75: 0
VP8-212/A12-63: 0
VP8-240/A12-63: 0
VP8-240/A12-75: 0
VP8-240/A12-88: 1.000
VP8-240/A12-166: 0
VP8-240/E3-40: 0
VP8-250/P4A-487: 0.500

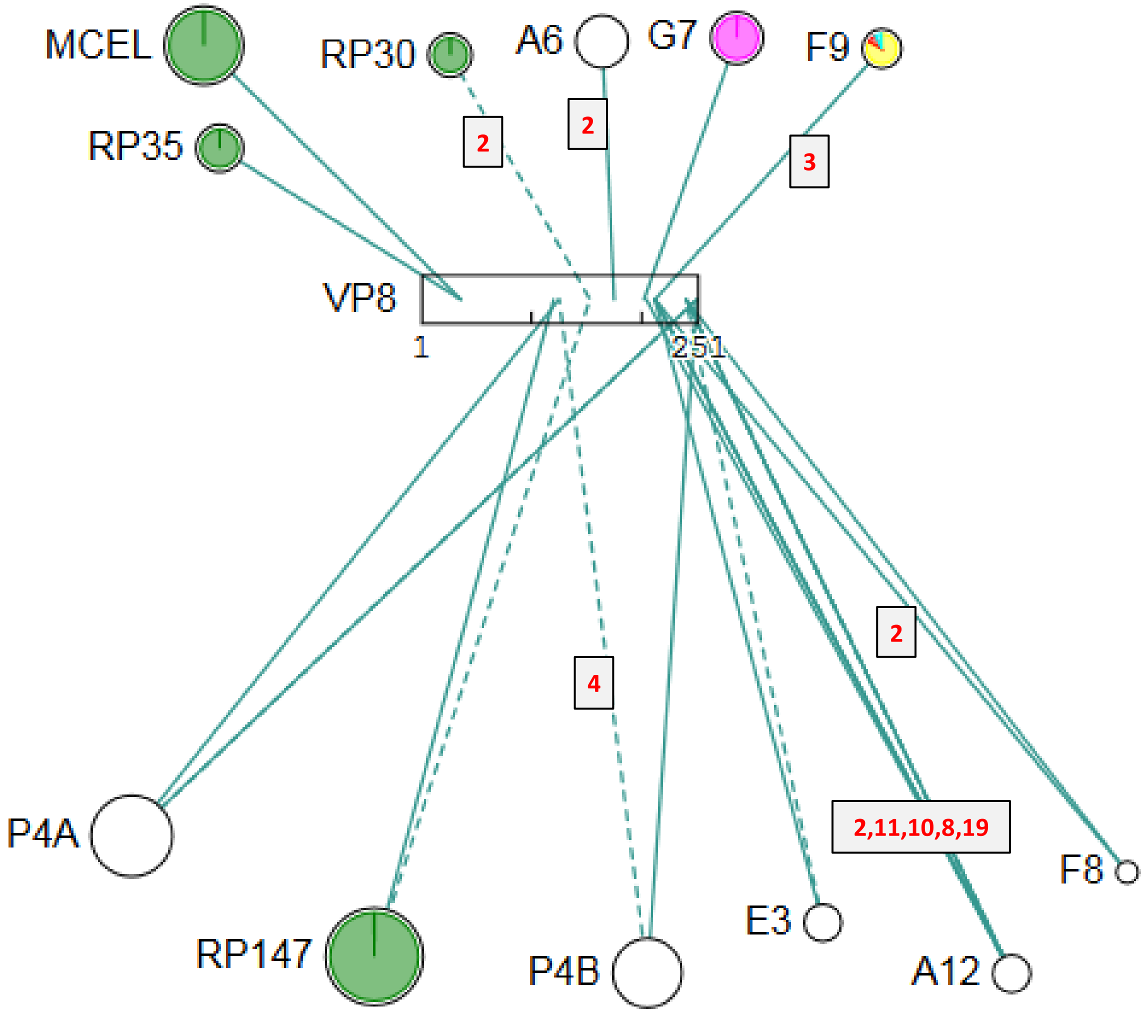

‘Structural’ proteins

A4, p4a, p4b

A4-32/A12-88: 0

A4-33/A16-194: 1.000

A4-33/E6-90: 0

A4-42/A16-194: 1.000

A4-42/F17-74: 0

A4-274/F17-74: 1.000

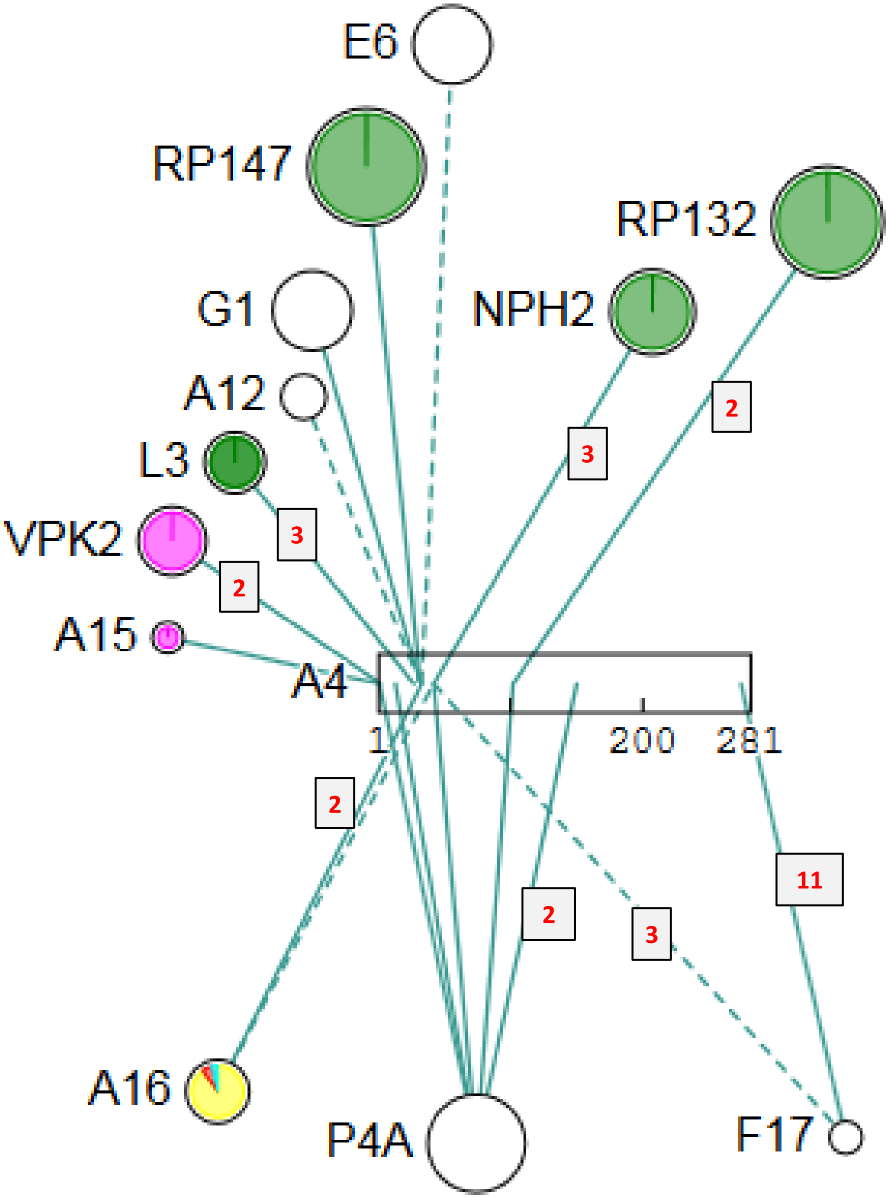

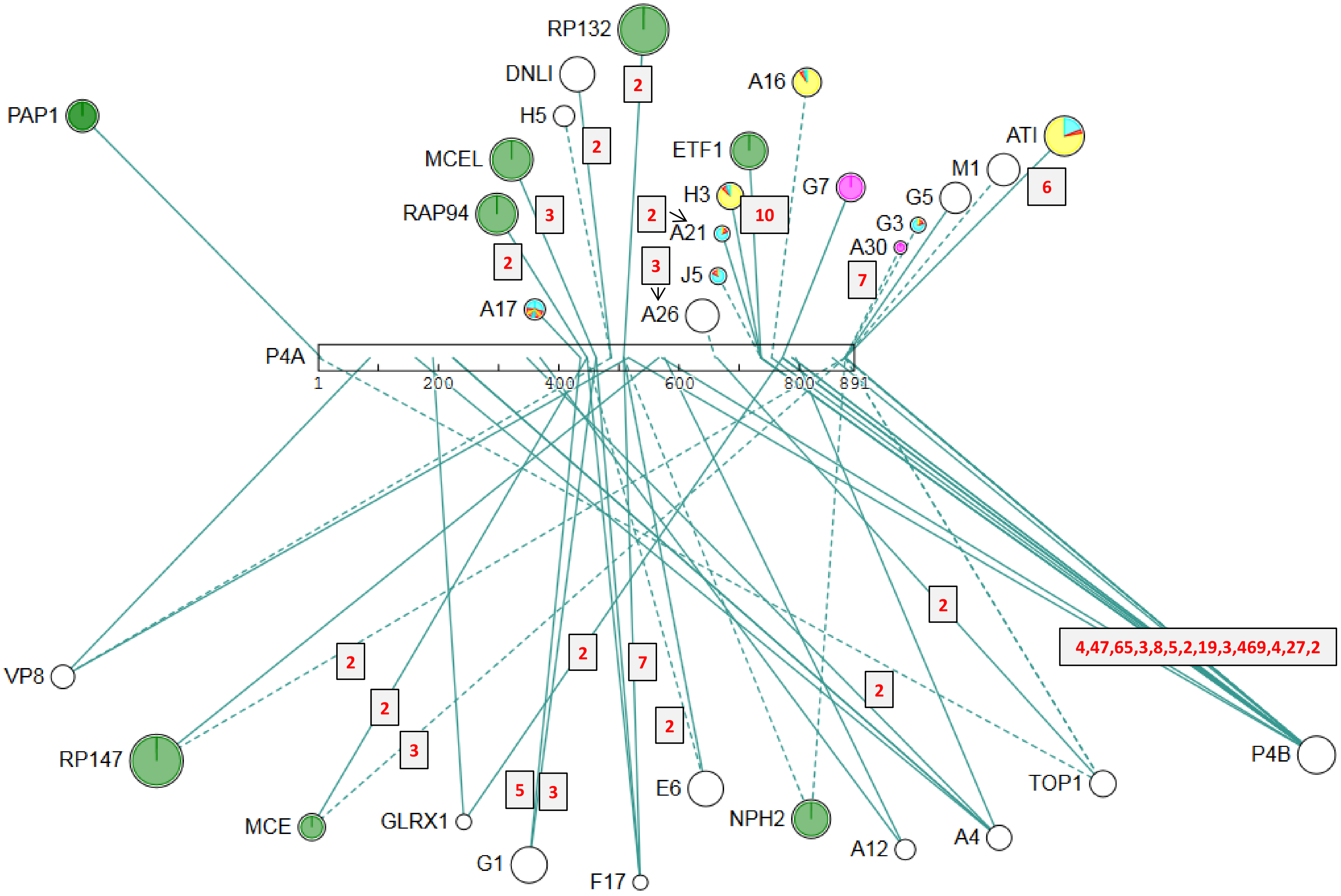

RP132
DNLI
2
A16
PAP1
H5
2
ATI
MCEL
ETF1
M1
6
H3
G7
G5
RAP94
3
2
10
A21
G3
A30
2
3
J5
7
A17
A26
P4A
1
200
400
600
800
891
2
4,47,65,3,8,5,2,19,3,469,4,27,2
VP8
2
2
7
2
2
RP147
3
2
P4B
TOP1
5
3
E6
MCE
GLRX1
NPH2
A4
A12
G1
F17

P4A-1/TOP1-169: 0
P4A-436/G1-96: 0
P4A-447/E6-484: 0
P4A-487/H5-25: 0.500
P4A-487/VP8-250: 0.500
P4A-508/NPH2-627: 0
P4A-508/F17-74: 0
P4A-660/A26-415: 0
P4A-736/J5-92: 0
P4A-736/ETF1-308: 0.333
P4A-736/P4B-393: 1.000
P4A-736/P4B-407: 1.000
P4A-754/A16-133: 0
P4A-788/P4B-567: 0.500
P4A-788/P4B-568: 0
P4A-875/P4B-563: 1.000
P4A-876/A30-49: 0.080
P4A-876/ATI-572: 0
P4A-876/G3-88: 0.167
P4A-876/NPH2-622: 0.167
P4A-876/MCE-321: 0.018
P4A-876/P4B-563: 0.500
P4A-876/P4B-564: 0.982
P4A-876/P4B-567: 1.000
P4A-876/P4B-568: 0

P4A-876/RP147-1029: 0.286
P4A-876/TOP1-167: 0.500
P4A-876/TOP1-207: 0
P4A-876/M1-149: 0

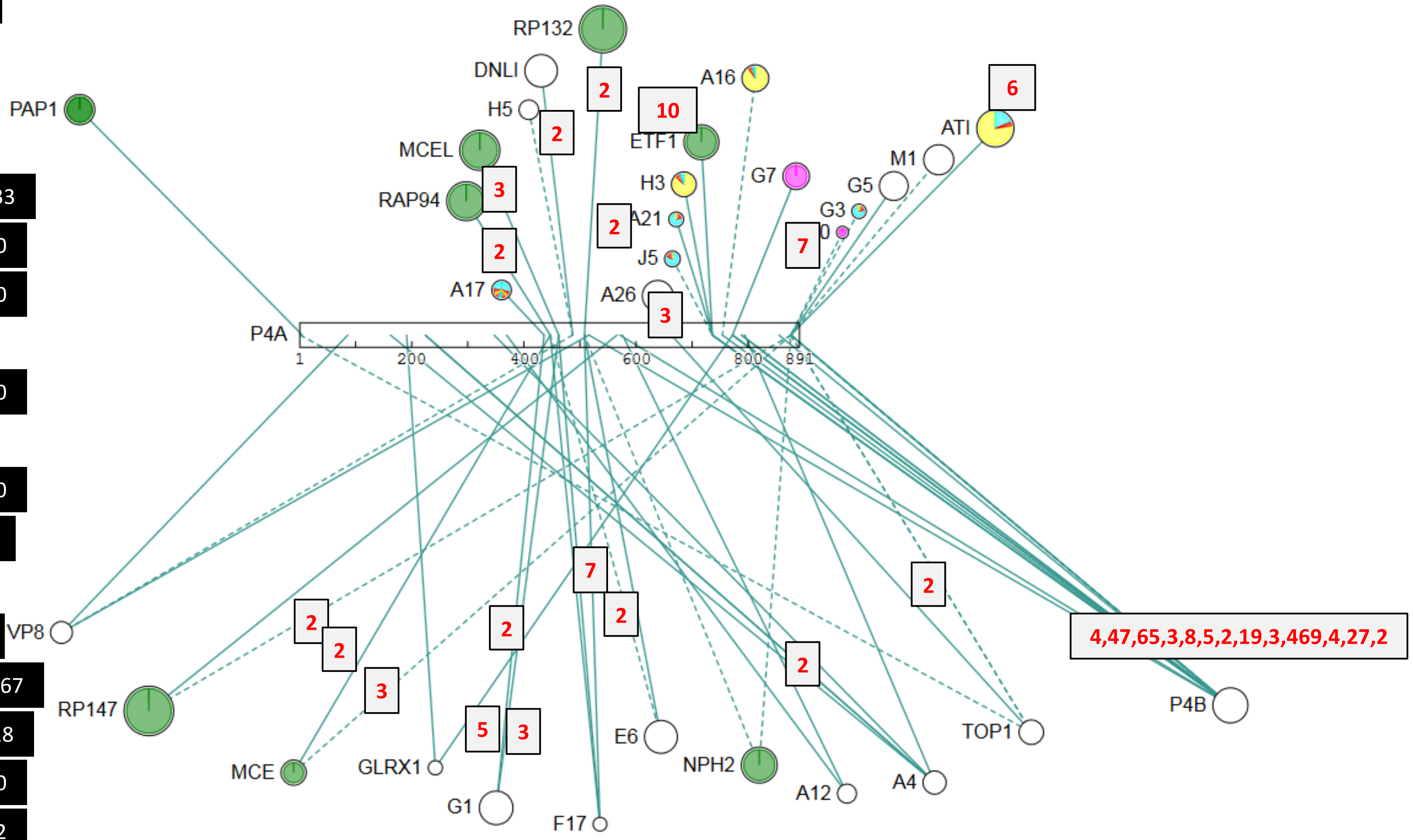

P4a, showing XL with transcriptosome, membrane, structural proteins only

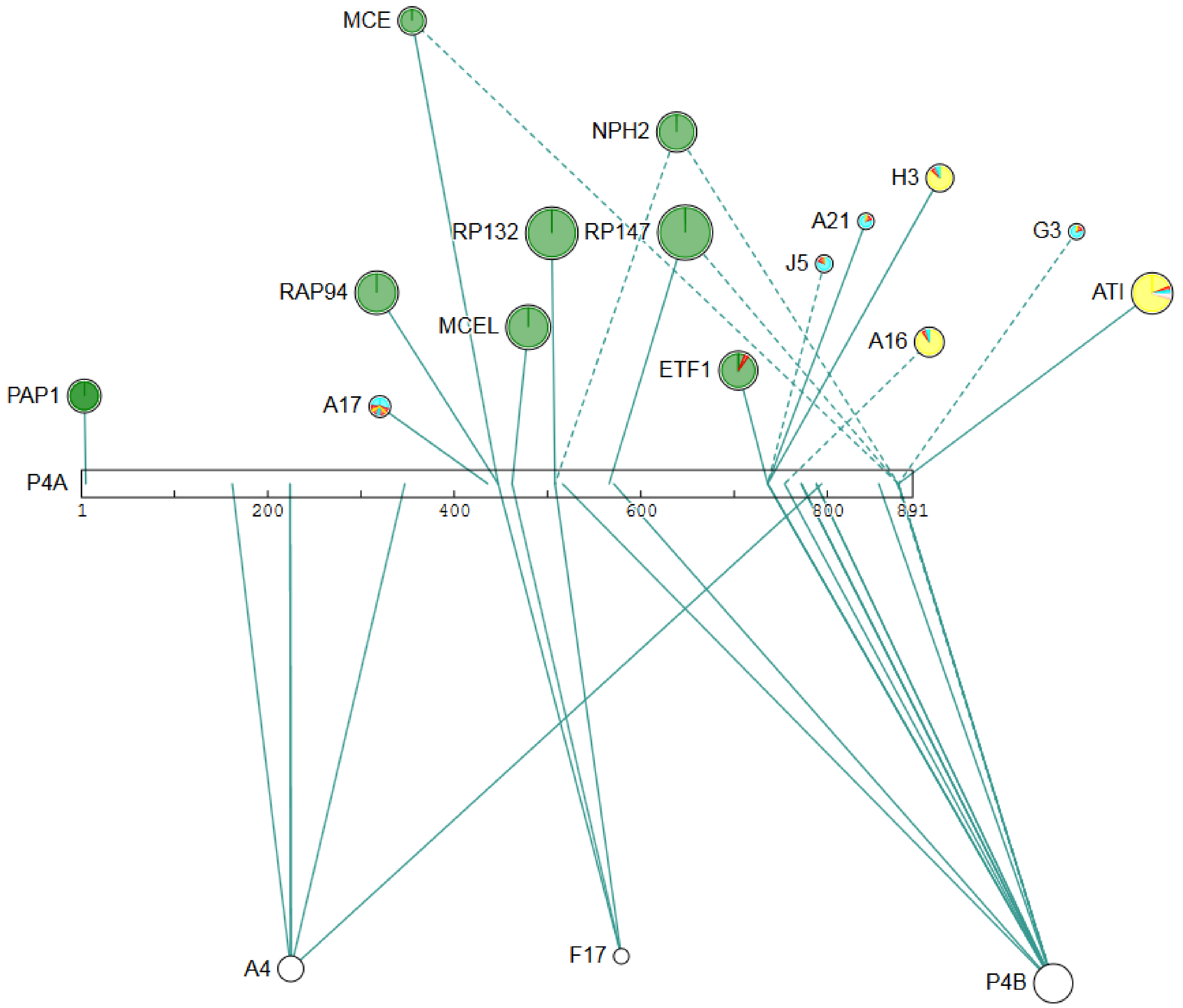

P4a, showing XL with transcriptosome, membrane, structural proteins only:
After removal of three ‘ambig’. XL between transcriptosome proteins and p4a C-terminal residue.

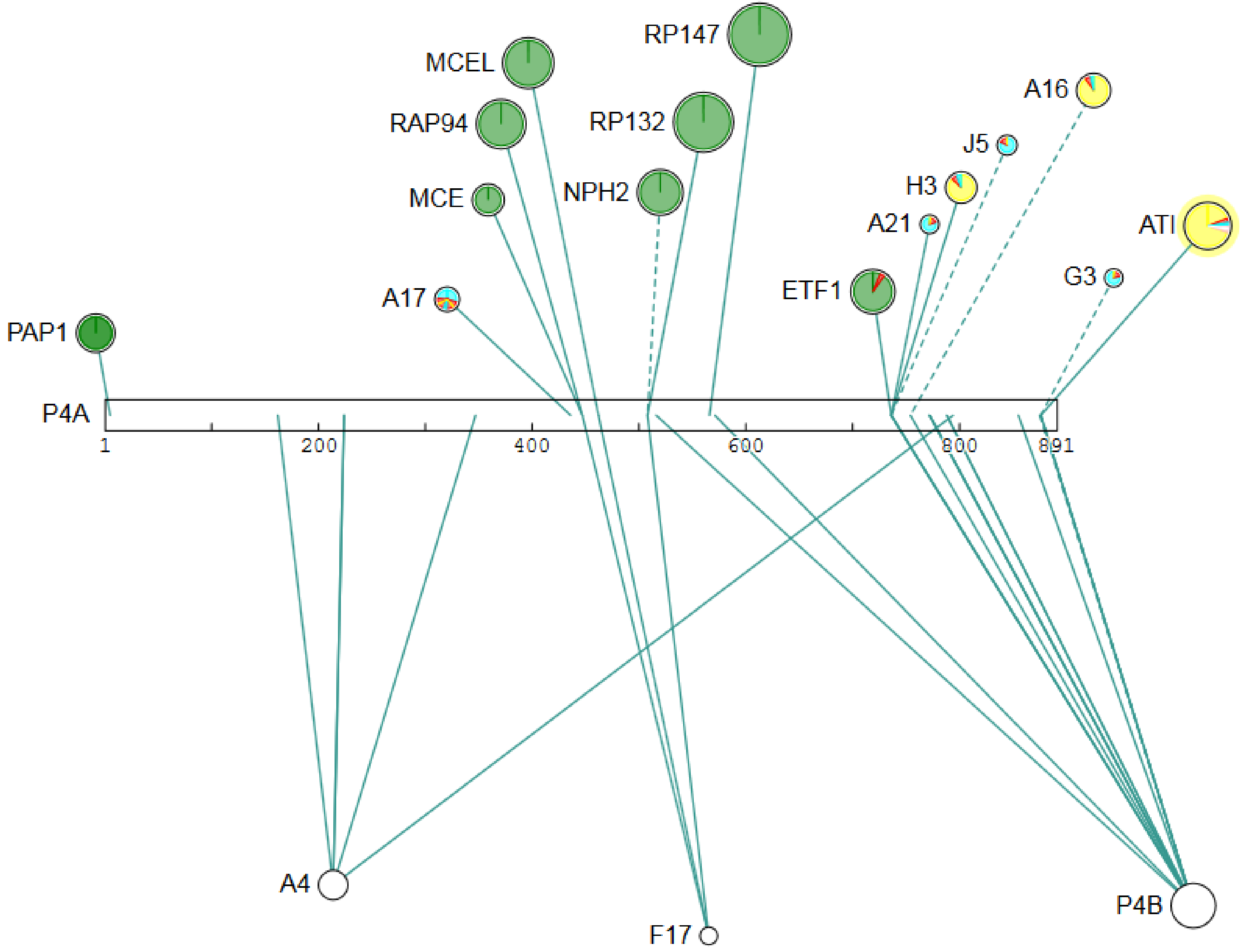

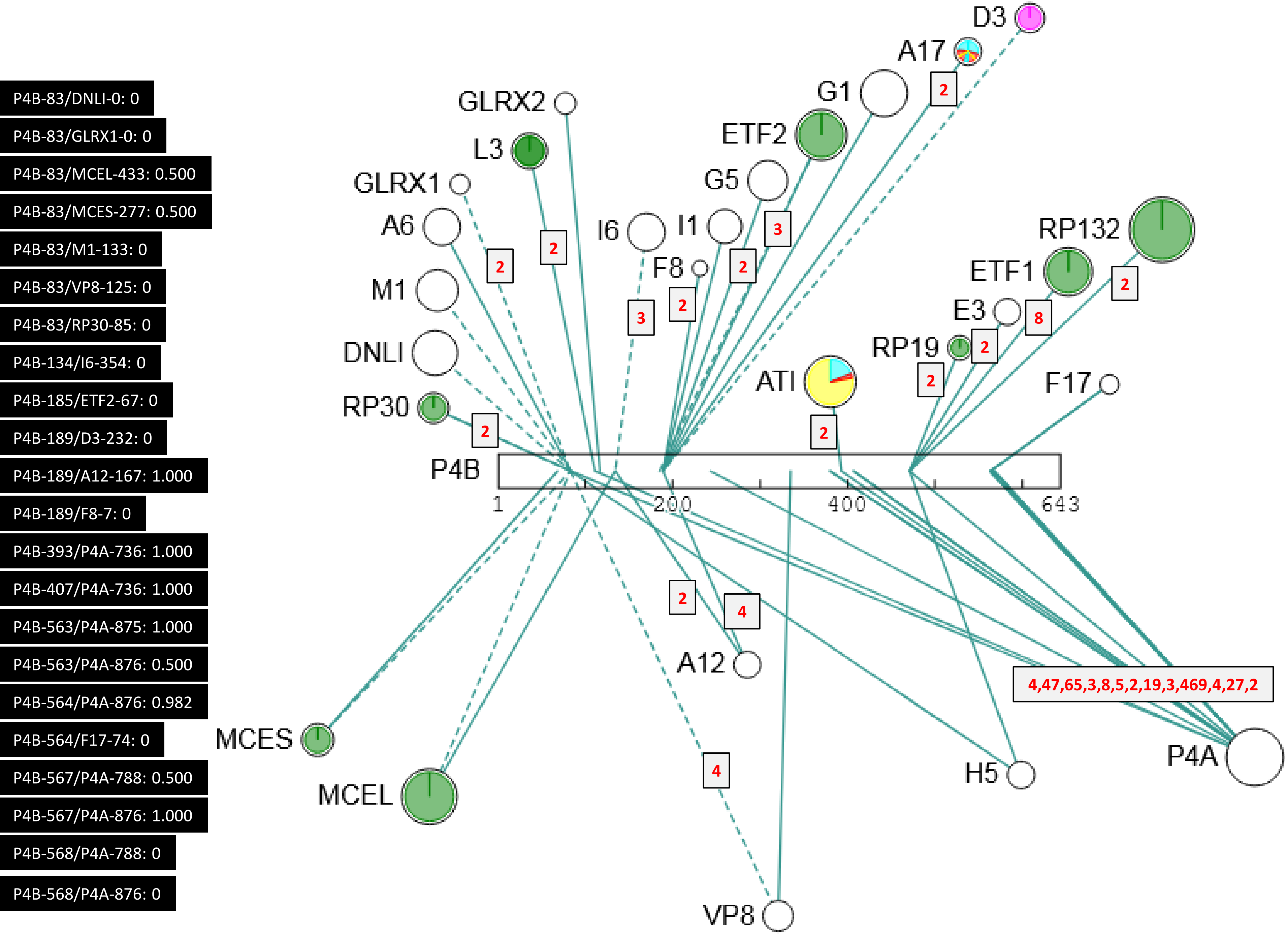
P4B-83/DNLI-0: 0
P4B-83/GLRX1-0: 0
P4B-83/MCEL-433: 0.500
P4B-83/MCES-277: 0.500
P4B-83/M1-133: 0
P4B-83/VP8-125: 0
P4B-83/RP30-85: 0
P4B-134/I6-354: 0
P4B-185/ETF2-67: 0
P4B-189/D3-232: 0
P4B-189/A12-167: 1.000
P4B-189/F8-7: 0
P4B-393/P4A-736: 1.000
P4B-407/P4A-736: 1.000
P4B-563/P4A-875: 1.000
P4B-563/P4A-876: 0.500
P4B-564/P4A-876: 0.982
P4B-564/F17-74: 0
P4B-567/P4A-788: 0.500
P4B-567/P4A-876: 1.000
P4B-568/P4A-788: 0
P4B-568/P4A-876: 0
D3
A17
G1
GLRX2
ETF2
L3
G5
GLRX1
RP132
A6
I6
I1
F8
ETF1
M1
E3
RP19
DNLI
ATI
F17
RP30
P4B
1
200
400
643
A12
4,47,65,3,8,5,2,19,3,469,4,27,2
MCES
P4A
H5
MCEL
VP8

P4b with intramolecular XL.

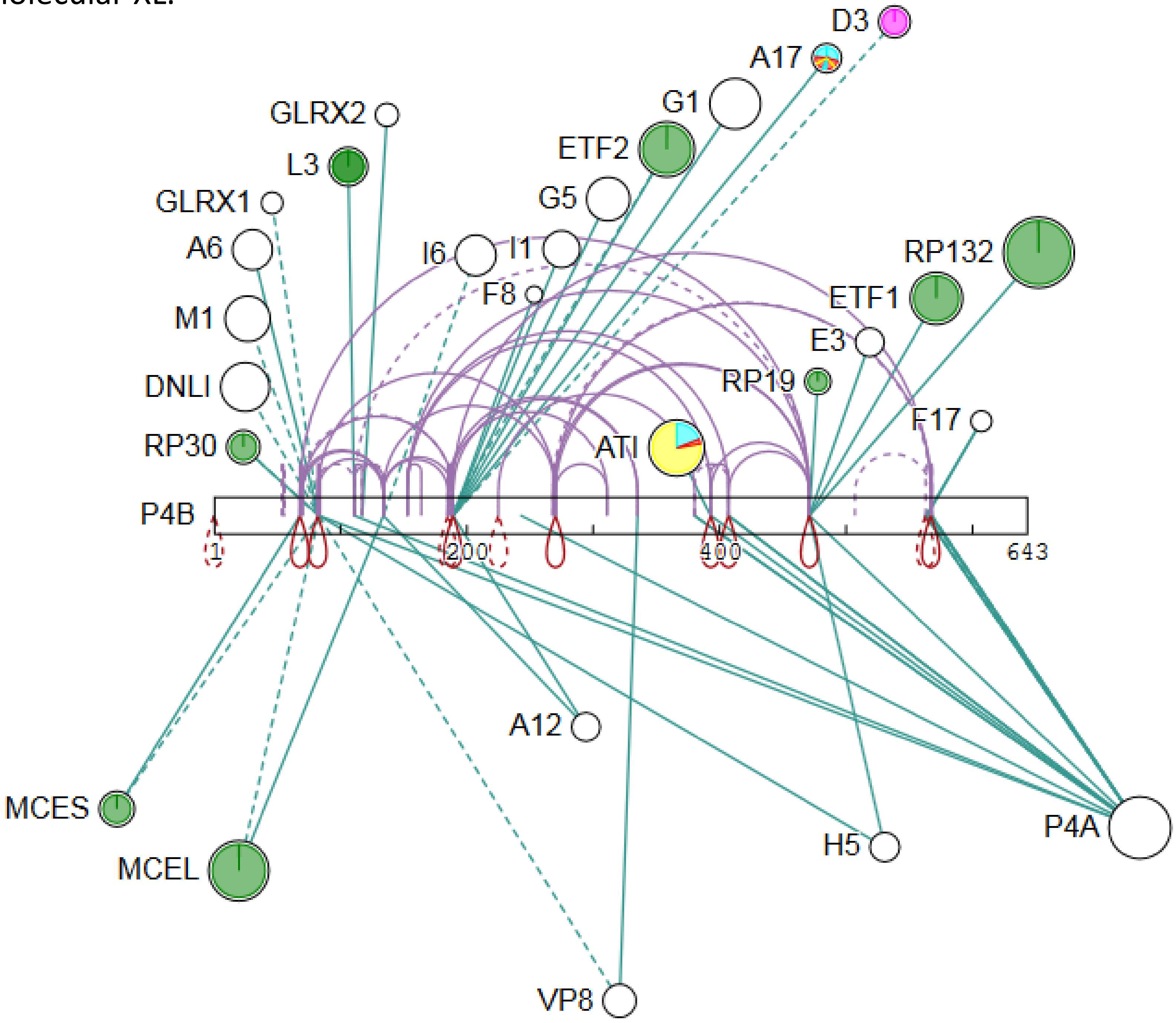

Other enzymes

DUSP, G1, GLRX1, GLRX2, I7, VPK1

DUSP-1/E5-127: 0

DUSP-8/A6-317: 0

DUSP-87/E6-439: 0.500

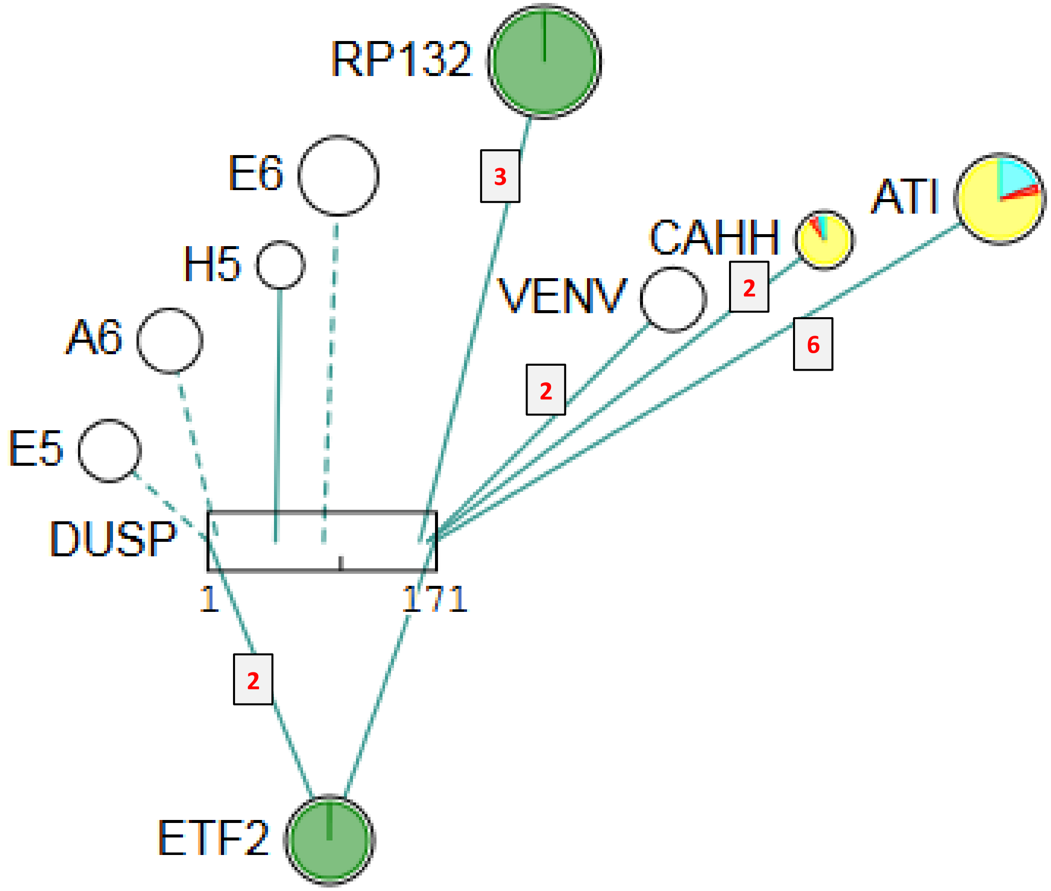

P4A-436/G1-96: 0

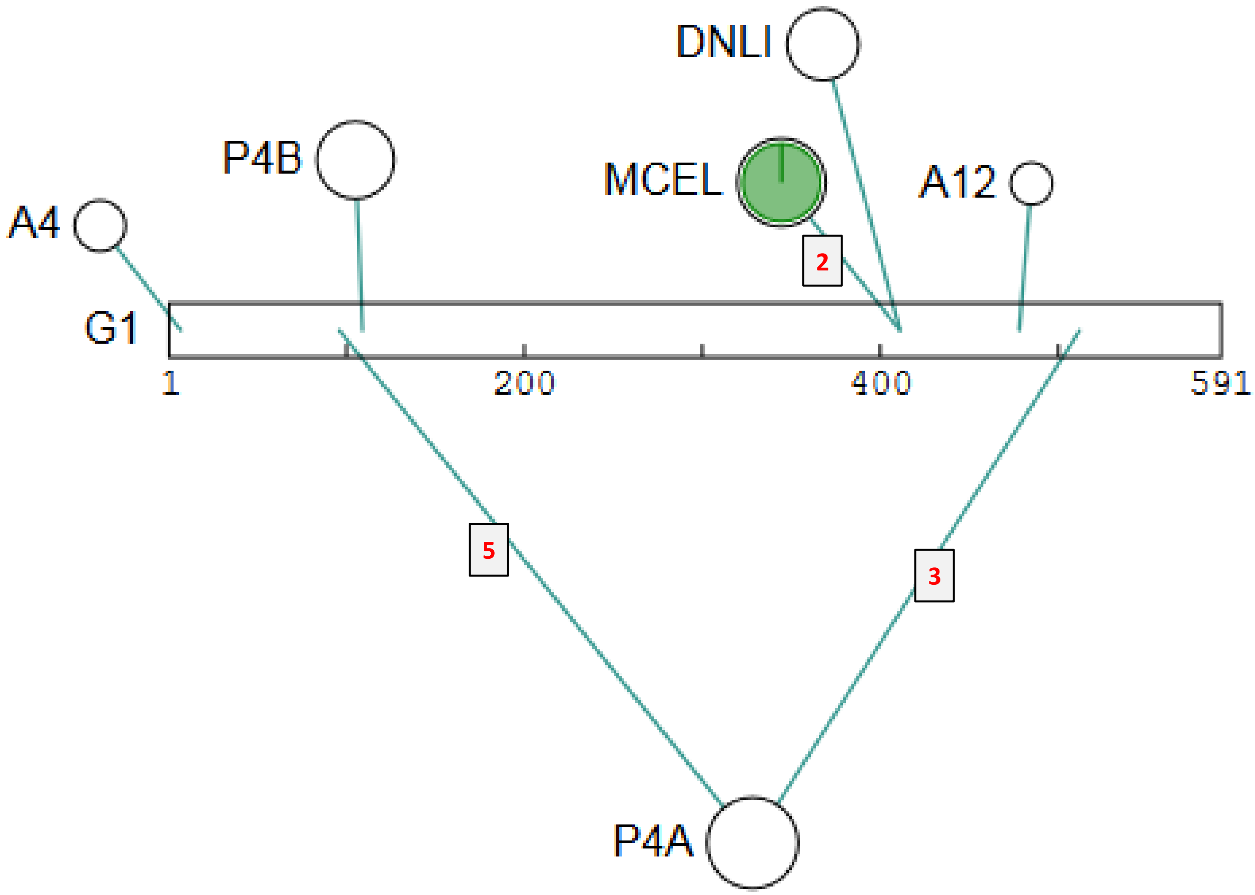

GLRX1-0/P4B-83: 0

GLRX1-1/RAP94-333: 0.500

GLRX1-35/MCES-6: 0

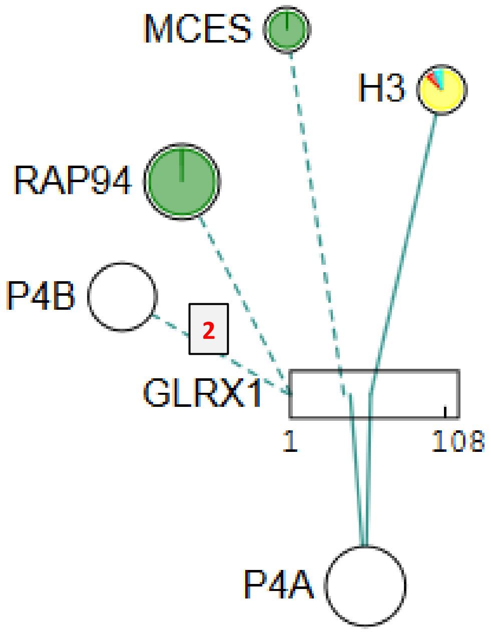

H3-77/GLRX2-27: 0

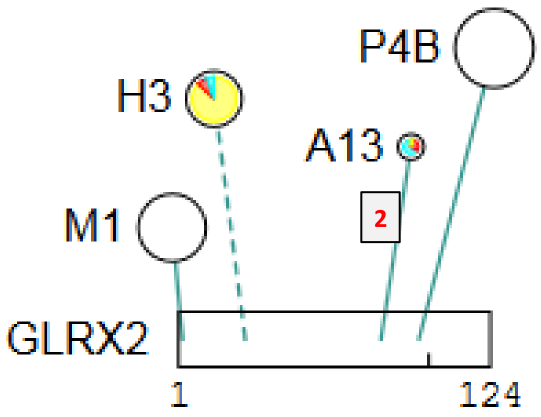

I7-53/RP147-566: 0

I7-90/A13-67: 0

I7-126/A16-149: 0

I7-146/CAHH-14: 0

I7-213/A18-458: 0

I7-364/E6-1: 0

I7-364/H3-147: 0.889

I7-414/TOP1-169: 0.500

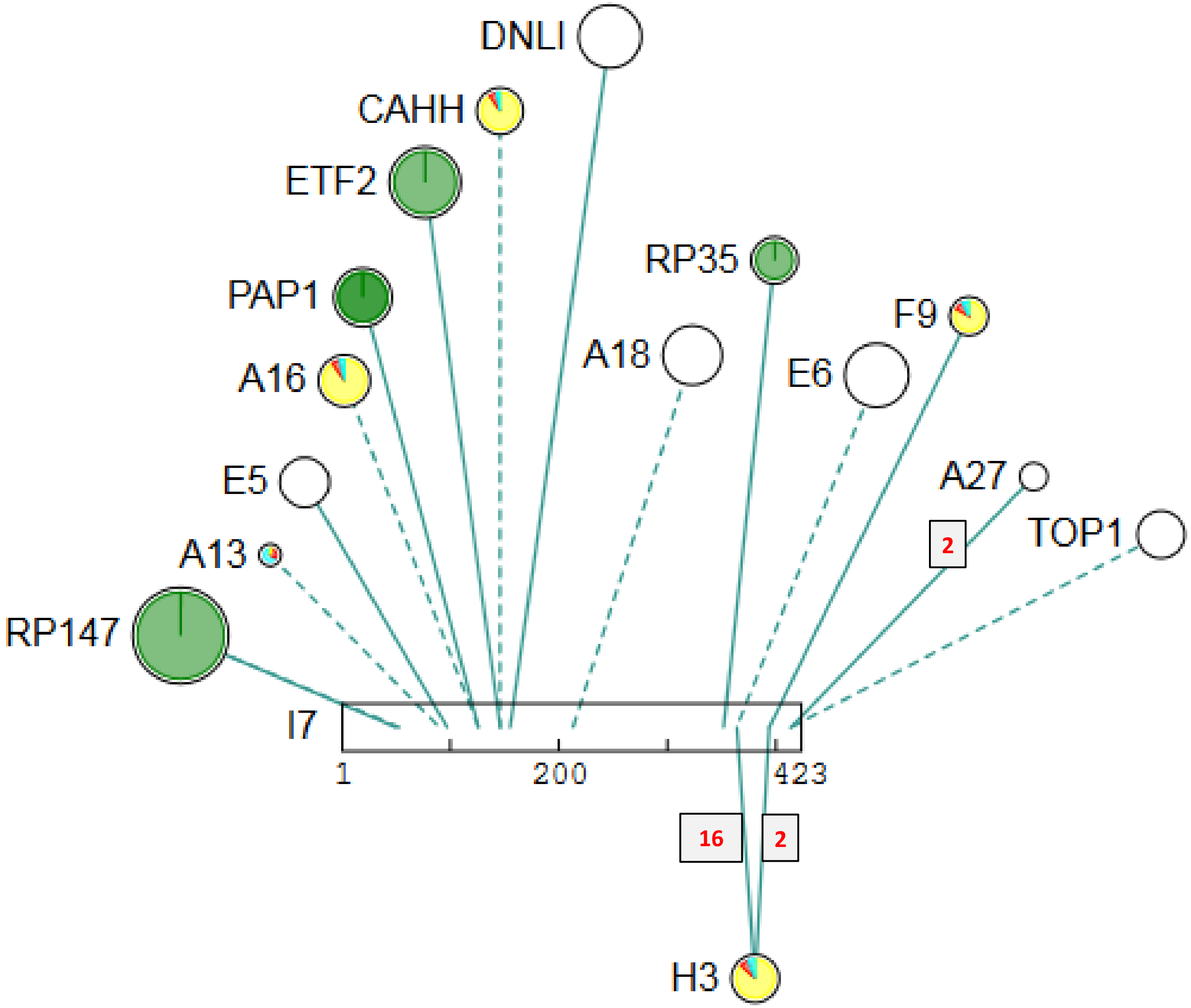

VPK1-71/H3-253: 0

VPK1-121/G9-58: 0

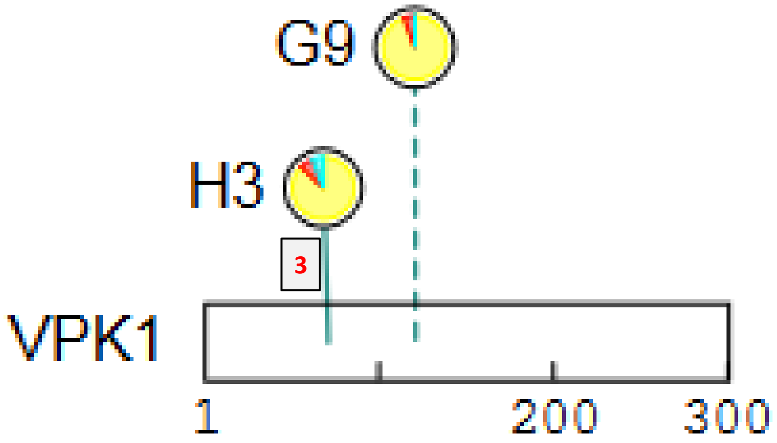

Other proteins

A12, A18, A19, A25(A2.5), A46, E3, E5, E6, E10, E11, F8, F17, M1, N1, PROF, SODL, A46

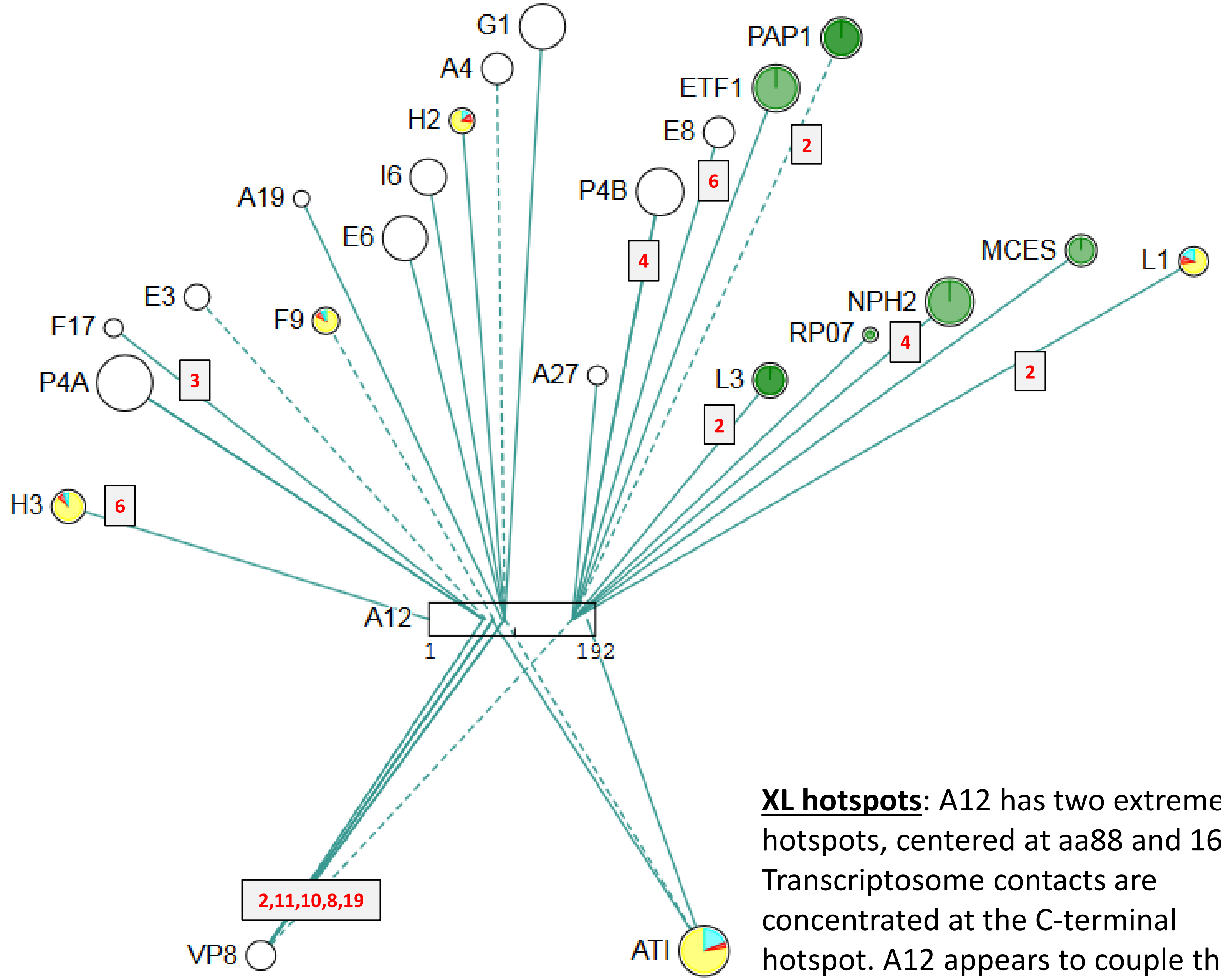

<u>XL hotspots</u>: A12 has two extreme XL hotspots, centered at aa88 and 167. Transcriptosome contacts are concentrated at the C-terminal hotspot. A12 appears to couple the transcription apparatus (C-terminus) with the VP8 interactome (N-terminal region).

A12-63/VP8-240: 0
A12-63/VP8-212: 0
A12-66/E3-109: 0
A12-75/VP8-204: 0
A12-75/VP8-240: 0
A12-75/F9-0: 0
A12-88/A4-32: 0
A12-88/VP8-240: 1.000
A12-88/ATI-572: 0
A12-166/VP8-240: 0
A12-167/L3-18: 0
A12-167/E8-1: 0
A12-167/P4B-189: 1.000
A12-167/PAP1-462: 0

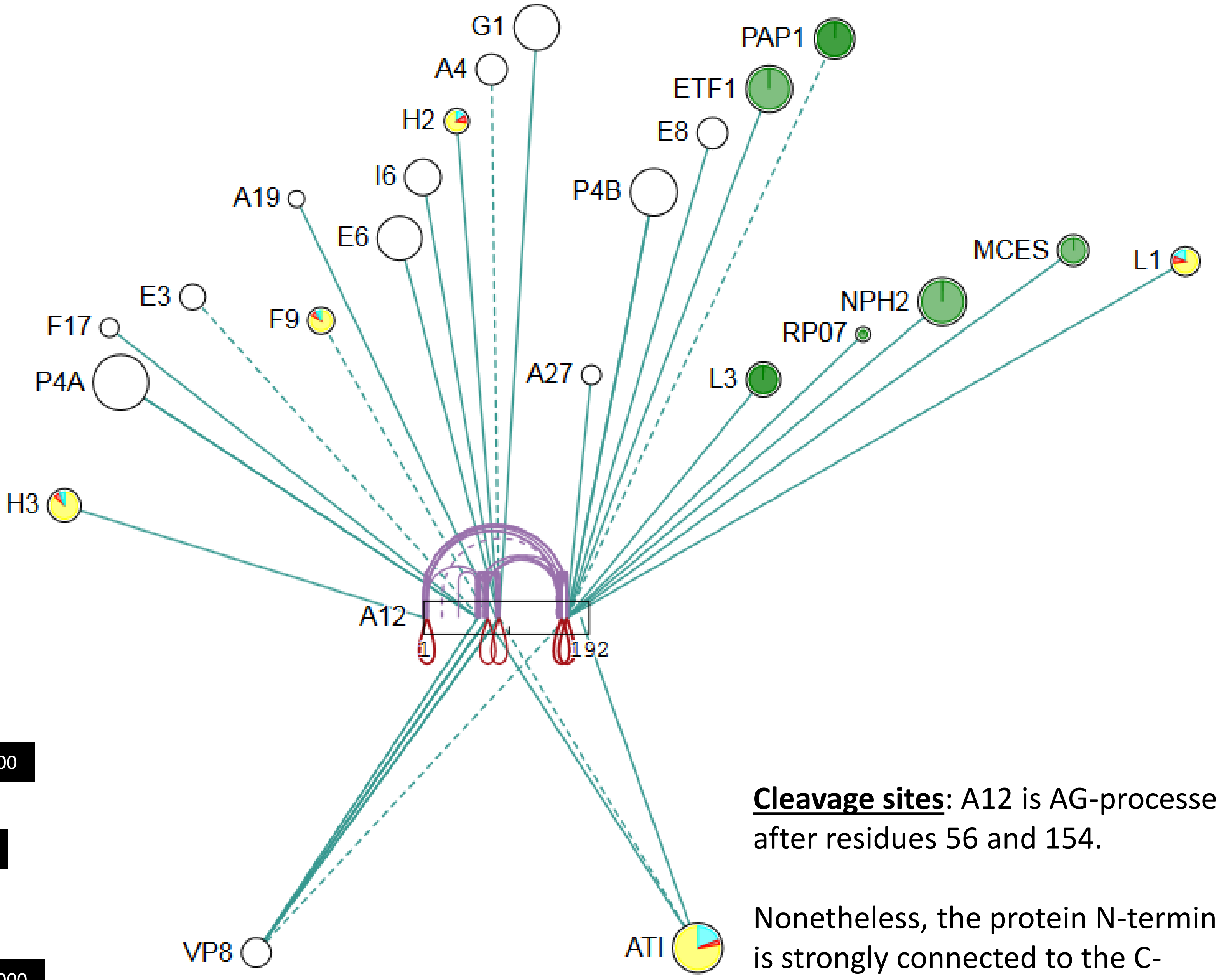

Cleavage sites: A12 is AG-processed after residues 56 and 154.

Nonetheless, the protein N-terminus is strongly connected to the C-terminal hotspot via intra-protein XL.

A18-458/I7-213: 0

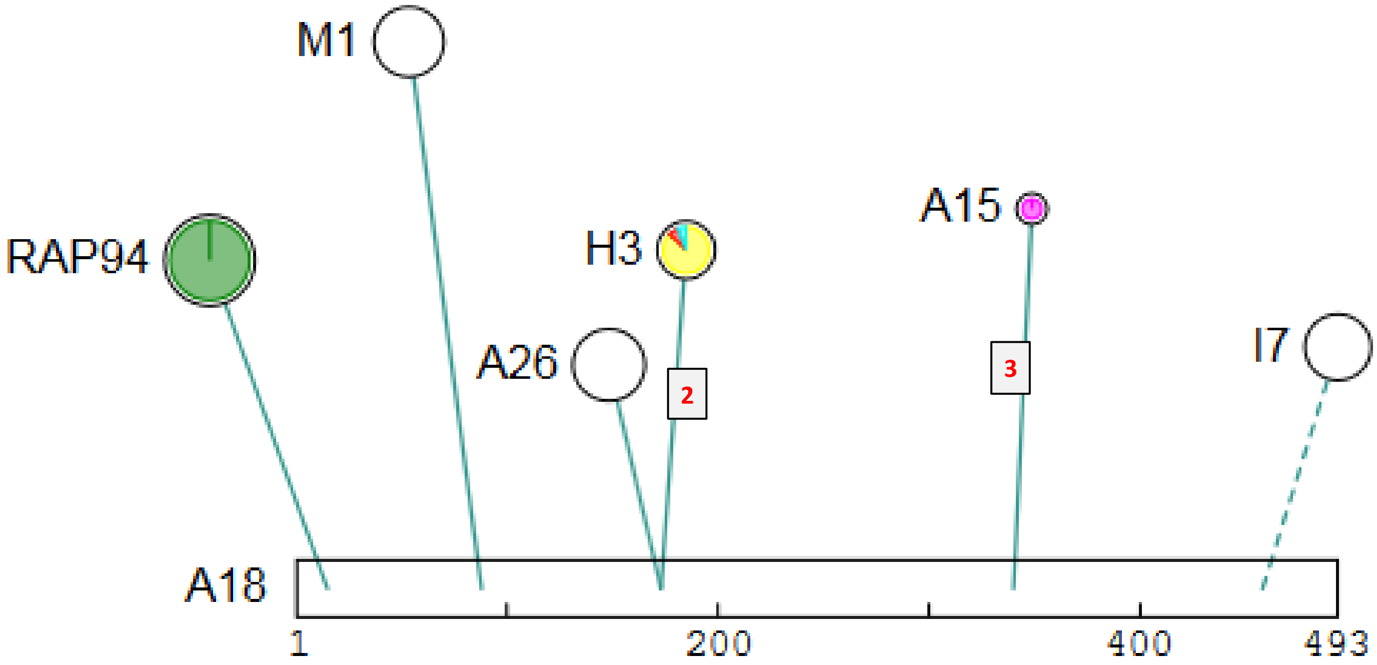

A19-0/DNLI-177: 0.500

A19-1/RP147-566: 0

A19-46/RP132-511: 0

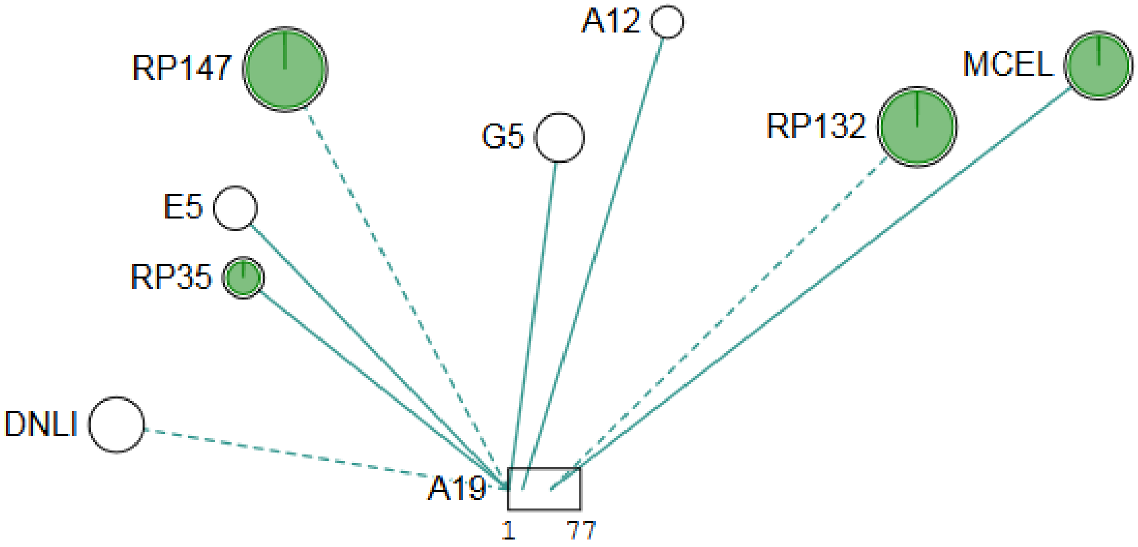

A25-2/F8-7: 0

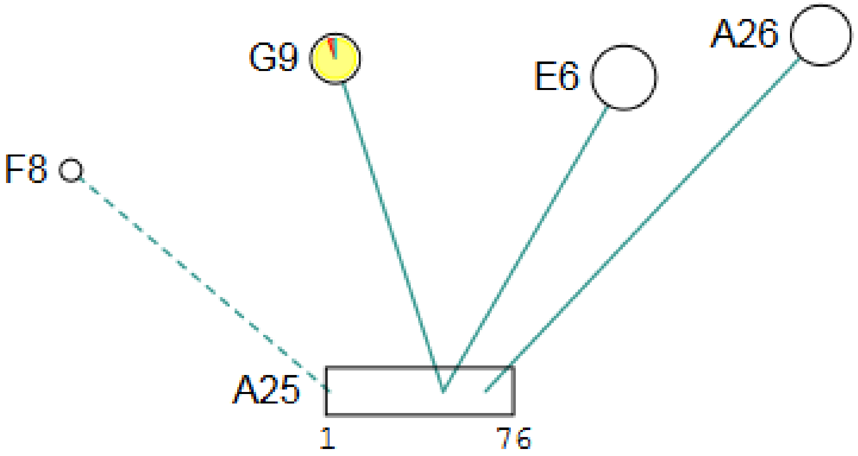

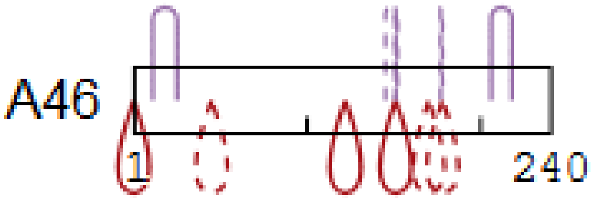
A46
1
240

E3-3/RP30-54: 0.500

E3-40/VP8-240: 0

E3-45/F17-74: 0

E3-109/A12-66: 0

E3-161/E5-223: 1.000

E3-165/E5-223: 1.000

E3-165/G7-345: 0

E3-165/L3-142: 0.500

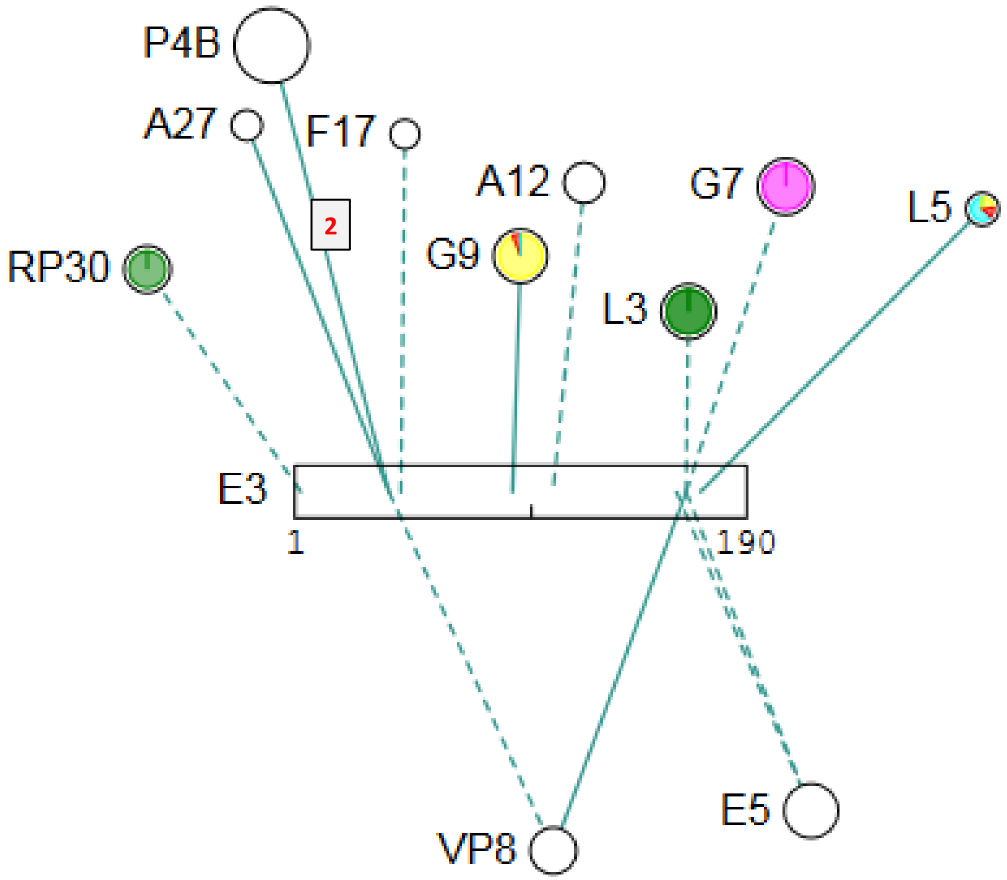

E5-0/A27-57: 0

E5-61/RAP94-206: 0

E5-67/A13-49: 0

E5-127/DUSP-1: 0

E5-223/E3-161: 1.000

E5-223/E3-165: 1.000

E5-231/H3-147: 0

E5-314/RP147-127: 0

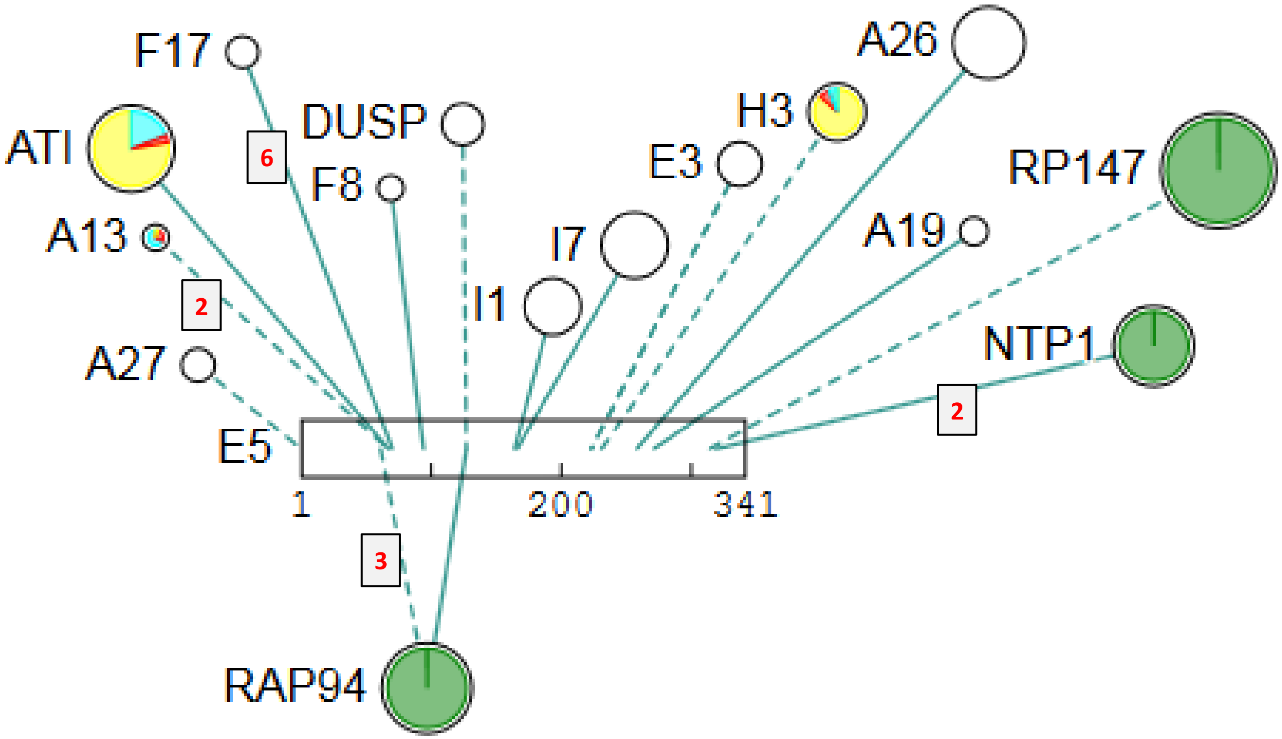

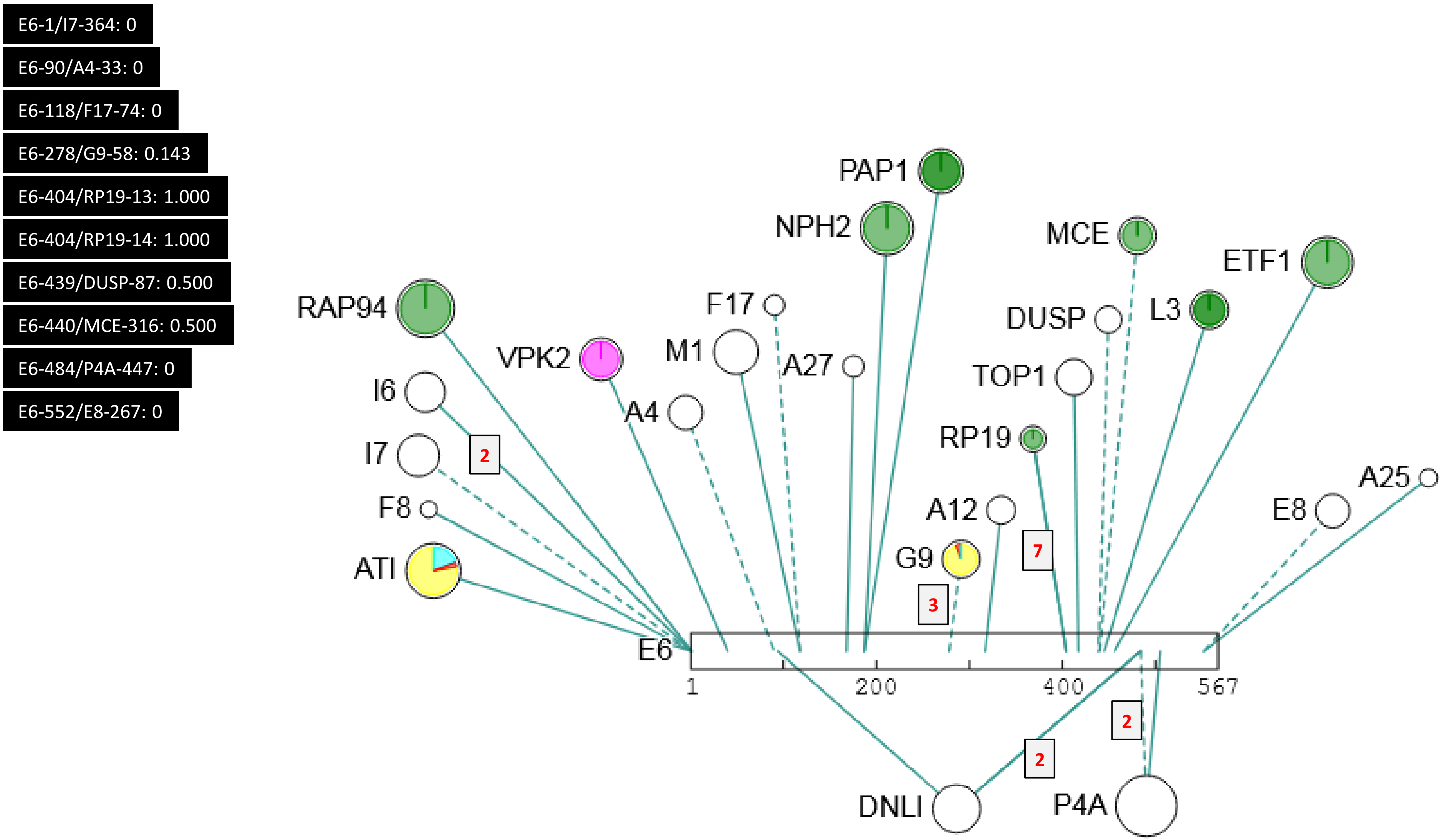
E6-1/I7-364: 0
E6-90/A4-33: 0
E6-118/F17-74: 0
E6-278/G9-58: 0.143
E6-404/RP19-13: 1.000
E6-404/RP19-14: 1.000
E6-439/DUSP-87: 0.500
E6-440/MCE-316: 0.500
E6-484/P4A-447: 0
E6-552/E8-267: 0
PAP1
NPH2
MCE
ETF1
RAP94
F17
DUSP
L3
VPK2
M1
A27
TOP1
I6
A4
RP19
I7
2
A25
F8
A12
E8
7
G9
ATI
3
E6
1
200
400
567
2
2
DNLI
P4A

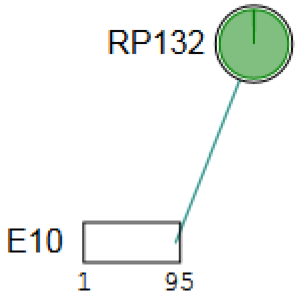
RP132
E10
1
95

E11-56/H5-1: 0

E11-62/NTP1-32: 0

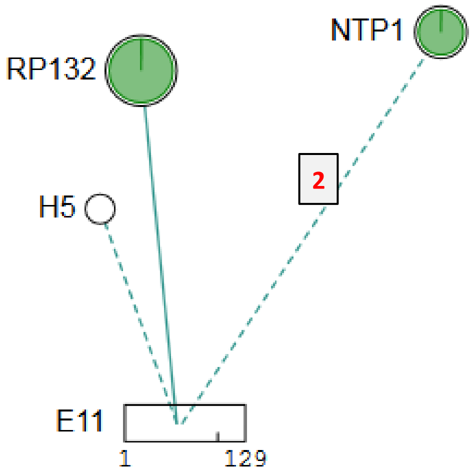

F8-1/A17-180: 0

F8-7/A25-2: 0

F8-7/P4B-189: 0

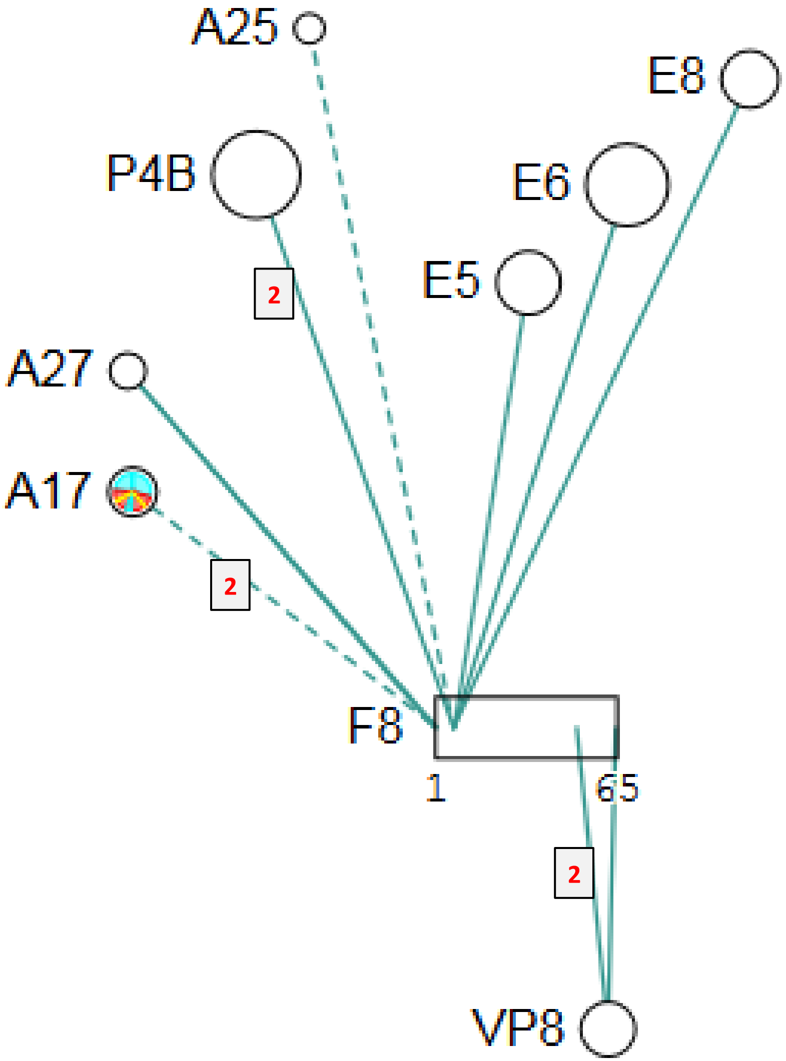

F17-17/RP147-37: 0

F17-50/ETF1-252: 0

F17-74/A4-42: 0

F17-74/A4-274: 1.000

F17-74/P4A-508: 0

F17-74/E6-118: 0

F17-74/J1-74: 0

F17-74/L3-22: 0

F17-74/NTP1-569: 0

F17-74/P4B-564: 0

F17-74/E3-45: 0

F17-74/M1-277: 0

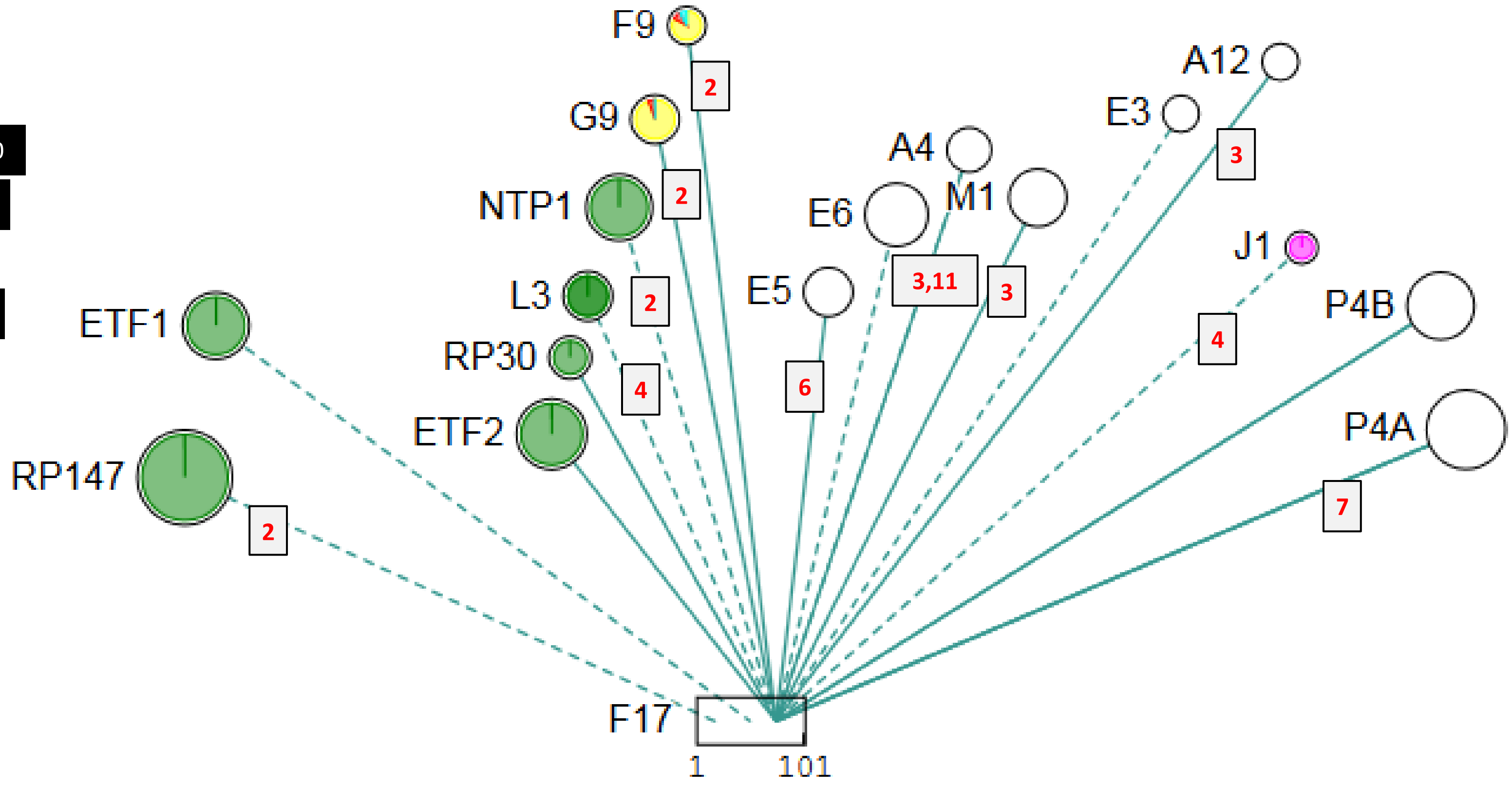

M1-133/P4B-83: 0

M1-149/P4A-876: 0

M1-277/F17-74: 0

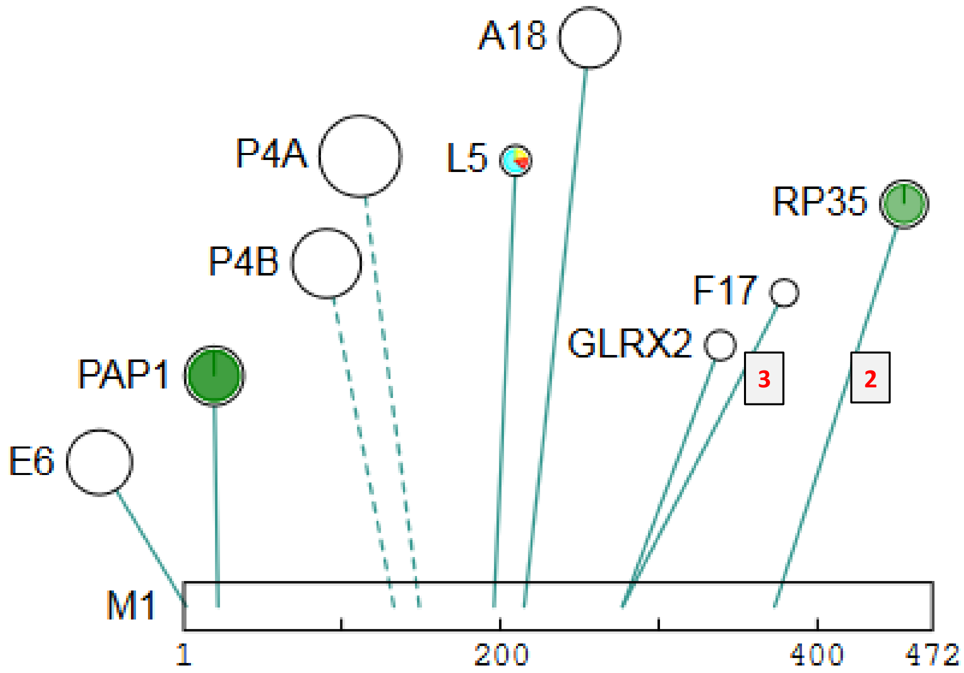

RP147-951/N1-78: 0

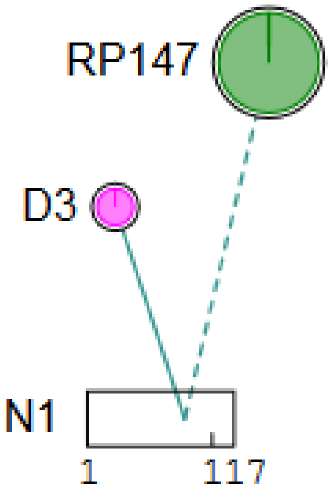

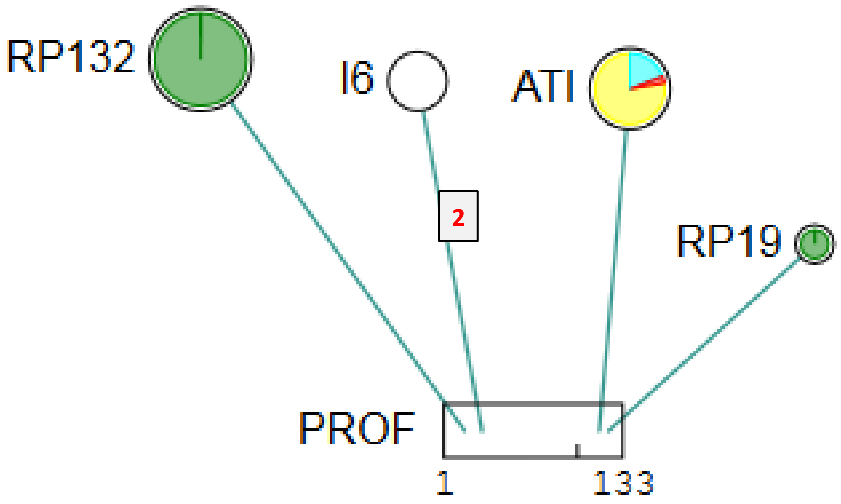
RP132
I6
ATI
RP19
2
PROF
1
133

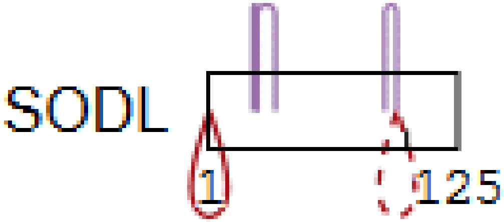
SODL
1
125
