# Supplementary material for: The Vaccinia virion: Filling the gap between atomic and ultrastructure
Source: PLoS Pathog. 2019 Jan 7;15(1):e1007508. doi: 10.1371/journal.ppat.1007508 (PMC6336343; doi:10.1371/journal.ppat.1007508)
Supplement: S2 Fig — (a) Intra-protein crosslinks within all TM proteins. Yellow, red, cyan fill: ‘Outside’, TM, ‘Inside’ domains, respectively (see legend to Fig 8). Mauve loops (above protein): Crosslinked peptides from the same protein sequence. Although depicted as intra-molecular (‘intra-protein’) XL, formally, any of them could, instead, span homomultimer subunits. Red loops (beneath protein): Identical crosslinking site from the same protein for both members of the crosslinked peptide pair (a bona fide indicator of homomultimerization). Vaccinia ATI protein is included in the TM protein group due to its predicted possession of a TM domain with 80% probability (albeit this was a lower probability than for the other TM proteins, see below). With few exceptions, intra-protein crosslinks followed the discrete domains predicted by the program TMHMM [68], ie. did not show membrane-spanning ‘Inside’-to-’Outside’ XL. Proteins containing apparent exceptions to this are indicated with a red star (*) at the C-terminus (right-hand column of proteins). Although lipid bilayers (~50 Å in thickness [141]) are substantially beyond the 11 Å span of crosslinkers such as DSS, a number of experiments involved brief NP40+TCEP pre-treatment with the likelihood of liberating proteins from the virion envelope. Nonetheless, TM proteins can retain rigidity and structural integrity in their (bundled) bilayer-spanning portions even in the absence of a lipid barrier [142, 143], and TM domains are typically impoverished in charged residues such as lysines, which comprised the crosslinking targets in most of our experiments. The paucity of XL spanning predicted TM domains provided additional validation for the XL dataset as a whole. No intra-protein XL were detected for TM proteins F14.5 and I5, which are therefore not depicted. (b) TMHMM prediction of TM domain(s) in Vaccinia protein ATI. In contrast to all other TM proteins, whose TM domain prediction posterior probability (red) approached 1.0 (100%), for ATI [file ppat.1007508.s002.pdf]

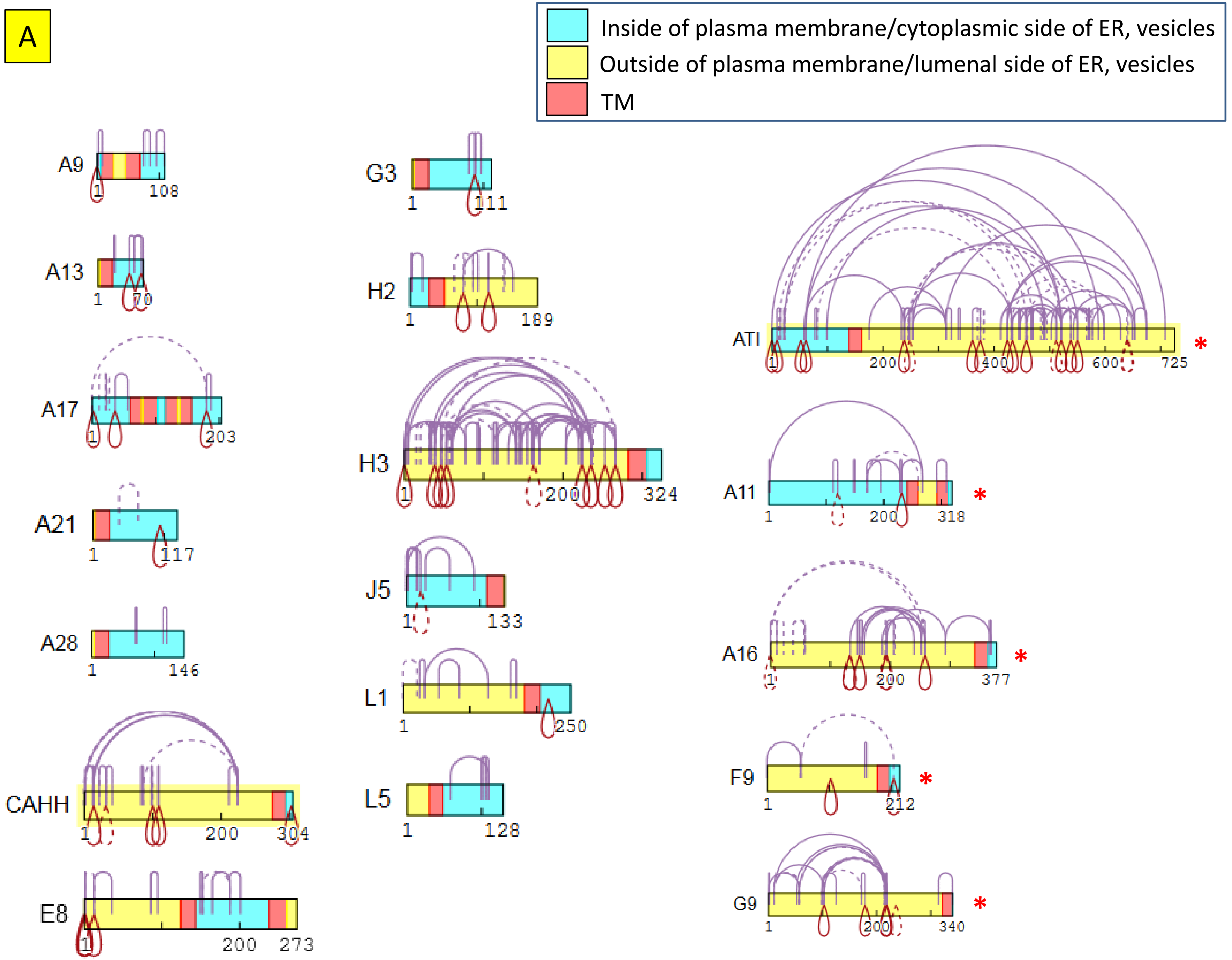

A
Inside of plasma membrane/cytoplasmic side of ER, vesicles
Outside of plasma membrane/lumenal side of ER, vesicles
TM
A9
1
108
A13
1
70
A17
1
203
A21
1
117
A28
1
146
CAHH
1
200
304
E8
1
200
273
G3
1
111
H2
1
189
H3
1
200
324
J5
1
133
L1
1
250
L5
1
128
ATI
1
200
400
600
725
*
A11
1
200
318
*
A16
1
200
377
*
F9
1
212
*
G9
1
200
340
*

```
# WEBSEQUENCE Length: 725
# WEBSEQUENCE Number of predicted TMHs:  1
# WEBSEQUENCE Exp number of AAs in TMHs: 26.15002
# WEBSEQUENCE Exp number, first 60 AAs:  0
# WEBSEQUENCE Total prob of N-in:        0.77264
WEBSEQUENCE     TMHMM2.0        inside       1   138
WEBSEQUENCE     TMHMM2.0        TMhelix    139   161
WEBSEQUENCE     TMHMM2.0        outside    162   725
```

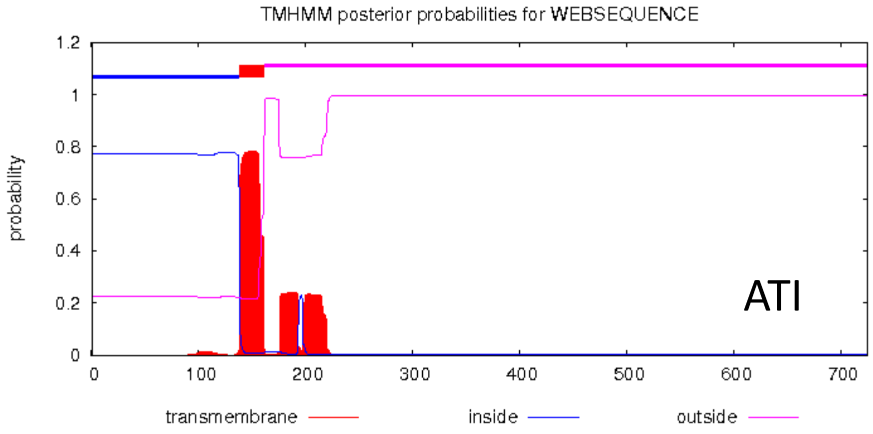

B

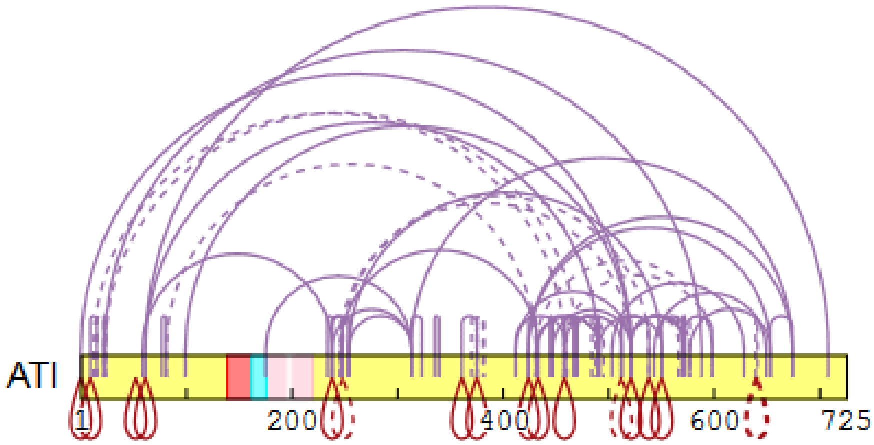

C
