# Supplementary figures and images for: The Vaccinia virion: Filling the gap between atomic and ultrastructure
Source: PLoS Pathog. 2019 Jan 7;15(1):e1007508. doi: 10.1371/journal.ppat.1007508 (PMC6336343; doi:10.1371/journal.ppat.1007508)

S3 Fig

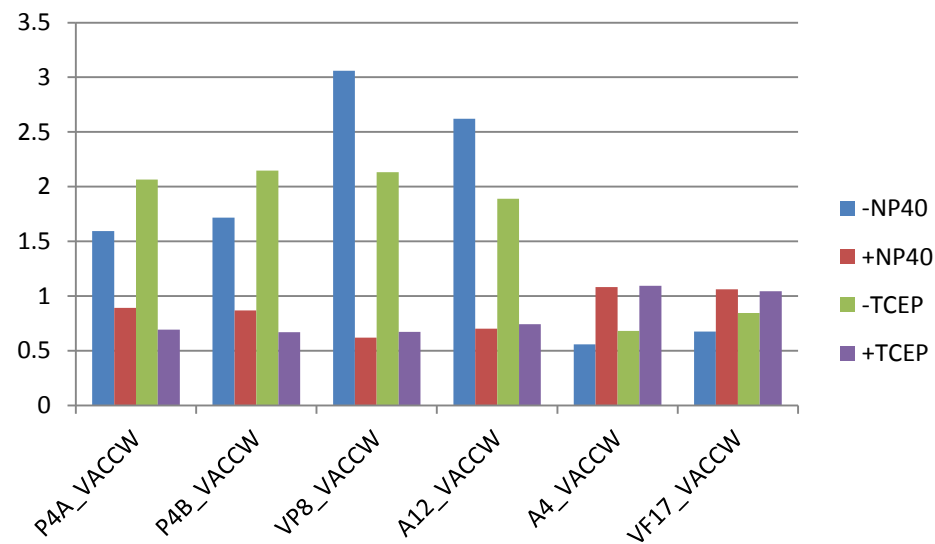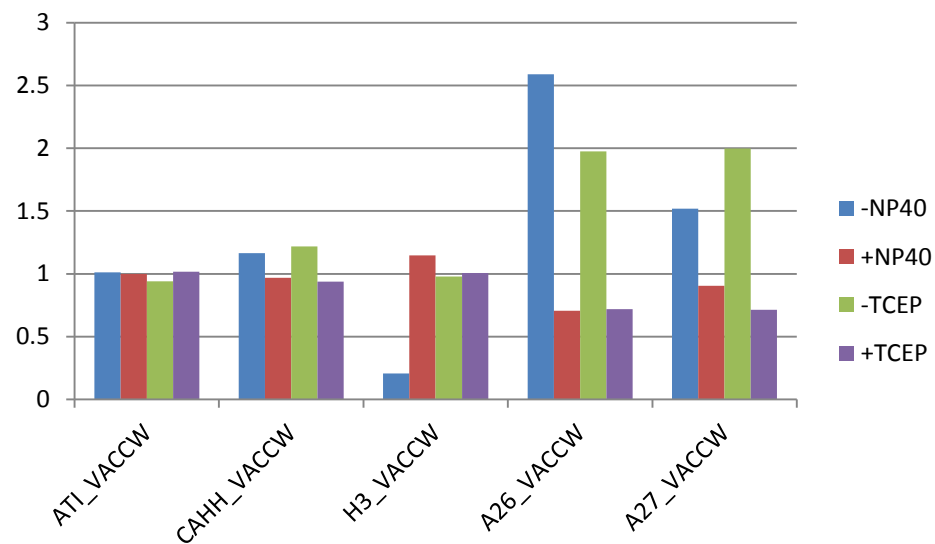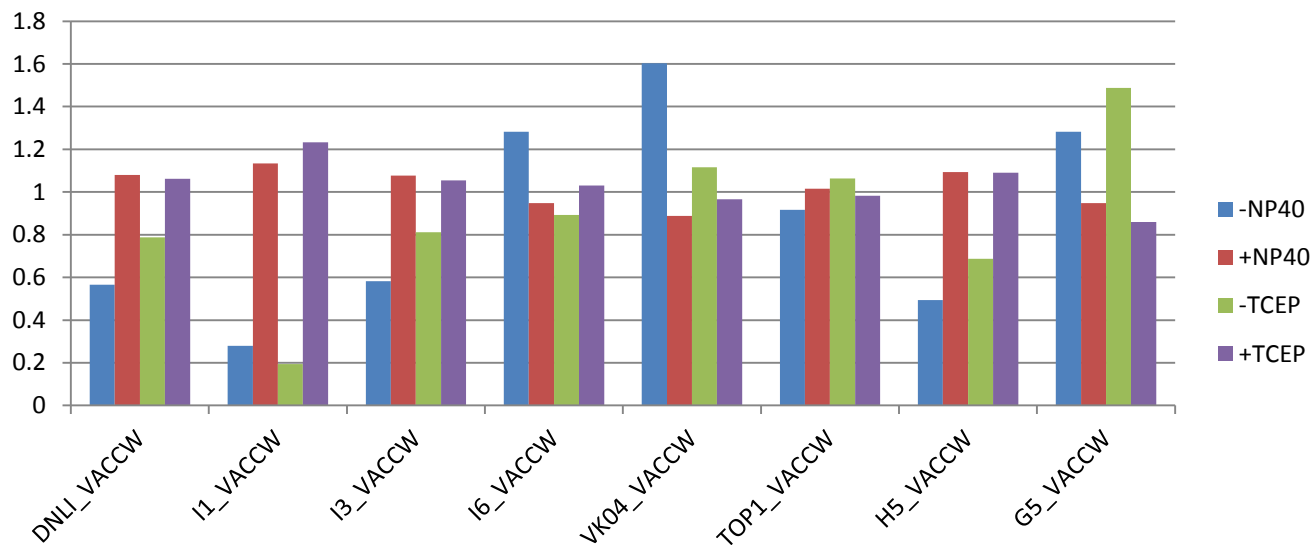

Supplement: S3 Fig — Briefly, Y infers the number of XL detects for an accession in the presence (+) or absence (-) of virion pre-treatment reagents NP40 and TCEP, as a proportion of total XL detects for the accession, and after each of the four resulting values were then normalized for the different number of XL detects per accession for each treatment condition. Y > 1 and Y < 1 infer that the stated pre-treatment tends to stimulate or suppress crosslinking, respectively, with respect to an average protein in the virion crosslinkome. Actual pre-treatment conditions (Fig 1) were deconstructed to yield the bars shown: The -NP40/-TCEP (‘None’) condition was binned as both–NP40 and–TCEP, the +NP40 alone condition was binned as both +NP40 and–TCEP, and the +NP40/+TCEP condition was binned as both +NP40 and +TCEP. Values +TCEP alone are therefore inferred. Upper-left, upper-right and lower panels: Key structural, membrane and ‘DNA group’ proteins, respectively. (PDF) [file ppat.1007508.s003.pdf]
