# Supplementary material for: The Vaccinia virion: Filling the gap between atomic and ultrastructure
Source: PLoS Pathog. 2019 Jan 7;15(1):e1007508. doi: 10.1371/journal.ppat.1007508 (PMC6336343; doi:10.1371/journal.ppat.1007508)
Supplement: S1 Text — Table A. Proteins in the XL search database in relation to their virion packaging status, and for which XL were not detected. Table B. Partial and complete X-ray crystallographic structures covering the crosslinked portions of proteins in the XL search database. Table C. Numbers of crosslinking ions detected for the three predicted fragments of protein p4a and two predicted fragments of protein p4b. Table D. EFC interactions noted previously and in the current study. Table E. DFscoring inter-protein XL in the dataset, ordered by descending DFscore. Table F. Matrix of all detected interactions between transcriptosome proteins (listed on row 1) and membrane (TM plus membrane-associated) proteins (listed in column 1). Table G. Matrix of all detected transcriptosome-transcriptosome protein interactions. Table H. Crosslinked peptides in the dataset spanning AG| sites known to be cleaved during virion maturation. Table I. Parameters used with crosslink search engines. (PDF) [file ppat.1007508.s005.pdf]

Supplementary Tables A - I

Table A. The XL search engine database (XLDB) was tailored carefully to reflect the expected Vaccinia virion proteome. It was based on the 58-member 'consistently packaged' portion of the published virion quantitative proteome histograms (the first 58 data rows in Table S7 of ref. [1]), supplemented with the following proteins considered potentially packaged: 11 of the 16 proteins from the 'inconsistently packaged' portion listed in Table S7 of ref. [1] (the remaining five are highlighted cyan in table rows 21 – 25, below); 11 proteins from outside the 'packaged' region (rows 10 – 20 highlighted green in the table below) and 6 proteins never detected in the published quantitative proteomes (rows 1 – 6, highlighted yellow in the table below). The resulting XLDB had 86 entries.

Rows 7 – 9 of the table below show proteins from the 'packaged' portion in Table S7 of ref. [1] for which either no XL at all, or intra-protein XL ("IntraXL") only, were detected.

In ref. [1] we performed relative protein quantitation experiments for pairs of virion preparations considered orthogonal in preparation methodology, leading to histograms of protein abundance ratio (x) vs. number of proteins (y). The central portions of these histograms (within a specified distance of 1:1 abundance ratio) were considered to represent bona fide packaged proteins and proteins whose ratios consistently fell within this central portion are denoted "Inside", in the table below). Proteins with a quant ratio falling outside this portion of histograms (ie. with highly skewed quant ratios) in multiple protein quant experiments (denoted "Outside" in the table below) were formally considered to be protein contaminants in virion preparations. A minimal number of these was included in our XLDB if biologically interesting, to cover the possibility they may be 'loosely' packaged. Proteins found 'outside' the packaged region in just one out of five quant experiments are denoted "inconsistent" below.

		Virion quant proteome		In XLDB	XL detected		
Row	Protein	Detected	'Packaged' portion of histograms		None	IntraXL only	In S1 Fig
1	E5	N	-	Y			Y
2	F14.5	N	-	Y			Y
3	G5	N	-	Y			Y
4	O3	N	-	Y			Y
5	VENV	N	-	Y			Y
6	I2	N	-	Y	X		N
7	A14	Y	Inside	Y	X		N
8	D2	Y	Inside	Y		X	Y
9	SODL	Y	Inside	Y		X	Y
10	A9	Y	Outside	Y		X	Y
11	A46	Y	Outside	Y		X	Y
12	A11	Y	Outside	Y			Y
13	A19	Y	Outside	Y			Y
14	ATI	Y	Outside	Y			Y
15	E3	Y	Outside	Y			Y
16	GLRX2	Y	Outside	Y			Y
17	H2	Y	Outside	Y			Y
18	J5	Y	Outside	Y			Y
19	M1	Y	Outside	Y			Y
20	N1	Y	Outside	Y			Y
21	DUT	Y	Inconsistent	N	-	-	-
22	E2	Y	Inconsistent	N	-	-	-
23	F12	Y	Inconsistent	N	-	-	-
24	A49	Y	Inconsistent	N	-	-	-
25	VLTF2	Y	Inconsistent	N	-	-	-

Table B. Partial and complete X-ray crystallographic structures covering the crosslinked portions of proteins in the XL search database. Black: Vaccinia proteins. Green: Orthologous proteins from other orthopoxviruses. Gray: Structural data not used since XL not found in structurally-defined regions.

Protein	PDB	Coverage	Publication
A14_VACCW	4N8V	10%	Liu J, Xiao Z, Ko H.L., Shen M, and Ren E.C. 2014. Activating killer cell immunoglobulin-like receptor 2DS2 binds to HLA-A*11. PNAS **111 (7)** 2662-2667.
A27_VACCW	3VOP, 3U59	58%, 9%	Chang T.H., Chang S.J., Hsieh F.L., Ko T.P., Lin C.T., Ho M.R., Wang I, Hsu S.T.D., Guo R.T., Chang W, Wang A.H.J. 2013. Crystal Structure of Vaccinia Viral A27 Protein Reveals a Novel Structure Critical for Its Function and Complex Formation with A26. Plos Pathog 9 e1003563-e1003563.<br><br>Kaever T, Matho M.H., Meng X, Crickard L, Schlossmn A, Xiang Y, Crotty Y, Peters B, Zajonc D.M. 2016. Linear Epitopes in the Vaccinia Virus A27 Are Targets of Protective Antibodies induced by Vaccination against Smallpox. J. Virol **90** 4334-4345.
A46_VACCW	4LQK	60%, 35%	Fedosyuk S, Grishkovskaya I, de Almeida Ribeiro E, Skern T. 2014. Characterization and Structure of the Vaccinia Virus NF-kappa B Antagonist A46. J. Biol. Chem **289** 3749-3762.<br><br>Fedosyuk S, Bezerra G.A, Radakovics K, Smith T.K., Sammito M, Bobik N, Round A, Ten Eyck L.F., Djinovic-Carugo K, Uson I, Skern T. 2016. Vaccinia Virus Immunomodulator A46: A Lipid and Protein-Binding Scaffold for Sequestering Host TIR-Domain Proteins. PLoS Pathog 12 e1006079-e1006079.
CAHH_VACCW	4E9O	88%	Matho M.H., Maybeno M, Benhnia M.R., Becker D, Meng X, Xiang Y, Crotty S, Peters B, Zajonc D.M. 2012. Structure and Biochemical Characterization of the Vaccinia Virus Envelope Protein D8 and Its Recognition by the Antibody LA5. J Virol **86** 8080-8058.
DUSP_VACCW	3CM3, 2Q05, 2RF6	100%	Koksal A.C., Nardozzi J.D., Cingolani G. 2009. Dimeric Quaternary Structure of the Prototypical Dual Specificity Phosphatase VH1. J. Biol. Chem **284** 10129-10137
GLRX2_VACCW	2G2Q	100%	Su H.P., Lin D.Y., Garboczi D.N. 2006. The structure of G4, the poxvirus disulfide oxidoreductase essential for virus maturation and infectivity. J. Virol **80** 7706-7713.
H3_VACCW	5EJ0	73%	Singh K, Gittis A.G., Gitti R.K., Ostazeski S.A., Su H.P., Garboczi D.N. 2016. The Vaccinia Virus H3 Envelope Protein, a Major Target of Neutralizing Antibodies Exhibits a Glycosyltransferase Fold and Binds UDP-Glucose. J. Virol **90** 5020-5030.
L1_VACCW	1YPY, 2I9L, 4U6H	74%	Su H.P., Garman S.C., Allison T.J., Fogg C, Moss B, Garboczi D.N. 2005. The 1.51 -Angstrom structure of the poxvirus L1 protein, a target of potent neutralizing antibodies. PNAS **102** 4240*4245.<br><br>Su H.P., Golden J.W., Gittis A.G., Hooper J.W., Garboczi D.N. 2007. Structural basis for the binding of the neutralizing antibody, 7D11, to the poxvirus L1 protein. Virology **368** 331-341.<br><br>Kaever T, Meng X, Matho M.H., Schlossman A, Li S, Sela-Culang I, Ofran Y, Buller M, Crump R.W., Parker S, Frazier A, Crotty S, Zajonc D.M., Peters B, Xiang Y. 2014. Potent neutralization of vaccinia virus by divergent murine antiboies targeting a common site of vulnerability in L1 protein. J. Virol **88** 11339-11355.
MCE_VACCW	1AV6	100%	Hodel A.E., Gershon P.D., Shi X, Quiocho F.A. 1996. The 1.85 A structure of vaccinia protein VP39: a bifunctional enzyme that participates in the modification of both mRNA ends. Cell (Cambridge,Mass.) **85** 247-256.
MCEL_VACCW	4CKB	100%	Kyrieleis O.J.P., Chang J, De La Pena M, Shuman S, Cusack S. 2014. Crystal Structure of Vaccinia Virus mRNA Capping Enzyme Provies Insights Into the Mechanism and Evolution of the Capping Apparatus. Structure **22** 452.
MCES_VACCW	4CKB	100%	Kyrieleis O.J.P., Chang J, De La Pena M, Shuman S, Cusack S. 2014. Crystal Structure of Vaccinia Virus mRNA Capping Enzyme Provies Insights Into the Mechanism and Evolution of the Capping Apparatus. Structure **22** 452.
PAP1_VACCW	2GA9, 3ERC	98%, 100%	Moure C.M., Bowman B.R., Gershon P.D., Quiocho F.A. 2006. Crystal structures of the vaccinia virus polyadenylate polymerase heterodimer: insights into ATP selectivity and processivity. Mol Cell **22** 339-349.<br><br>Li C, Li H, Zhou S, Sun E, Yoshizawa J, Poulos T.L., Gershon P.D. 2009. Polymerase Translocation with Respect to Single-Stranded Nucleic Acid: Looping or Wrapping of Primer around a Poly(A) Polymerase. Structure **17** 680-689.
TOP1_VACCW	1VCC, 1A41	25%, 75%	Sharma A, Hanai R, Mondragon A. 1994. Crystal structure of the amino-terminal fragment of vaccinia virus DNA topoisomerase I at 1.6 A resolution. Structure **2** 767-777.<br><br>Cheng C, Kussie P, Pavletich N, Shuman S. 1998. Conservation of structure and mechanisms between eukaryotic topoisomerase I and site-specific recombinases. Cell (Cambridge,Mass.) **92** 841-850.
VE03_VACCW	1OYI	43%	Kahmann J.D., Wecking D.A., Putter V, Lowenhaupt K, Kim Y.-G., Schmieder P, Oschkinat H, Rich A, Schade M. 2004. The solution structure of the N-terminal domain of E3L shows a tyrosine conformation that may explain its reduced affinity to Z-DNA in vitro. PNAS **101** 2712-2717.
GLRX1_ECTVM	2HZE [Ectromeila]	100%	Bacik J.P., Hazes B. 2007. Crystal Structures of a Poxviral Glutaredoxin in the Oxidized and Reduced States Show Redox-correlated Structural Changes. J Mol Bio **368** 1545-1558.
PROF_MONPZ	4QWO [Monkeypox]	100%	Minasov G, Shuvalova L, Dubrovska I, Flores K, Grimshaw S, Kwon K, Anderson W.F. 1.52 Angstrom Crystal Structure of A42R Profilin-like Protein from Monkeypox Virus Zaire-96-I-16. To be published.

Table C. Numbers of crosslinking ions detected for the three predicted fragments of protein p4a and two predicted fragments of protein p4b. Bold red: Values charted on Y axis of Fig. 5b.

Line		Fragment 1	Fragment 2	Fragment 3
	**P4a**			
1	#Unique XL ions	437	13	287
2	Line #1 x DFscore for each ion	2706	30	1331
3	fragment length (aa)	614	83	194
4	**Line #2 normalized to fragment length**	**4.41**	**0.36**	**6.86**
5	#Lys plus fragment N-terminus	36	3	17
6	**Line #2 normalized to line 5**	**75.17**	**10**	**78.29**
	**P4b**			
1	#Unique XL ions	4	375	-
2	Line #1 x DFscore for each ion	4	1358	-
3	Fragment length (aa)	61	582	-
4	**Line #2 normalized to fragment length**	**0.066**	**2.33**	-
5	#Lys plus fragment N-terminus	3	28	-
6	**Line #2 normalized to line 5**	**1.33**	**48.5**	-

Table D. EFC interactions noted previously [2] and in the current study. Column 3: Previously known interactions, with those confirmed by crosslinking in red font. Column 4: All transmembrane ("TM") protein crosslinking partners for EFC proteins, with EFC partners indicated in red font. Four EFC proteins (A16, G9, J5 and L1) are known to be myristoylated [3].

**Protein**	**Comment** (ref. [2])	**Known interaction** (ref. [2])	**XL partners (TM)**
A16	Paralog of G9, J5	Binds G9	ATI
A21		-	CAHH
A28		Binds H2	H3
F9	Structurally related to L1	-	J5
G3		Binds L5	L5 (DFscore=10)
G9	Paralog of A16, J5	Binds A16	ATI, H2, H3, (A26).
H2		Binds A28	ATI, G9
J5	Paralog of A16, G9	-	ATI, F9
L1	Structurally related to F9	-	-
L5		Binds G3	H3, G3 (DFscore=10).
O3			H3

Table E. Top DFscoring inter-protein XL in the dataset, ordered by descending DFscore. Acc, Pos, #PepSeqs, #ions, BR: Accession, position in protein sequence, number of distinct peptide sequences in which the XL was detected, number of distinct ions via which it was detected, and biological rationality (Y/N), respectively. An XL is defined as a unique combination of Acc1/Pos1 and Acc2/Pos2.

Acc1	Acc2	Pos1	Pos2	#pepSeqs1	#pepSeqs2	#ions	DFscore	BR
P4B_VACCW	P4A_VACCW	563	876	3	4	43	475	Y
P4B_VACCW	P4A_VACCW	407	736	2	2	18	69	Y
P4B_VACCW	P4A_VACCW	393	736	2	2	17	46	Y
H3_VACCW	A27_VACCW	224	98	4	4	13	35	Y
P4A_VACCW	P4B_VACCW	788	568	2	2	14	34	Y
RAP94_VACCW	NTP1_VACCW	177	175	2	1	8	30	Y
A17_VACCW	H3_VACCW	36	224	4	4	8	30	Y
P4A_VACCW	P4B_VACCW	876	567	2	2	7	27	Y
I1_VACCW	E8_VACCW	178	201	2	2	3	24	
VP8_VACCW	A12_VACCW	240	75	3	2	6	22	
RAP94_VACCW	RP132_VACCW	577	790	2	2	6	22	Y
I7_VACCW	H3_VACCW	364	147	2	2	6	16	
H3_VACCW	ETF2_VACCW	1	381	1	1	1	16	N
P4A_VACCW	ETF1_VACCW	736	308	1	1	7	15	
MCEL_VACCW	RP18_VACCW	136	47	2	2	4	12	Y
VP8_VACCW	A12_VACCW	240	63	2	2	5	12	
A27_VACCW	A17_VACCW	98	36	2	2	7	12	Y
G9_VACCW	MCE_VACCW	58	226	1	1	2	12	N
G3_VACCW	L5_VACCW	111	109	1	1	2	11	Y
A26_VACCW	A27_VACCW	415	48	2	2	4	11	Y
VF17_VACCW	A4_VACCW	74	274	3	3	7	11	
RP18_VACCW	RP132_VACCW	33	1117	3	3	7	11	Y
VF17_VACCW	P4A_VACCW	74	508	3	3	5	10	
VP8_VACCW	A12_VACCW	212	75	1	1	3	10	
VP8_VACCW	A12_VACCW	212	63	1	1	2	9	
A12_VACCW	VP8_VACCW	75	204	1	2	3	8	
P4A_VACCW	P4B_VACCW	876	563	1	2	3	8	Y
P4A_VACCW	P4B_VACCW	772	471	1	1	1	8	Y
P4B_VACCW	ETF1_VACCW	471	308	1	1	2	8	
H3_VACCW	A27_VACCW	147	98	1	1	2	7	Y
A12_VACCW	VP8_VACCW	88	240	1	1	3	7	
A30_VACCW	P4A_VACCW	49	876	1	1	1	7	
E8_VACCW	A12_VACCW	1	167	1	1	2	6	
RP07_VACCW	RP132_VACCW	36	979	1	1	3	6	Y
A26_VACCW	I3_VACCW	415	189	1	1	1	6	
ATI_VACCW	P4A_VACCW	572	876	1	1	2	6	
A17_VACCW	H3_VACCW	36	235	1	1	2	6	Y

Table F. Matrix of all detected interactions between transcriptosome proteins (listed on row 1) and membrane (TM plus membrane-associated) proteins (listed in column 1). Inter-protein XL are highlighted, showing DFscores. Orange highlights emphasize interactions with DFscore > 3. Summation ('+') indicates multiple XL positions for a protein pair.

	RP147	RP132	MCEL	MCE	ETF2	RAP94	L3	MCES	NTP1	ETF1	RP35	PAP1	RP22	RP18	RP30	RP19	RP07	NPH2
**H3**		1+1+1		1	16	2	1+1				1+1			1	5+1			
**A16**	1	1	1			2	1											1
**CAHH**		1	1						1									
**ATI**		1	1			2									1			
**E8**	1	2		1						1								
**G9**	1			12									1					
**J5**					1			1										
**A11**			1		1		1											
**L5**				1														
**O3**		1																
**I5**												1						
**F9**	2+1																	
**A26**	1		3				1				1				1			
**A27**	1		1+1	1	1							1						
**VENV**					1				1									
**A21**																		
**A28**																		
**G3**			1															
**H2**	1																	
**L1**																		
**A13**																		
**A17**																		
**F14.5**																		

Table G. Matrix of all detected transcriptosome-transcriptosome protein interactions. Inter-protein XL are highlighted, showing DFscores. Other details as in Table S8.

	RP147	RP132	MCEL	MCE	ETF2	RAP94	L3	MCES	NTP1	ETF1	RP35	PAP1	RP22	RP18	RP30	RP19	RP07	NPH2
RP147		1			1+1	1+1		1	1+1+1	1					3			
RP132	1		1		1	22+1				1	2+1			11			6	
MCEL		1			1				1+1					12		1		
MCE							2											
ETF2	1+1	1	1						1	3				1				
RAP94	1+1	22+1					1		30+2+1						2+1			1
L3				2		1						1						1
MCES	1											1						
NTP1	1+1+1		1+1		1	30+2+1				1								
ETF1	1	1			3				1				2	1				
RP35		2+1																
PAP1							1	1						1				
RP22										2								
RP18		11	12		1					1		1						
RP30	3					2+1												
RP19			1															
RP07		6																
NPH2						1	1											

Table H. Crosslinked peptides in the dataset spanning AG| sites known to be cleaved during virion maturation. "Row": Row of S1 Table A. 'Pos': Crosslinking position. "XLSE": Crosslink search engine. Red font (columns 2 and 4): Virion proteins known to be proteolytically processed. Columns 6 and 7, underlines/red font: Known AG| cleavage sites. Green font: Crosslinked residues.

Row	Prot1	Pos1	Prot2	Pos2	XL pep1	XL pep2	Expt#	XLer	Cleavage	XLSE
1881	A17	22	A17	23	YYNMLDDFSAGAGVLDKDLFTEE	YYNMLDDFSAGAGVLDKDLFTEE	67	EDC	Tryp/GluC	Kojak
1882	A17	22	A17	11	YYNMLDDFSAGAGVLDKDLFTEE	YYNMLDDFSAGAGVLDKDLFTEE	67	EDC	Tryp/GluC	Kojak
1883	A17	22	A17	27	YYNMLDDFSAGAGVLDKDLFTEE	YYNMLDDFSAGAGVLDKDLFTEE	67	EDC	Tryp/GluC	Kojak
1884	A17	22	A17	21	YYNMLDDFSAGAGVLDKDLFTEE	YYNMLDDFSAGAGVLDKDLFTEE	67	EDC	Tryp/GluC	Kojak
2962	A17	180	F8	1	SINVTIPEQYTCNKPYTAGNK	MEGSK	17	DSS	Trypsin	PP
4092	A17	180	A17	187	SINVTIPEQYTCNKPYTAGNKVDVDIPTFNSLNTDDY	SINVTIPEQYTCNKPYTAGNKVDVDIPTFNSLNTDDY	17,50,51	DSS	Trypsin, Tryp/GluC	Kojak(3)
4296	A17	180	A17	180	SINVTIPEQYTCNKPYTAGNK	SINVTIPEQYTCNKPYTAGNK	17	DSS	Trypsin	ECL2
980,1012	VP8	34	VP8	36	YDDLQMVIAGAKSKFPR	YDDLQMVIAGAKSKFPR	57,69	DSS	Trypsin, Tryp/GluC	Kojak(4)
3633	A12	41	A12	61	NLLAQIGGDAAVKGGNNNLNSQTDVTAGACDTKSK	NLLAQIGGDAAVKGGNNNLNSQTDVTAGACDTKSK	25	DSS	Trypsin	ECL2
3609	G7	1	G7	240	MAAEQR	GIDTSNNIAEYIAGLKIEEIEKVEK				ECL2
632	G7	240	ETF1	197	YIAGLKIE	IISRGKK	57	DSS	Trypsin	xProphet
4009	G7	240	J1	83	KGIDTSNNIAEYIAGLKIEEIEK	KLFNKVPIVTDGR	30,51	DSS	Trypsin	PP(2)

Table I. Parameters used with crosslink search engines.

	PP	pLINK	Kojak	xQuest	ECL	ECL2
Instrument fragmentation method	HCD	HCD	HCD	CID	CID	CID
Selected instrument method type	'ESI-Q-high-res'	'HCD' From instrument.ini	'"O" for Orbitrap'	-	-	-
Format conversion to	mgf	mgf	mzML	mzXML	mzXML	mzXML
Search DB	Vaccinia86 + xDecoy	Vaccinia86	Vaccinia86 + xDecoy	Vaccinia86 + xDecoy	Vaccinia86	Vaccinia86 + xDecoy
Cleavage specificity	From program menu	From enzyme.ini file	Based on params provided	From xquest.def file	Trypsin only	Trypsin only
Allowed missed cleavages: Up to	2	2	2	2	2	2
Fixed mods	Carbamidomethly C/none *	Carbamidomethly C/none *	Carbamidomethly C/none *	Carbamidomethly C/none *	Carbamidomethly C/none *	Carbamidomethly C/none *
Variable mods	Oxidation M, Deamidation NQ	Oxidation M, Deamidation NQ	Oxidation M, Deamidation NQ	None	None	Oxidation M, Deamidation NQ
Parent tolerance (ppm)	15	15	15	10	10	10
fragment tolerance	25 ppm	25 ppm	25 ppm	0.5 Th	0.5 Th	0.5 Th
Max #varmods per peptide	2	2	2	2	None	2
Crosslinker DSS or BS3	DSS isotopic pair with a 12 Da shift	from xlink.ini	configuration file	"Xmm.def" and "xquest.def"	Entered manually	Entered manually
Other crosslinkers	Imputed as user defined parameters	from xlink.ini	configuration file	"Xmm.def" and "xquest.def"	Entered manually	Entered manually
Ion charge state	-	-	-	"Xmm.def" and "xquest.def"	Parameter.def**	Parameter.def**
Post-processing	-	-	Percolator	xProphet	Percolator	Percolator

** For samples with iodoacetamide/no treatment*
*** 'ms1 charge', 'common ion charge' and 'xlink ion charge' were adjusted manually for highest scores.*

Vaccinia86 = FASTA formatted search DB containing 86 Vaccinia protein sequences
xDecoy = Shuffled decoy database generated by xQuest
pLINK and ECL each generated their own internal decoy DB

References

1. Ngo T, Mirzakhanyan Y, Gershon PD. Protein primary structure of the Vaccinia virion at increased resolution. J Virol. 2016.
2. Moss B. Poxvirus cell entry: how many proteins does it take? Viruses. 2012;4(5):688-707. Epub 2012/07/04. doi: 10.3390/v4050688 v4050688 [pii]. PubMed PMID: 22754644; PubMed Central PMCID: PMC3386626.
3. Condit RC, Moussatche N, Traktman P. In a nutshell: structure and assembly of the vaccinia virion. In: Maramorosch K, Shatkin J, editors. Advances in Virus Research. 66: Elsevier; 2006. p. 31–124.
